# Ion-selective conformational stabilization of a disordered repeats-in-toxin protein domain

**DOI:** 10.1016/j.bpj.2025.10.014

**Published:** 2025-10-13

**Authors:** Alana P. Gudinas, Gatha M. Shambharkar, Marina P. Chang, Daniel Fernández, Tsutomu Matsui, Danielle J. Mai

**Affiliations:** 1 Department of Physics, Stanford University, Stanford, California; 2 Department of Materials Science & Engineering, Stanford University, Stanford, California; 3 Macromolecular Structure Group, Nucleus at Sarafan ChEM-H, Stanford University, Stanford, California; 4 Stanford Synchrotron Radiation Lightsource, SLAC National Accelerator Laboratory, Menlo Park, California; 5 Department of Chemical Engineering, Stanford University, Stanford, California

## Abstract

Ion-binding intrinsically disordered proteins (IDPs) recruit and bind to specific metal ions to perform critical biological functions. In proteins where ion binding and structural transitions are coupled, interactions with off-target toxic metals can dramatically disrupt protein structure and function, exemplified by lead and mercury poisoning. Understanding the complex mechanisms underlying how IDPs exclude or allow binding to different ionic species is crucial for addressing the origins of metal toxicity in biological systems. Here, we elucidate mechanisms of ion selectivity in an IDP that adopts a structure upon Ca^2+^ binding. We probed ion-induced conformational changes of a repeats-in-toxin (RTX) protein domain in the presence of different ion ligands—Mg^2+^, Ca^2+^, Sr^2+^, and Ba^2+^—with chemical similarities but drastically different ionic radii. RTX adopts ion-selective conformations measured by x-ray crystallography, small-angle x-ray scattering, and circular dichroism. High-resolution x-ray structures reveal that Sr^2+^ induces a nearly identical RTX structure as natively binding Ca^2+^, enabled by the intrinsic flexibility and disorder of the protein. Small-angle x-ray scattering and circular dichroism indicate that smaller Mg^2+^ does not induce a significant conformational change in RTX, whereas larger Ba^2+^ induces a partially folded structure. These results highlight the importance of geometric constraints imposed by protein structure in determining metal ion selectivity, yielding insights into how off-target ion binding may result in protein misfolding and malfunction.

## Graphical abstract

**Figure:**
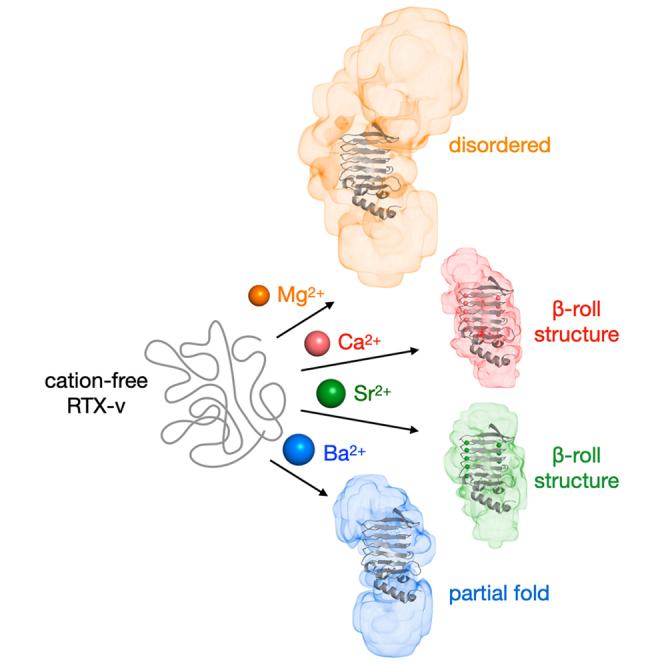

## Significance

The mechanisms of metal selectivity in ion-binding proteins are complex, which is exacerbated in the case of conformationally flexible, intrinsically disordered proteins. In this study, we elucidate the roles of ion size and protein binding site geometry in the ion selectivity of a calcium-ion-binding intrinsically disordered protein. Ion-induced structural transitions are probed using multiscale structural experiments to find that protein flexibility and disorder allow promiscuous binding to strontium ions, whereas the geometric constraints of binding sites prevent full structural formation with smaller magnesium ions or larger barium ions. Importantly, barium stabilizes partially folded structures, uncovering how off-target metal binding may induce protein misfolding and downstream biological malfunction.

## Introduction

Intrinsically disordered proteins (IDPs) adopt dynamic and heterogeneous conformations to perform critical biological functions (1,2,3). Many IDPs undergo drastic conformational changes upon binding to molecular and ionic ligands, sometimes transitioning from disordered states to folded structures (4,5). Proteins that fold upon binding to metal ions include charged sequence motifs, which have evolved selectivity for specific ionic species (6,7). In these cases, interactions with off-target ions can dramatically disrupt protein structure and function, leading to downstream biological malfunction (8). Well-studied examples include cadmium-induced protein aggregation (9), lead competition for calcium-binding sites (10), and mercury binding to cysteine residues (11). In IDPs where folding and ligand binding are coupled, off-target ion binding can promote aggregation linked to neurodegenerative disorders (12,13,14). The mechanisms underlying how IDPs exclude or allow binding to different ionic species are complex. These complexities are exacerbated when IDPs undergo structural transitions upon binding (6,15,16). To understand the coupling between ion-dependent folding and intrinsic ion selectivity, it is critical to investigate ion-induced conformational changes in IDPs.

**Figure 1:**
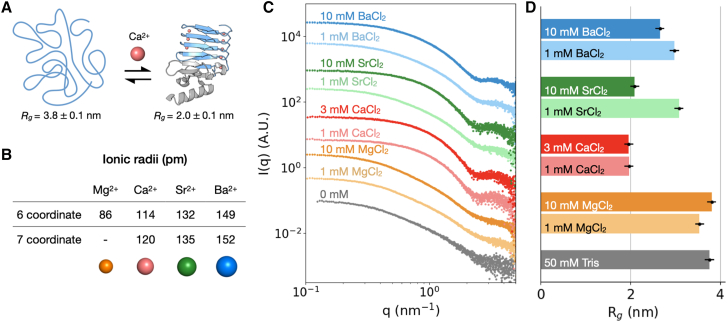
SAXS quantifies conformational changes in RTX-v upon interacting with group II ions. (*A*) RTX-v transitions from an intrinsically disordered conformation to a folded structure comprising repeats of ion-binding turns and hydrophobic β sheets. The Ca^2+^-driven conformational change is marked by a decrease in radius of gyration (*R*_*g*_). (*B*) Six- and seven-coordinate ionic radii for group II ions Mg^2+^, Ca^2+^, Sr^2+^, and Ba^2+^ (19). Ions were selected for similar chemical characteristics as Ca^2+^ and differing ionic radii. (*C*) SAXS profiles (*I*(*q*) vs. *q*) of RTX-v incubated with 1–10 mM group II metal chlorides, vertically offset for clarity. (*D*) *R*_*g*_ computed from Guinier analysis of SAXS scattering profiles. Error bars represent the order of magnitude of fitting errors, which range from 0.01 nm to 0.03 nm. Ca^2+^, Sr^2+^, and Ba^2+^ induced compaction of the protein compared with the divalent cation-free condition, whereas incubation with Mg^2+^ did not reduce RTX-v *R*_*g*_. For a Figure360 author presentation of Figure 1, see https://doi.org/10.1016/j.bpj.2025.10.014#mmc2.

A family of disordered proteins that undergo exemplary ion-induced conformational changes are repeats-in-toxin (RTX) proteins, which fold upon binding to calcium ions (Fig. 1 *A*) (17,18). RTX proteins are disordered in calcium-poor bacterial intracellular environments (100 nM Ca^2+^) and undergo calcium-dependent folding upon translocation and secretion to calcium-rich extracellular environments (10 μM to over 10 mM Ca^2+^) (20). A well-studied RTX protein is adenylate cyclase toxin (CyaA^1-1706^) from *Bordetella pertussis*, which contains 40 repeats of a Ca^2+^-binding motif described by the amino acid sequence GGXGXDXUX. In this motif, X can be any amino acid, and U is an aliphatic amino acid (21). Glycine residues confer flexibility to the protein, and aspartic acid provides negative charge to recruit cations. The repeat region forms five distinct blocks denoted with Roman numerals i to v proceeding from N- to C-terminal domains, respectively. The fifth block—denoted RTX-v (CyaA^1529-1680^)—binds most strongly to Ca^2+^ and consists of nine repeats of the consensus sequence flanked by a C-terminal capping domain. The C-terminal RTX-v binds to eight Ca^2+^ ions and initiates folding as the protein is secreted through the type I secretion system of Gram-negative bacteria. Cooperative Ca^2+^-induced folding of the entire RTX protein then proceeds successively from the C-terminus to the N-terminus (22,23,24).

The coordination environment of RTX-v is most flexible at the solvent-exposed C-terminal Ca^2+^ at position 8—denoted Ca^2+^[8]—where two water molecules assist in Ca^2+^ coordination. The remaining seven Ca^2+^ ions are periodically coordinated by backbone carbonyls and side-chain carboxyls within the ion-binding turns (GGXGXD) in a pentagonal bipyramidal geometry. Successive binding of each ion triggers partial folding of the structure and increases the Ca^2+^ affinity of the remaining sites. Ca^2+^-induced folding of the disordered protein forces hydrophobic residues (XUX) to form a β-roll structure. Without Ca^2+^, electrostatic repulsion between aspartic acid carboxylate groups drives the intrinsic disorder of RTX-v (25).

Off-target binding of CyaA to ions beyond Ca^2+^ prevents formation of the full β-roll structure and disrupts CyaA activity (26). RTX-v binds to Ca^2+^ with micromolar to millimolar affinity, showing selectivity over high background concentrations of Mg^2+^ (100 mM); however, Ca^2+^ can be replaced by other ions (26,27,28,29). In CyaA, small fractions of Ca^2+^-binding sites can be replaced with barium and cadmium, which hinders toxin activity (30).

Recently, the distinct ion-dependent conformational changes of RTX-v have been used as a proxy to measure its affinity to ions beyond Ca^2+^. Förster resonance energy transfer measurements suggest that RTX-v binds to Sr^2+^ with 10-fold weaker affinity than Ca^2+^. Interestingly, trivalent cations including rare earth elements induce a different compact structure entirely (27,31,32). It is clear RTX-v may bind to ions beyond Ca^2+^, but its ion-dependent conformational states remain poorly characterized.

We elucidate mechanisms of ion selectivity in RTX-v by probing ion-induced conformational changes in the presence of different ion ligands. We combined small-angle x-ray scattering, x-ray crystallography, and circular dichroism to study the structure of RTX-v in the presence of group II divalent cations. Mg^2+^, Ca^2+^, Sr^2+^, and Ba^2+^ were chosen for their chemical similarities but drastically different ionic radii (Fig. 1 *B*). We found that Sr^2+^ successfully replaces all Ca^2+^-binding sites in RTX-v, stabilizing a nearly identical β-roll structure. Smaller Mg^2+^ does not induce a conformational change in RTX-v, whereas larger Ba^2+^ appears to promote partially folded structures. The disorder and flexibility of RTX-v give rise to promiscuous ion binding, but its repetitive sequence restricts cooperative binding to geometrically favorable ions (33).

## Materials and methods

All materials are listed in Table S1.

### Protein preparation

RTX-v was produced using directional cloning and recombinant protein expression (28). A gene fragment encoding RTX-v was designed with an N-terminal 6×His tag to aid purification. The fragment was subcloned using BamHI and HindIII restriction sites into expression vector pQE-9. DNA and amino acid sequences are provided in the supporting material. The plasmid is available for use from Addgene (catalog #225964). Recombinant RTX-v was expressed in T7 Express lysY/I^*q*^ *Escherichia coli*. Briefly, 10 mL freshly grown overnight culture was inoculated into 1 L LB media supplemented with 10 mg L^−1^ ampicillin. Cultures were incubated at 37°C and 220 rpm. Once the optical density at 600 nm reached 0.8–1.0 (2–3 h), cultures were induced with 1 mM isopropyl-β-d-1-thiogalactopyranoside. Expression proceeded for 6 h at 37°C and 220 rpm. Cells were harvested by centrifugation at 4136 × *g* for 10 min. Pelleted cells were resuspended in 25 mL denaturing lysis buffer (8 M urea, 1.0 M NaCl, 100 mM sodium phosphate, and 10 mM Tris (pH 8.0)) and stored at −70°C.

Expressed RTX-v was recovered from cell pellets and isolated using immobilized metal affinity chromatography to capture 6×His-tagged proteins, dialysis to remove excess ions, and lyophilization to remove water. Cell pellets were defrosted with an additional 25 mL of denaturing lysis buffer and lysed by sonication. Crude lysates were clarified by centrifugation (9250 rpm for 1 h) and filtration (0.45 μm). Clarified lysates were incubated with NiNTA resin for at least 2 h at ambient temperature or overnight at 4°C. Protein-bound resins were washed with denaturing lysis buffer supplemented with 10 mM imidazole, followed by elution with 250 mM imidazole. Protein purity was assessed by sodium dodecyl sulfate polyacrylamide gel electrophoresis (Fig. S1). Pure fractions were dialyzed against chelating buffer (1 mM EGTA, 50 mM NaCl, and 10 mM Tris (pH 8.0), three exchanges) and ultrapure water (seven exchanges). Water was removed by lyophilization, and purified proteins were stored at −20°C. Protein expression yields ranged from 100 mg to 125 mg per 1 L culture.

### Small-angle x-ray scattering data collection and analysis

Ion-dependent conformations of RTX-v were obtained using size-exclusion chromatography coupled to small-angle x-ray scattering (SEC-SAXS, SASBDB: SASDXM8, SASDXN8, SASDXP8, SASDXQ8, SASDXR8, SASDXS8, SASDXT8, SASDXU8, SASDXV8, SASDXW8, SASDXX8, SASDXY8, and SASDXZ8). SEC-SAXS was conducted at the Stanford Synchrotron Radiation Lightsource (SSRL, Menlo Park, CA), beamline 4-2, with a 1.7-m sample-to-detector distance and 11-keV beam energy (wavelength *λ* = 1.127 Å) (34). Lyophilized protein was resuspended at 10 mg mL^−1^ in running buffer (5 mM dithiothreitol and 50 mM Tris (pH 7.5)) supplemented with up to 10 mM MgCl_2_, CaCl_2_, SrCl_2_, BaCl_2_, or KCl. Dithiothreitol protects protein samples from radiation damage (35). Samples were dissolved overnight at 4°C and filtered (0.22 μm) before measuring protein concentration (absorbance at 280 nm, molar extinction coefficient 18,450 M^−1^ cm^−1^). Filtered protein solutions are stable for at least 4 days.

SAXS data were collected downstream of an in-line SEC, outfitted with a Superdex 200 column equilibrated with running buffer supplemented with metal chlorides. Using a 50 μL sample injection volume (30 μL for 10 mM KCl and 3 mM CaCl_2_) and a 0.05 mL/min flow rate, scattering images were collected with a 2-s exposure every 5 s for each metal chloride condition. Data reduction and initial analyses were performed using the BL4-2 automated SEC-SAXS data processing and analysis pipeline, *SECPipe* (https://www-ssrl.slac.stanford.edu/smb-saxs/node/1860), which implements the program *SASTOOL* (https://www-ssrl.slac.stanford.edu/smb-saxs/node/1914) and ATSAS AUTORG (36). Briefly, 50 image frames in the first 100 frames were scaled and averaged to create a buffer scattering profile, which was subtracted from each subsequent profile. Scattering images were reduced to one dimension and presented as scattered intensity *I* as a function of scattering vector *q* = 4*π*sin(*θ*)/*λ*, where 2*θ* is the scattering angle, and *λ* is the x-ray wavelength. Guinier analysis using AUTORG was conducted to approximate the radius of gyration, *R*_*g*_, in each scattering profile. *R*_*g*_ is approximated as follows:

$$I\left(q\right)\approx I\left(0\right)e^{-q^{2}R_{g}^{2}/3}\text{,}$$

where *I*(0) is the intensity at *q* = 0. *R*_*g*_ and *I*(0) were extracted from linear fits to the Guinier plots limited to *qR*_*g*_ < 1.3 (37). Rg are reported as the fit values and order of magnitude of fitting errors, which range from 0.01 nm to 0.03 nm. For each SEC-SAXS elution profile, an average of five scattering profiles where *R*_*g*_ was constant were selected for further analysis. Visual inspection of the selected curves was performed using ATSAS PRIMUS (38). Automatically computed *I(q)* scale factors from PRIMUS were applied in all scattering profiles shown in this work. To independently compute *R*_*g*_, pairwise distance distributions *P*(*r*) were generated in ATSAS GNOM (Fig. S6) (39). The *D*_*max*_ value that ensured *P*(*D*_*max*_) = 0 and maximized the GNOM total quality estimate was selected. The data collection and structural parameters are summarized in Tables S3 and S4, respectively.

To visualize solution conformations of RTX-v, 3D electron density maps were reconstructed using the Density from Solution Scattering (DENSS) algorithm (40). Twenty electron density maps were generated from each SAXS profile before alignment with the Ca^2+^-bound RTX-v structure. Maps were visualized as transparent surfaces in PyMOL, colored from lowest (blue, 2*σ*) to highest (red, 15*σ*) electron densities (Figs. S4 and S5) (40).

### Protein crystallization

To resolve ion-bound protein structures, RTX-v was incubated with Mg^2+^, Ca^2+^, Sr^2+^, and Ba^2+^ during protein crystallization. Fresh protein solutions were prepared at 10 mg mL^−1^ in tris-buffered saline (TBS, 50 mM Tris, and 150 mM NaCl (pH 8)) and equilibrated overnight at 4°C. Two milliliters of protein solution was loaded onto a Superdex 75 size-exclusion chromatography column and fractionated using an Äkta pure chromatography system. Fraction purity was analyzed using sodium dodecyl sulfate polyacrylamide gel electrophoresis. Pure fractions were concentrated using Amicon spin columns (3 kDa MWCO) before crystallization screens.

Protein crystallization conditions were screened in sitting drop vapor diffusion plates. Protein solutions were supplemented with 30 mM MgCl_2_, 10 mM CaCl_2_, 10 mM SrCl_2_, or 30 mM BaCl_2_. Some precipitate was observed upon supplementing with CaCl_2_; in this case, the precipitate was removed by centrifugation, and the protein concentration in the supernatant remained unchanged. Ion-supplemented protein solutions were mixed 1:1 with crystallization screening formulations using a Douglas Oryx8 Crystallization Robot for automated screen setup. All protein crystallization screens were incubated at 16°C. RTX-v with 10 mM CaCl_2_ was incubated with Morpheus I and II and JCSG core suite I-IV screens. RTX-v with 10 mM SrCl_2_ was incubated with Morpheus I and II, JCSG core suites I and II, MCSG-4, and Shotgun I screens. Conditions with 30 mM MgCl_2_ or BaCl_2_ were incubated with MCSG-4, Memgold, Morpheus I and II, and Shotgun I screens. For MgCl_2_ and BaCl_2_ screens, incubation for over 3 months did not produce diffraction-quality crystals comprising both protein and target cations. Crystal formation was monitored using a Formulatrix RockImager2 UV-Vis Imager. For screens containing 10 mM CaCl_2_ or 10 mM SrCl_2_, approximately 10 crystallization conditions produced crystals. In general, crystals harvested from different crystallization conditions showed variation in x-ray diffracting power, and therefore, several were screened for initial data quality assessment. The best candidates were selected for high-resolution data collection.

#### RTX-v-Ca^2+^

Diffraction-quality crystals were grown from a 1:1 mixture of 7.5 mg/mL protein solution (50 mM Tris, 150 mM NaCl, and 10 mM CaCl_2_ (pH 8)) and reservoir solution (Morpheus II H12: 40 mM polyamines, 100 mM glycylglycine, and 2-amino-2-methyl-1,3-propanediol buffer, 31% v/v: 10% w/v PEG 20k; 50% w/v trimethylpropane, 2% w/v NDSB-195 (pH 8.5)). Crystals were harvested 2.5 months after setting up screening plates and immediately frozen in liquid N_2_ in a 3:1 mixture of the Morpheus II H12 reservoir solution and glycerol.

#### RTX-v-Sr^2+^

Diffraction-quality crystals were grown from a 1:1 mixture of 6.9 mg/mL protein solution (50 mM Tris, 150 mM NaCl, 10 mM SrCl_2_ (pH 8)) and reservoir solution (Morpheus I H4: 0.1 M amino acids, 100 mM imidazole and 2-(N-morpholino)ethanesulfonic acid monohydrate buffer, 37.5% v/v: 25% v/v 2-methyl-2,4-pentanediol; 25% w/v PEG 1000, 25% w/v PEG 3350 (pH 6.5)). Crystals were harvested 1.5 months after setting up the screens and immediately frozen in liquid N_2_ in a 3:1 mixture of the Morpheus I H4 reservoir solution and glycerol.

### X-ray data collection, structure solution, and refinement

Data for single Ca^2+^-bound and Sr^2+^-bound protein crystals were collected at SSRL beamlines 9-2 and 12-2, respectively (41). Data collection parameters and structure refinement results are detailed in Table S10.

#### RTX-v-Ca^2+^

High-resolution x-ray data were collected for the Ca^2+^-bound RTX-v crystal to a Bragg spacing of 1.70 Å (PDB: 9P0C). Data were integrated using *XDS* (42) and scaled with AIMLESS (43). The crystal belonged to the monoclinic space group P2_1_ and contained two polypeptide chains per asymmetry unit. The structure was solved by the molecular replacement method with Phaser (44), using the Block V RTX domain of the adenylate cyclase toxin from *Bordetella pertussis* (PDB: 5CXL (18)). The search model included the polypeptide chain stripped of nonprotein atoms. Residues 1513–1684 (chain A) and 1514–1682 (chain B) were unambiguously traced in the electron density maps. Extra electron density was attributed to residues in the N-terminal 6×His tag, which coordinated zinc ions. Additional peaks in electron density were detected and assigned to eight bound Ca^2+^ ions. Throughout refinement with REFMAC5 (45), manual adjustments of the polypeptide chain were made in COOT (46). Solvent water molecules, glycerol, and a chloride ion were assigned. Water molecules were placed based on their hydrogen bonding properties. Refinement continued until convergence of R_work_ and R_free_ and reached an agreement between the model and experimental data. Throughout this manuscript, the chain A polypeptide is referenced as RTX-v-Ca^2+^.

#### RTX-v-Sr^2+^

High-resolution x-ray data were collected for the Sr^2+^-bound RTX-v crystal to a Bragg spacing of 1.50 Å using a 15 × 15 μm microfocused beam (PDB: 9P0D). Data were integrated using DIALS (47) and scaled with SCALA (48). The presence of strontium in the sample was detected by employing an excitation scan to measure the fluorescence counts from any element present in the sample with an excitation energy of 16.605 KeV. A fluorescence peak at 14.100 keV was detected, confirming strontium in the sample. To use this signal to solve the structure via experimental phasing, a multiwavelength anomalous diffraction (MAD) scan was performed to define the energies for MAD data collection at the strontium peak. Energy values using calculated anomalous scattering factors from the fluorescence data were selected with the program *autochooch* implemented in Web-Ice (49). The f” and f’ values were obtained as well as the suggested peak (maximum f”), inflection (minimum f’), and remote (high f” and f’) energies for MAD data collection. MAD data were collected at energies 16.120 (peak), 16.112 (inflection), and 9.000 (remote) KeV. The anomalous signal was significant at 2.27 Å. Data at the strontium peak were sufficient for structure solution. The CRANK2 (50) automated pipeline (via programs SFtools, PEAKMAX, SHELXC/D/E (51), REFMAC5 (45), MAPROT (52), Solomon (53), Multicomb, Parrot (46), and Buccaneer (54)) within the CCP4 (55) suite using combined iterative model building with density modification with phased refinement led to an almost completely built model (152 residues out of 156; R-factor/R_free_: 0.36/0.39). The model was completed by manual adjustments of the polypeptide chain in COOT (46). A REFMAC-SAD substructure refinement was used to determine the occupancy of the strontium ions. In the final stages of refinement, their occupancies were fixed (the occupancies range from 0.65 to 1.00). The crystal belonged to the orthorhombic space group I222 and contained one polypeptide chain per asymmetry unit. The single polypeptide chain encompasses residues Leu1525–Asp1680. Eight copies of the strontium ion are bound to the structure making 45 contacts with protein backbone and side-chain atoms. Further ligands include tris and glycerol possibly arising from the buffer condition and cryoprotectant solution. Ions chloride and sodium and a tentatively assigned formaldehyde molecule (possibly a PEG impurity (56)) were placed at occupancy = 0.50 in special positions.

### Circular dichroism spectroscopy

Ion-dependent protein structural changes were monitored using CD spectroscopy (PCDDB: deposition in progress). Lyophilized protein was resuspended in 50 mM Tris (pH 7.5), equilibrated overnight at 4°C, filtered (0.2 μm polyethersulfone membrane), and measured using UV-vis to determine protein concentration. Protein solutions were supplemented with up to 100 mM metal chlorides, and final protein concentrations were between 10 and 13 μM. CD experiments were conducted on at least three biological replicates using a Jasco J-815 spectropolarimeter (Fig. S8). Samples were loaded into a 1-mm pathlength cuvette (Hellma) and held at 20°C. Scans were performed from 250 nm to 190 nm with 0.2-nm steps, 1-nm bandwidth, and 2-s integration times, at a scanning speed of 50 nm/min. Spectra were averaged between 10 scans, and all spectra were corrected by background subtraction of the protein-free buffer (0–100 mM metal chloride, 50 mM Tris (pH 7.5)).

Spectral deconvolution was performed from 200 nm to 250 nm with CDPro software using the reference set SPD48, which is the largest available reference set that includes denatured proteins (57). The results from CDSSTR, CONTIN/LL, and SELCON3 methods were normalized and averaged to facilitate quantitative comparisons.

## Results

### Group II ions induce nonmonotonic changes in RTX-v size and flexibility

We observe distinct behaviors in 1D scattering profiles of unfolded and folded proteins (Fig. 1 *C*, page 2). For unfolded proteins in divalent cation-free buffer, the scattering intensity decreases as a function of *q* and lacks distinct features. Similar scattering profiles are observed with up to 10 mM MgCl_2_. In comparison, the full-length CyaA^1-1706^ does not fold in response to Mg^2+^, though Mg^2+^ can occupy its ion-binding sites (30). For folded proteins in the presence of 1 mM and 3 mM CaCl_2_ (henceforth referred to as RTX-v-Ca^2+^), the scattering intensity increases in the mid-*q* region between 0.3 nm^−1^ and 2 nm^−1^ (Fig. 1 *C*). Emergent features in the high-*q* region are attributed to enhanced structural order within the folded protein (58,59).

Upon incubation with Sr^2+^ or Ba^2+^, RTX-v adopts intermediate conformations between those of the cation-free unfolded protein and Ca^2+^-bound folded protein. With 1 mM SrCl_2_ or 1 mM BaCl_2_, the scattered intensities resemble the cation-free sample, with slight enhancement of the high-*q* scattering intensity characteristic of structure formation. With 10 mM SrCl_2_, the structural features of the folded protein are fully recovered, indicated by enhanced mid-*q* scattering and high-*q* features. This folded protein state is hereafter referred to as RTX-v-Sr^2+^. With 10 mM BaCl_2_, similar features appear in the high-*q* region. However, the mid-*q* scattering intensity with BaCl_2_ is weaker than with CaCl_2_ and SrCl_2_. We attribute suppressed mid-*q* scattering and a high-*q* peak to partial folding in the presence of sufficient Ba^2+^ (60).

To quantify ion-dependent RTX-v sizes, we analyzed the low *q* regions using the Guinier approximation to compute *R*_*g*_ (Fig. S3). *R*_*g*_ ranged from 3.8 ± 0.1 nm for unfolded RTX-v in 50 mM Tris to 2.0 ± 0.1 nm for RTX-v-Ca^2+^ (Fig. 1 *D*, page 2). With 1 mM and 10 mM MgCl_2_, respective radii of 3.5 ± 0.1 nm and 3.8 ± 0.1 nm are consistent with the cation-free *R*_*g*_, indicating that RTX-v remains disordered in the presence of Mg^2+^. With 1 mM SrCl_2_, *R*_*g*_ of 3.1 ± 0.1 nm indicates partial folding of RTX-v. With 10 mM SrCl_2_, RTX-v adopts a conformation with *R*_*g*_ of 2.1 ± 0.1 nm, resembling the *R*_*g*_ of RTX-v-Ca^2+^. This resemblance reflects the similar scattering profiles between RTX-v-Ca^2+^ and RTX-v-Sr^2+^. With 1 mM BaCl_2_ and 10 mM BaCl_2_, respective radii of 3.0 ± 0.1 nm and 2.7 ± 0.1 nm indicate a distinct Ba^2+^-induced protein conformation. Intermediate *R*_*g*_ suggests that BaCl_2_ contributes to the compaction of RTX-v, but not to the extent of folded RTX-v-Ca^2+^. Inspection of the Guinier plot with 1 mM BaCl_2_ revealed aggregation of RTX-v, indicated by an upturn at low *q* (60). RTX-v aggregates in the presence of Ca^2+^ (61,62) and may be more prone to aggregation in a partially folded state. To further resolve intermediate RTX-v states, additional scattering profiles were collected at 2 mM SrCl_2_, 3 mM SrCl_2_, and 3 mM BaCl_2_ (Fig. S7). We observed similar upturns at low *q* in corresponding Guinier plots, indicating RTX-v aggregation. At these intermediate metal chloride concentrations, partially folded RTX-v appears more prone to aggregation than its fully disordered or structured states. We attribute this aggregation to solvent-exposed hydrophobic residues that are more likely to form intermolecular associations.

**Figure 2:**
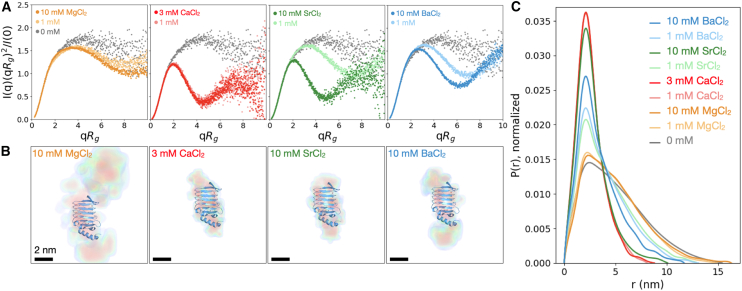
Diverse conformational states of RTX-v measured by SAXS. (*A*) Dimensionless Kratky plots for SAXS profiles shown in Fig. 1 reveal enhanced globular character in RTX-v with Ca^2+^, Sr^2+^, and Ba^2+^. (*B*) DENSS projections of electron density from SAXS profiles overlaid with the Ca^2+^-bound RTX-v crystal structure for scale. Electron density maps are colored from lowest (*blue*, 2*σ*) to highest (*red*, 15*σ*) electron densities, where *σ* represents the standard deviation of the mean electron density (40). (*C*) *P*(*r*) distributions for each SAXS profile.

Dimensionless Kratky plots reveal that RTX-v is partially flexible in its unfolded and folded states. Across all scattering profiles, an increase in *I*(*q*)*q*^2^ at high *q* is characteristic of a protein with a flexible region (Fig. 2 *A*) (63). Without divalent cations, the Kratky plot best matches a primarily disordered protein, with a shallow peak suggesting locally folded regions (58). Addition of MgCl_2_ slightly distinguishes the shallow peak while maintaining the flexible high-*q* region. In the presence of 1 mM and 3 mM CaCl_2_, a sharp peak indicates the protein is compacted upon adopting the RTX-v-Ca^2+^ structure. The same features appear with 10 mM SrCl_2_. With only 1 mM SrCl_2_, RTX-v is slightly compacted without adopting the folded structure, consistent with the trend in *R*_*g*_. Compact protein features become more pronounced at intermediate concentrations of 2 mM and 3 mM SrCl_2_. Addition of 1 mM BaCl_2_ widens the low *q* peak with respect to 1 mM CaCl_2_ and 1 mM SrCl_2_. This peak is more pronounced than in the cation-free and MgCl_2_ conditions and weaker than with Ca^2+^ or 10 mM SrCl_2_. The peak magnitude and sharpness are enhanced with 3 mM or 10 mM BaCl_2_, indicating partial compaction of the protein (63). The partial compaction of RTX-v with 1–3 mM SrCl_2_ or 1–10 mM BaCl_2_ warranted further analysis to distinguish between protein size changes and structure formation.

To further resolve the compacted protein states, we reconstructed 3D electron density maps from scattering profiles using the DENSS algorithm (40) (Fig. 2 *B*). In the absence of divalent cations, the reconstructed electron density map for RTX-v indicates a flexible and disordered conformation (data not shown). The electron density maps remain spatially extended upon adding 1 mM and 10 mM MgCl_2_. With 1 mM CaCl_2_ or 10 mM SrCl_2_, the electron density is consistent with the slightly elongated shape of the protein crystal structure. An elongated shape is expected from the repetitive structure of RTX-v-Ca^2+^ (18,64). The electron density with 10 mM BaCl_2_ is more elongated than RTX-v-Ca^2+^ with a similar width, suggesting partial β-roll formation while part of the protein remains disordered. All *R*_*g*_ values computed directly from DENSS reconstructions agree with the Guinier fits (Table S2).

Pair-distance distribution functions *P*(*r*) of intermediate RTX-v conformations show comparable length scales to folded proteins while retaining an extended and flexible region (Fig. 2 *C*). Unfolded RTX-v with no divalent cations, 1 mM, or 10 mM MgCl_2_ produces a broad pair-distance distribution with a large maximum protein dimension *D*_*max*_ (15.4 nm, 14.9 nm, and 16.3 nm, respectively). Shallow shoulders indicate disorder (63). These shoulders disappear with 1 mM and 3 mM CaCl_2_, or 10 mM SrCl_2_ (*D*_*max*_ = 8.1 nm, 8.8 nm, and 10.1 nm). In these conditions, *P*(*r*) is more symmetric and narrow, characteristic of folded proteins. Both unfolded and folded features emerge in the intermediate RTX-v states with 1 mM SrCl_2_, or 1 mM and 10 mM BaCl_2_ (*D*_*max*_ = 13.0 nm, 13.1 nm, and 11.7 nm). In these conditions, *P*(*r*) is narrower than for disordered proteins, with a shallow shoulder and trailing tail indicating a partially extended conformation. All *R*_*g*_ values computed from *P*(*r*) agree with the Guinier fits and DENSS reconstructions (Table S2).

### Sr^2+^ stabilizes RTX-v β-roll structure

**Figure 3:**
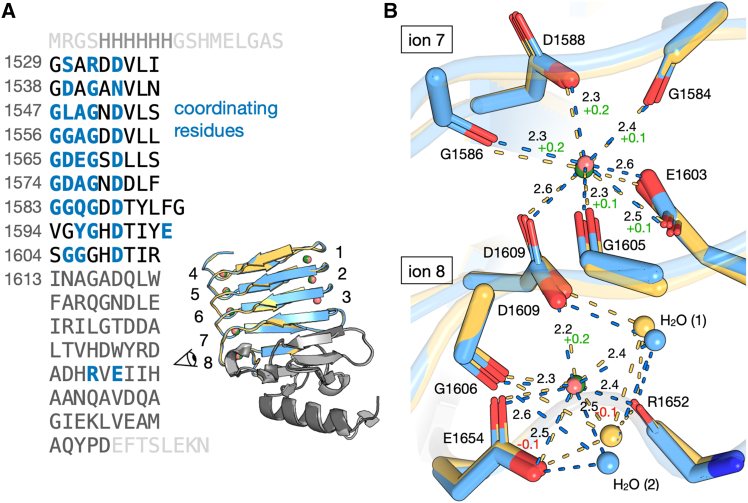
Both Ca^2+^ and Sr^2+^ stabilize the folded RTX-v structure with identical coordinating geometries. (*A*) The native RTX-v sequence and superimposed Ca^2+^-bound (*blue*) and Sr^2+^-bound (*yellow*) structures, with coordinating residues in blue font (*bolded*). The C-terminal capping domain is highlighted in gray, and the repeat ion-binding structure is shown in blue/yellow. Ca^2+^ and Sr^2+^ ions are shown as pink and green spheres, respectively. A cartoon eye indicates the viewer perspective in (*B*). (*B*) Close-up view of seventh- and eighth-position ion binding loops for the overlaid RTX-v-Ca^2+^ and RTX-v-Sr^2+^ structures. Ca^2+^ and Sr^2+^ ions are depicted with relative sizes proportional to their seven-coordinate ionic radii. Noncoordinating residues are omitted for clarity. Coordination and hydrogen bonds are shown as blue or yellow dashed lines, and the two water molecules involved in the coordination of the eighth ion are shown as blue or yellow spheres. Ion–ligand distances (Å) for the RTX-v-Ca^2+^ structure are annotated in black font. Changes in distances for the Sr^2+^-bound structures are indicated in green (increased) or red (decreased) text. Shifts in solvent molecule positions at the eighth ion site allow RTX-v to accommodate the larger Sr^2+^ ion.

X-ray structures of RTX-v-Ca^2+^ and RTX-v-Sr^2+^ are remarkably similar (Fig. 3 *A*). Structural similarity was quantified by the root-mean-square distance of 0.25 Å. In RTX-v-Sr^2+^, all eight canonical Ca^2+^-binding sites in RTX-v contained Sr^2+^ with identical coordinating ligands and geometries. Coordinating residues are highlighted throughout the RTX-v sequence in Fig. 3 *A*. Prior studies have crystallized other RTX domains with Sr^2+^ (65); to the authors’ knowledge, this structure is the first report of CyaA RTX-v bound to Sr^2+^. We assessed the quality of agreement between RTX-v-Ca^2+^ and experimental SAXS profiles using the FoXS webserver (66). The theoretical scattering profile from RTX-v-Ca^2+^ agreed well with data collected at 3 mM CaCl_2_ and 10 mM SrCl_2_ (Fig. S14). Crystallization attempts of RTX-v incubated with up to 10 mM MgCl_2_ or 10 mM BaCl_2_ did not yield diffraction-quality crystals containing both protein and cation.

For RTX-v-Sr^2+^ to accommodate the larger strontium ion, its coordinating backbone carbonyls and side-chain carboxyls shift positions (Fig. S12). At the solvent-exposed, C-terminal ion-binding site—denoted as Sr^2+^[8]—two coordinating water molecules and the side-chain carboxyls of Asp1609 and Glu1654 are rotated when compared with Ca^2+^[8]. Typically, these carboxyls and the backbone carbonyls of Gly1606 and Arg1652 bind to Ca^2+^[8] to nucleate folding of the RTX-v capping domain and stabilize the β-roll structure (18). This mechanism and structure appear to be preserved during Sr^2+^ binding, except the Asp1609 side-chain carboxyl is positioned 0.2 Å farther from Sr^2+^[8] than from Ca^2+^[8]. Conversely, the Glu1654 carboxyl and the second coordinating water molecule H_2_ O(2) both shift 0.1 Å closer to Sr^2+^[8]. Measurements of all ion-ligand distances in both structures reveal that position shifts are most pronounced at the seventh ion-binding site, where five of seven coordinating carbonyls or carboxyls are 0.1 or 0.2 Å farther from Sr^2+^[7] than Ca^2+^[7] (Fig. 3 *B*; Table S8).

**Figure 4:**
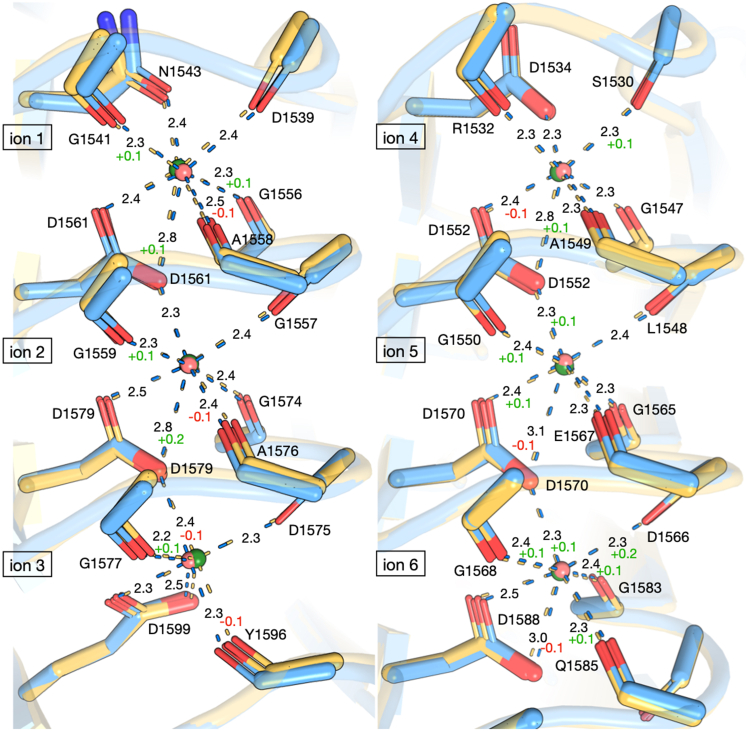
Shifts in coordinating ligand positions between the Ca^2+^-bound (*blue*) and Sr^2+^-bound (*yellow*) structures are less pronounced toward the N-terminus of the protein (*top*, ions 1 and 4). A close-up view of the first- through sixth-position ion-binding loops is shown for the overlaid protein structures. Ca^2+^ and Sr^2+^ are shown as pink and green spheres, respectively, with relative sizes proportional to their seven-coordinate ionic radii. Nucleation of ion-dependent folding at the C-terminus reduces flexibility at the N-terminus.

Most of the remaining ion-binding sites of RTX-v expand slightly to accommodate larger Sr^2+^, with less pronounced distance changes toward the N-terminal ion-binding sites (Fig. 4). The average changes in ion-ligand distances for Sr^2+^[1] to Sr^2+^[5] range from −0.02 Å to +0.03 Å, in contrast to average increases of +0.07 Å and +0.10 Å for Sr^2+^[6] and Sr^2+^[7], respectively. We attribute smaller N-terminal changes to increased protein flexibility in its unbound, disordered state, which enables larger structural shifts near the folding nucleation site of the eighth, C-terminal ion-binding site. Expanded ion-binding sites for Sr^2+^[6] and Sr^2+^[7] force some β-roll turns to be farther away from each other than with Ca^2+^. Modest displacements of turns surrounding Sr^2+^[2] and Sr^2+^[5] are measured as the distances between *α*-carbons above and below the ion site (Fig. S13; Table S9). Turns surrounding Sr^2+^[2] and Sr^2+^[5] increased by an average of 0.1 Å and 0.25 Å, respectively, compared with RTX-v-Ca^2+^ (Table S8). Less significant changes around the other ions imply fewer constraints at the edges of the β-roll structure.

We hypothesized that Sr^2+^ would nucleate folding in RTX-v due to its similarities to Ca^2+^ (67,68), but we did not anticipate such subtle expansion of ion-binding loops upon Sr^2+^ substitution. Indeed, solvent-exposed, flexible Ca^2+^-binding sites are prone to Sr^2+^ substitution (69). Ca-binding sites fitting these criteria are found in alkaline phosphatase (70), parvalbumin (71), and bacterial surface layer proteins (65), wherein Sr^2+^ successfully substitutes for Ca^2+^ with nearly identical ion-ligand coordination. Our results suggest the intrinsic flexibility of apo-RTX-v and presence of two coordinating waters enable promiscuous binding at Sr^2+^[8]. We attribute the similarity between RTX-v-Ca^2+^ and RTX-v-Sr^2+^ to the repetitive structure of RTX-v, which forces Sr^2+^ to adopt identical coordinating geometries as Ca after the nucleation of folding at the C-terminal ion-binding site. The binding site geometries are further rigidified by *δ*2 oxygens of carboxylic acids, which coordinate two ions simultaneously (72). As the protein successively folds from C- to N-termini, the repetitive sequence constrains available ion-ligand distances, forcing near-identical folds of RTX-v-Sr and RTX-v-Ca^2+^. These tight constraints may disallow RTX-v folding in the presence of ions with drastically different sizes, including Mg^2+^ and Ba^2+^.

### RTX-v secondary structure formation is tuned by ion

**Figure 5:**
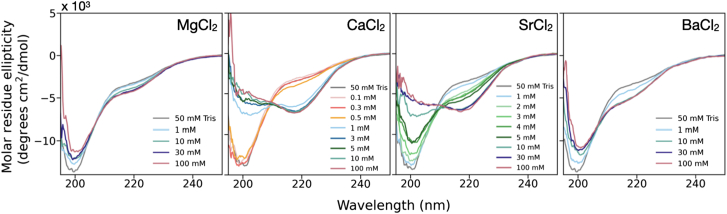
Circular dichroism experiments reveal secondary structure formation in RTX-v in response to group II ions. CD spectra were collected with RTX-v incubated with 0.1–100 mM metal chlorides. At 1 mM and 10 mM, respectively, CaCl_2_ and SrCl_2_ induce β-roll structure formation indicated by a negative peak at 218 nm. A slight shoulder between 210 and 230 nm appears upon incubation with 10 mM MgCl_2_ or BaCl_2_.

CD spectroscopy reveals a fivefold stronger affinity of RTX-v to Ca^2+^ over Sr^2+^ (Fig. 5). In the absence of divalent cations, a negative peak at 200 nm indicates a disordered protein. Upon addition of at least 1 mM CaCl_2_, RTX-v formed β-roll structures indicated by the appearance of a negative peak at 218 nm and the disappearance of the negative peak at 200 nm (17,27,73). Upon addition of at least 10 mM SrCl_2_, RTX-v underwent the same structural transition from disordered to a β-roll, with spectra matching those of RTX-v-Ca^2+^. The binding affinities of RTX-v-Ca^2+^ and RTX-v-Sr^2+^ were compared using the Hill-Langmuir equation, which determines apparent disassociation constants *K*_*D*_ and Hill coefficients *n* (Fig. S10) (74). For Ca, *K*_*D*_ of 0.69 ± 0.03 mM and *n* of 4.4 ± 0.5 agree with previous results (17,27,28). For Sr^2+^, *K*_*D*_ and *n* of 3.3 ± 0.1 mM and 1.5 ± 0.1 indicated weaker affinity with less cooperative binding.

Mg^2+^ and Ba^2+^ induce subtle structural changes in RTX-v. The disordered 200-nm peak weakened slightly upon introducing at least 10 mM MgCl_2_ or 10 mM BaCl_2_. With both ions, a shoulder emerged between 210 nm and 230 nm, which suggested β-structure formation. Spectral deconvolution quantified a modest increase in β-sheet content from 13.2% (50 mM Tris) to 16.2% (100 mM MgCl_2_) or a more dramatic increase to 21.1% (100 mM BaCl_2_) (57). Together, these features indicate that Mg^2+^ and Ba^2+^ may occupy a small number of C-terminal ion binding sites without driving the full structural transition of RTX-v.

Affinity of RTX-v to different divalent cations is heavily influenced by the distinct sizes and coordination preferences of each ion. We hypothesize the reduced affinity of RTX-v to Sr^2+^ over Ca^2+^ is partially due to the lower charge density of Sr^2+^, given its larger ionic radius than Ca^2+^ but similar coordination preferences (71,75). Mg^2+^ has a higher charge density, suggesting that aspartic acid-rich RTX-v might more easily recruit the ion, but it prefers an octahedral coordination and smaller binding sites (72,76). In Ca^2+^-binding EF-hand proteins, Mg^2+^ can occupy Ca^2+^ sites with the aid of additional coordinating waters (77,78). Given the two coordinating water molecules at Ca^2+^[8]/Sr^2+^[8], it is plausible Mg^2+^ could occupy this binding site without stabilizing the rest of the β-roll structure, since the remaining binding sites are buried and thus inaccessible to coordinating waters.

## Discussion

To gain insights into the origins of metal selectivity in disordered proteins, we characterized ion-induced conformational changes of the Ca^2+^-binding RTX-v domain. Ions beyond Ca^2+^ can occupy Ca^2+^-binding sites in RTX-v, but the mechanisms dictating how RTX-v promiscuously binds other ions are unclear (32,79). Probing conformational changes in RTX-v and other ion-binding proteins is often used as a proxy for metal binding while providing critical insight into how ions may stabilize different protein conformations (27,80). We find that RTX-v adopts diverse conformations upon incubation with group II ions with similar chemical characteristics as natively binding Ca^2+^.

We demonstrate that RTX-v adopts its native fold upon Sr^2+^ substitution by determining the atomic structure of Sr^2+^-bound RTX-v. Although RTX-v-Ca^2+^ and RTX-v-Sr^2+^ are nearly identical, RTX-v binds to Sr^2+^ with fivefold less affinity than to Ca^2+^ (Fig. S10). Slight increases in ion-ligand distances in RTX-v-Sr^2+^ are required for Sr^2+^ binding, consistent with simulations of an extracellular calcium-sensing receptor (75).

CD reveals modest secondary structure formation of RTX-v upon incubation with Mg^2+^ and Ba^2+^. Mg^2+^ did not induce compaction in the protein as measured by SAXS, whereas Ba^2+^ reduced RTX-v *R*_*g*_ compared with its disordered state. The compaction of RTX-v with 10 mM BaCl_2_ suggests that Ba^2+^ can stabilize partial folding of the protein. In contrast to the reduced *R*_*g*_ also seen with 1–3 mM SrCl_2_, a distinct mechanism underlies Ba^2+^-induced compaction, as only modest secondary structure formation was observed in CD spectra up to 100 mM BaCl_2_.

### Flexibility in apo-RTX-v enables promiscuous binding

Flexibility of the disordered apo-RTX-v enables initial binding at Sr^2+^[8], where water molecules shift positions to accommodate Sr^2+^. The repetitive structure of RTX-v then restricts binding promiscuity by constraining the available coordination geometry of the remaining binding sites. RTX-v-Ca^2+^ and RTX-v-Sr^2+^ form nearly identical structures as a result, as Sr^2+^ is forced to favor identical coordinating ligands and geometries. At the intermediate 1–3 mM SrCl_2_ concentrations, RTX-v adopts a conformation in between its disordered and folded states, suggesting a dynamic equilibrium or partially folded state (Figs. 1 *B*, 2, and S7) (81).

Conformational flexibility may allow RTX-v to partially fold in response to Mg^2+^ and Ba^2+^. SAXS and CD data reveal that partial folding may be nucleated with Mg^2+^ and Ba^2+^, evidenced by size changes and slight secondary structure formation, whereas a portion of the protein remains flexible (Fig. 2). We attribute Ba^2+^-induced compaction to direct coordination of Ba^2+^, as opposed to compaction in response to increased ionic strength. Scattering profiles of RTX-v with 10 mM KCl exactly matched that of the cation-free sample (Fig. S2). Likewise, CD spectra collected with 10 mM KCl and 10 mM NaCl matched the cation-free spectra (Fig. S9).

DENSS reconstructions of electron density maps show the compact Ba^2+^-induced conformation is more similar to RTX-v-Ca^2+^ and RTX-v-Sr^2+^ than samples with Mg^2+^. Ba^2+^-induced compaction was easily observed via a change in *R*_*g*_ of 2.7 nm versus 3.8 nm in the cation-free sample. We hypothesize that Ba^2+^ stabilizes one or more ion-binding turns at the C-terminus, but that the ion is too large for full β-roll formation. This hypothesis explains how a portion of the protein remains flexible (as seen in Kratky plots), whereas the *P*(*r*) distribution more closely resembles the folded protein structure than the disordered structure. Ba^2+^ has been shown to substitute for Ca^2+^-binding sites in EF-hand proteins with similar coordinating ligands (82).

The behavior of RTX-v with Ba^2+^ is distinct from with Mg^2+^, where very little compaction is observed in SAXS profiles, and the protein remains flexible upon incubation with MgCl_2_. DENSS reconstructions show a largely extended conformation of RTX-v with Mg^2+^, in line with a disordered structure. Prior studies suggest Mg^2+^ may occupy binding sites in RTX-v, explaining the very subtle changes in CD spectra (30,83).

## Conclusions

The factors dictating how proteins selectively bind metal ions differ widely between environmental fluctuations, protein sequence and structural constraints, and specific ionic properties (6). In IDPs that undergo ion-dependent folding, elucidating mechanisms of metal ion selectivity is especially challenging. To provide insight into how proteins coordinate ions in order to fold, we investigated ion-dependent conformations in an intrinsically disordered Ca^2+^-binding protein from the RTX family. We find that the flexibility and disorder of RTX-v promote promiscuous ion binding to Sr^2+^, which successfully substitutes all Ca^2+^-binding sites. For smaller Mg and larger Ba^2+^, the tightly constrained coordination geometry conferred from the repetitive sequence of RTX-v prevents full recovery of its folded structure. Critically, we show that Ba^2+^ induces a partially folded conformation, yielding insights into how off-target ion binding may result in protein misfolding. Many open questions remain, including quantifying Ba^2+^ binding to RTX-v and exploring sequence mutation effects on ion coordination. Sequence-to-structure prediction programs like AlphaFold3 can predict biomolecular interactions (84), but they often struggle to capture partially folded conformations in the presence of metal ions (Fig. S15). We anticipate future investigations with protein solution nuclear magnetic resonance (18,85) or molecular dynamics simulations (86,87) would provide powerful insights into partially folded conformations of RTX-v with Ba^2+^ by identifying coordinating residues. Overall, this work lays important groundwork for future studies investigating how conformational states of IDPs influence ion selectivity.

## Data and code availability

SAXS data are deposited in the Small Angle Scattering Biological Data Bank (SASBDB: SASDXM8, SASDXN8, SASDXP8, SASDXQ8, SASDXR8, SASDXS8, SASDXT8, SASDXU8, SASDXV8, SASDXW8, SASDXX8, SASDXY8, and SASDXZ8). CD data are deposited in the Protein Circular Dichrosim Data Bank (PCDDB) at pcddb.cryst.bbk.ac.uk/. The x-ray structures are available in the Protein Data Bank (PDB: 9P0C and 9P0D).

## Acknowledgments

This material is based upon work supported by the 10.13039/100000181Air Force Office of Scientific Research under award number FA9550-22-1-0241. We acknowledge training program support by the Macromolecular Structure Knowledge Center (MSKC) at Sarafan ChEM-H (RRID: SCR_023233) and the Stanford Community of Shared Research Platforms (C-ShaRP) Experiential Learning Program (RRID: SCR_022986). A.P.G. is partially supported by the 10.13039/100023581National Science Foundation Graduate Research Fellowship Program (DGE-1656518). We thank Prof. Possu Huang for access to the CD spectrophotometer and Dr. Olivia N. Pattelli for conducting crystallographic screens (Macromolecular Structure Group, Nucleus at Sarafan ChEM-H). We also thank Dr. Thomas Weiss (SSRL/SLAC), Dr. Patrick Dennis (Air Force Research Laboratory), Prof. Soichi Wakatsuki, and all members of the Mai Lab for helpful conversations.

Use of the Stanford Synchrotron Radiation Lightsource, SLAC National Accelerator Laboratory, is supported by the US Department of Energy, Office of Science, Office of Basic Energy Sciences under contract no. DE-AC02-76SF00515. The SSRL Structural Molecular Biology Program is supported by the DOE Office of Biological and Environmental Research and by the National Institutes of Health, National Institute of General Medical Sciences (P30GM133894). The contents of this publication are solely the responsibility of the authors and do not necessarily represent the official views of NIGMS or NIH.

## Author contributions

A.P.G., G.M.S., M.P.C., and D.J.M. conceptualized the project. A.P.G., G.M.S., M.P.C., D.F., and T.M. conducted the experiments. A.P.G., D.F., T.M., and D.J.M. analyzed the results. A.P.G. and D.J.M. wrote the manuscript. All authors reviewed the manuscript.

## Declaration of interests

The authors declare no competing interests.

## Supporting citations

References (88,89,90) appear in the supporting material.

## Supporting material

Document S1. Figures S1–S15 and Tables S1–S10

Fig. 360. An author presentation of Fig. 1

Document S2. Article plus supporting material
