# Supplementary material for: Ion-selective conformational stabilization of a disordered repeats-in-toxin protein domain
Source: Biophys J. 2025 Oct 13;124(23):4243–54. doi: 10.1016/j.bpj.2025.10.014 (PMC12821022; doi:10.1016/j.bpj.2025.10.014)
Supplement: Document S1. Figures S1–S15 and Tables S1–S10 [file mmc1.pdf]

Biophysical Journal, Volume *124*

Supplemental information

Ion-selective conformational stabilization of a disordered repeats-in-toxin protein domain

Alana P. Gudinas, Gatha M. Shambharkar, Marina P. Chang, Daniel Fernández, Tsutomu Matsui, and Danielle J. Mai

Supplemental Information

Ion-selective conformational stabilization of a disordered repeats-in-toxin protein domain

Alana P. Gudinas[1], Gatha M. Shambharkar[2], Marina P. Chang[2], Daniel Fernández[3], Tsutomu Matsui[4], and Danielle J. Mai[2,5,*]

[1]Department of Physics, Stanford University, Stanford, CA, USA
[2]Department of Materials Science & Engineering, Stanford University, Stanford, CA, USA
[3]Macromolecular Structure Group, Nucleus at Sarafan ChEM-H, Stanford University, Stanford, CA, USA
[4]Stanford Synchrotron Radiation Lightsource, SLAC National Accelerator Laboratory, Menlo Park, CA, USA
[5]Department of Chemical Engineering, Stanford University, Stanford, CA, USA
[*]Correspondence: djmai@stanford.edu

CONTENTS

1 Materials **2**

2 Protein preparation **3**
2.1 RTX-v DNA sequence . . . 3
2.2 RTX-v amino acid sequence . . . 3
2.3 SDS-PAGE . . . 3

3 Size exclusion chromatography with small angle X-ray scattering (SEC-SAXS) **4**
3.1 Scattering profiles with KCl . . . 4
3.2 Scattering profiles with divalent cations . . . 4
3.3 *DENSS* reconstructions . . . 5
3.4 Pair distance distribution function $P(r)$ fits . . . 6
3.5 Radius of gyration comparison across methods . . . 6
3.6 Protein aggregation during SEC-SAXS . . . 7
3.7 SEC-SAXS data collection and analysis . . . 8

4 Circular dichroism (CD) spectroscopy **12**
4.1 Triplicate data . . . 12
4.2 Monovalent cation samples . . . 13
4.3 Hill–Langmuir fits . . . 13
4.4 CD Pro . . . 14
4.5 Sample-to-sample variation . . . 15

5 X-ray Crystallography (XRC) **16**
5.1 Electron density maps . . . 16
5.2 Ligand–ion distances . . . 17
5.3 Turn-to-turn distances . . . 18
5.4 Agreement with SAXS data . . . 20
5.5 XRC data collection and refinement . . . 22
5.6 AlphaFold3 predictions . . . 23

1 MATERIALS

Table S1: Materials with vendor and catalog information.

Inorganic Salts and Buffer Reagents	Source	Identifier
Magnesium chloride hexahydrate	Thermo Fisher	CAS 7791-18-6
Calcium chloride dihydrate	Thermo Fisher	CAS 10035-04-8
Strontium chloride hexahydrate	Thermo Fisher	CAS 10025-7-4
Barium chloride dihydrate	Acros Organics	CAS 10326-27-9
Potassium chloride	Thermo Fisher	CAS 7447-40-7
Tris hydrochloride	Thermo Fisher	CAS 1185-53-1
Dithiothreitol (DTT)	Thermo Scientific	CAS 3483-12-3
**Water Purification System**		
Milli-Q Advantage A10 with BioPak Polisher, 18.2 MΩ cm	Millipore Sigma	Cat#Z00Q0V0WW
**Bacterial Strains and Competent Cells**		
NEB 5-alpha Competent E. coli (High Efficiency)	New England Biolabs	Cat#C2987H
T7 Express lysY/I^q Competent Cells	New England Biolabs	Cat#C3013I
Mix & Go! E. coli Transformation Kit and Buffer Set	Zymo Research Corporation	Cat#T3002
**Cloning**		
BamHI	New England Biolabs	Cat#R0136S
HindIII-HF	New England Biolabs	Cat#R3104S
T4 DNA Ligase	New England Biolabs	Cat#M0202L
Quick CIP	New England Biolabs	Cat#M0525S
ZymoPURE Plasmid MiniPrep Kit	Zymo Research Corporation	Cat#50-125-1483
Zymoclean Gel DNA Recovery Kit	Zymo Research Corporation	Cat#11-301
**Protein Purification and Validation**		
Nalgene™ Sterile PES Filter (0.2 μm)	Thermo Scientific	Cat#596-4520
Fisher Science Education Seamless Cellulose Dialysis Tubing (14,000 MWCO)	Thermo Scientific	Cat#S25645lH
Blue Prestained Protein Standard, Broad Range (11-250 kDa)	New England Biolabs	Cat#P7718S
HisPur™ Ni-NTA resin	Thermo Scientific	Cat#88223
Amicon® Ultra Centrifugal Filters (3 kDa MWCO)	Millipore Sigma	Cat#UFC900308
**DNA**		
Gene fragment	Twist Bioscience	N/A
pQE-9-RTXWT-C	Addgene	Cat#225964
**Crystallography**		
CrystalMation Intelli-Plates	Hampton Research	Cat#HR3-118
Morpheus I	Molecular Dimensions	Cat#MD1-47
Morpheus II	Molecular Dimensions	Cat#MD1-92
JCSG Core Suite I-IV	NeXtal	Cat#13092(4-7)
MCSG-4	Molecular Dimensions	Cat#MCSG-4
SG1 (ShotGun)	Molecular Dimensions	Cat#MD1-89
Memgold	Molecular Dimensions	Cat#MD1-41

2 PROTEIN PREPARATION

The gene encoding the RTX-v protein was flanked with restriction sites for directional cloning and purchased as gene fragments. Genes were subcloned into pQE-9 using BamHI and HindIII restriction sites, bolded in the DNA sequence below.

Protein concentration was quantified by measuring solution absorbance at 280 nm with a NanoDrop One C Spectrometer, then applying the Beer-Lambert law using a molar extinction coefficient of 18450 $M^{-1}cm^{-1}$ and a molecular mass of 19.1 kDa. Protein purity was assessed via sodium dodecyl sulfate–polyacrylamide gel electrophoresis (SDS-PAGE).

2.1 RTX-v DNA sequence

CCCCGTCACCTTTGGCTTATCAGT**GGATCC**CATATGGAGCTCGGCGCTAGCGGCAGCGCACGCGACGATGTCCTTATCGGCGATGCGGGCGCTAACGTC
CTCAACGGACTGGCTGGTAATGACGTATTATCAGGAGGGGCAGGTGACGATGTGTTATTAGGGGACGAAGGCAGTGATTTGCTGTCTGGGGATGCAGGA
AACGATGATCTGTTCGGTGGTCAGGGTGATGATACCTATCTGTTTGGGGTTGGTTACGGTCACGAC ACGATCTATGAGTCCGGCGGCGGCCACGATAC
AATCCGTATTAATGCCGGAGCAGACCAACTGTGGTTTGCGCGCCAGGGAAATGATCTTGAAATACGTATTCTCGGTACCGATGATGCGTTGACTGTTCA
TGATTGGTATCGGGACGCTGATCATAGAGTTGAAATAATTCATGC AGCGAATCAGGCTGTGGATCAAGCCGGTATTGAAAAACTGGTAGAGGCCATGG
CCCAGTACCCGGACGAATTCACTAGTCTCGAG**AAGCTT**AGATCTAGTGACATCTGGACGCTAAGACCG

2.2 RTX-v amino acid sequence

MRGSHHHHHHGSHMELGASGSARDDVLIGDAGANVLNGLAGNDVLSGGAGDDVLLGDEGSDLLSGDAGNDDLFGGQGDDTYLFGVGYGHD
TIYESGGGHDTIRINAGADQLWFARQGNDLEIRILGTDDALTVHDWYRDADHRVEIIHAANQAVDQAGIEKLVEAMAQYPDEFTSLEKLN*

2.3 SDS-PAGE

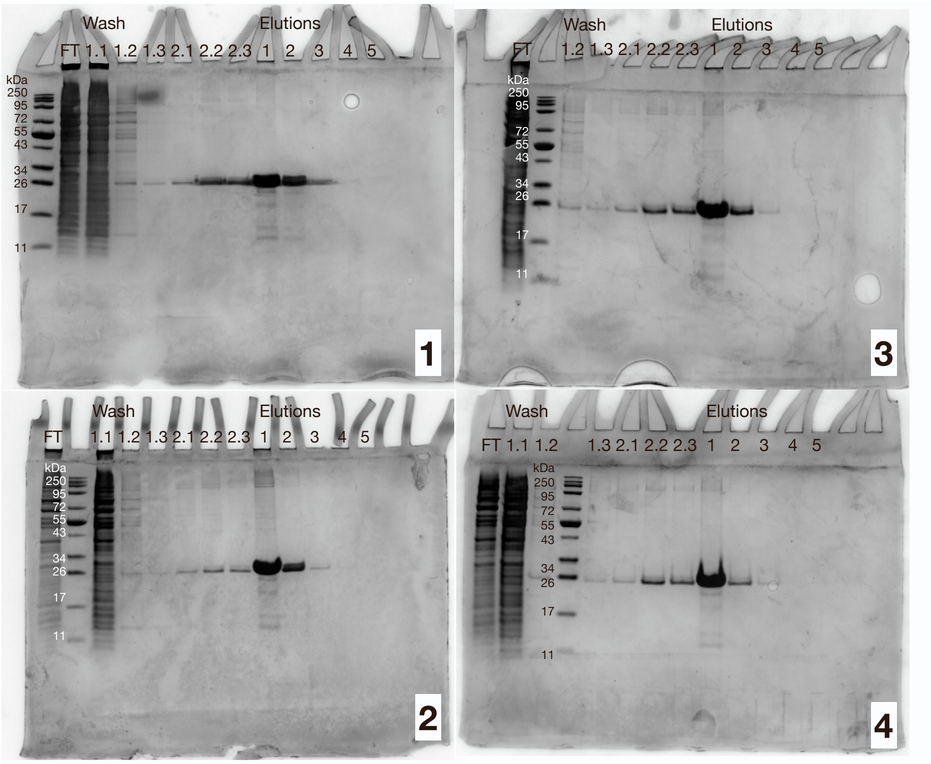

Figure S1: Representative SDS-PAGE of RTX-v fractions from flow through (FT), washes (1.1, 1.2, 1.3, 2.1, 2.2, 2.3), and elution (1-5) stages of Ni-NTA purification. Experimental conditions: 12% polyacrylamide, 200 V, 45 minutes.

3 SIZE EXCLUSION CHROMATOGRAPHY WITH SMALL ANGLE X-RAY SCATTERING (SEC-SAXS)

3.1 Scattering profiles with KCl

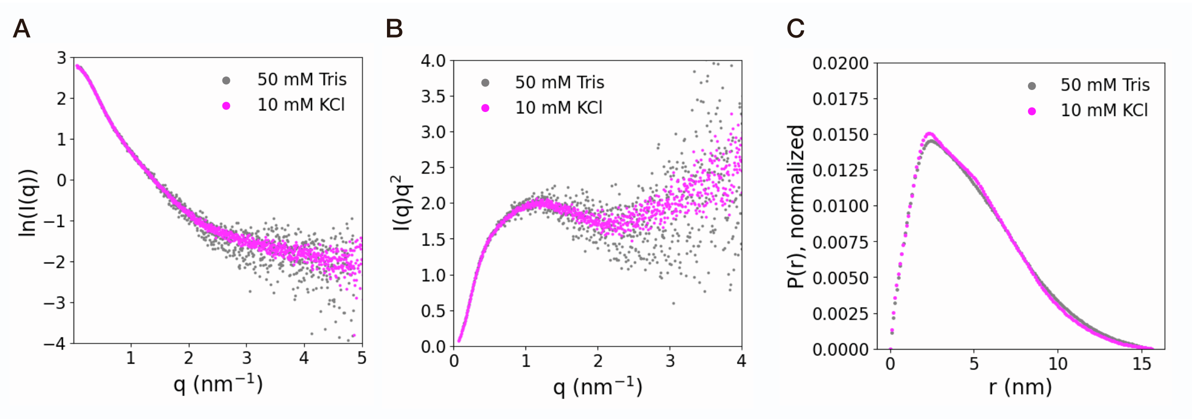

Figure S2: RTX-v exhibits identical scattering behavior when supplemented with 10 mM KCl (magenta) as in the absence of divalent cations (50 mM Tris, gray). A) Scattering profiles with 50 mM Tris, without or with 10 mM KCl. B) Kratky plots. C) $P(r)$ distributions.

3.2 Scattering profiles with divalent cations

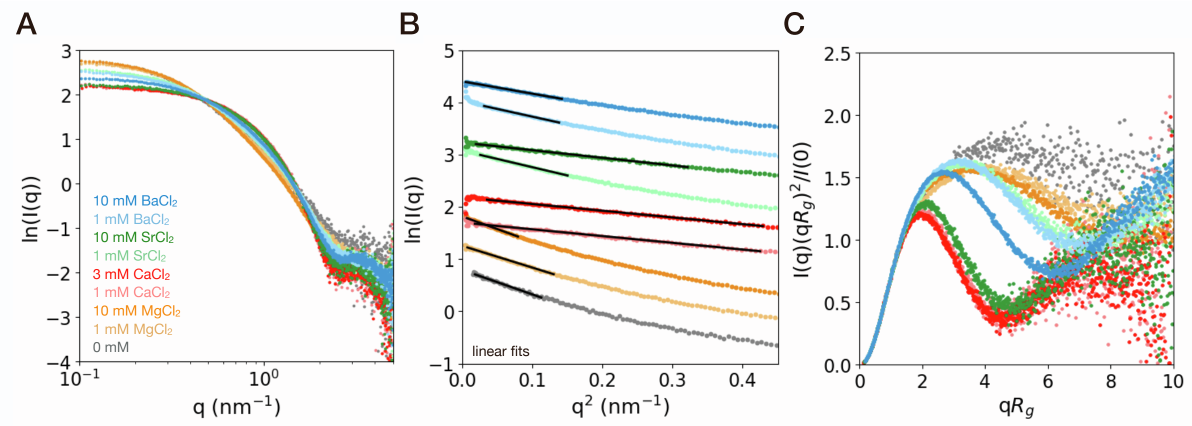

Figure S3: Scattering profiles for divalent cation conditions, overlaid to highlight differences. A) $\ln(I(q))$ vs q. Upon addition of $CaCl_2$, $SrCl_2$, and $BaCl_2$, different features emerge in the mid-q and high-q regions of the scattering profiles. The same legend is used in subsequent panels. B) Guinier plots ($\ln(I(q))$ vs q^2) with linear fits in black. Guinier analysis was applied to determine R_g and I_0 for reach sample. Steeper slopes in the low-q region of the Guinier plots correspond to larger R_g (50 mM Tris, 1 mM $MgCl_2$, 10 mM $MgCl_2$). C) Dimensionless Kratky plots for all conditions overlaid.

3.3 *DENSS* reconstructions

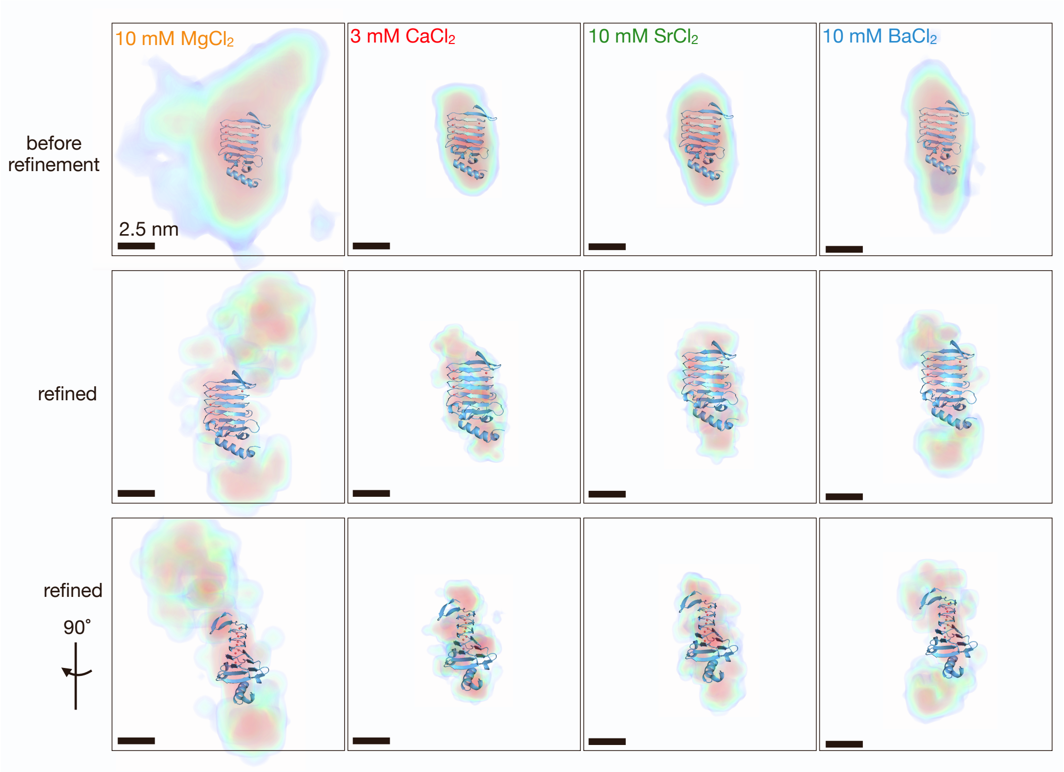

Figure S4: The *DENSS* algorithm was used to calculate 3D electron density maps from 1D SAXS data. Electron density maps are colored from lowest (blue, 2σ) to highest (red, 15σ) electron densities, where σ represents the standard deviation of the mean electron density (1). Top row: 20 *DENSS* projections were averaged and aligned with the X-ray structure of RTX-v-Ca^{2+}. Middle row: the averaged projections were refined against SAXS profiles. Bottom row: side view of refined maps, which were rotated by 90° about the vertical axis.

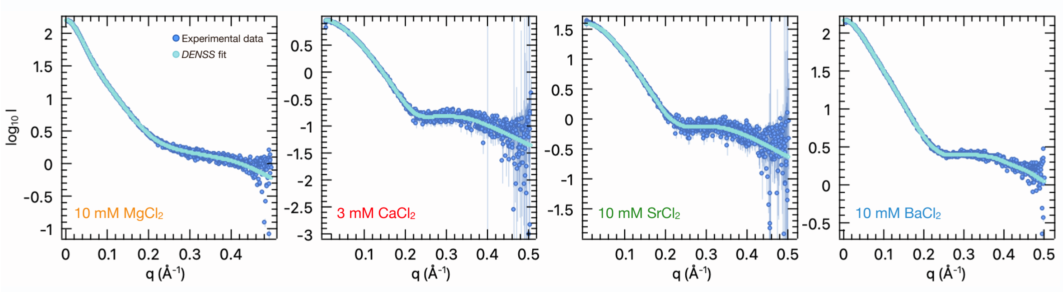

Figure S5: *DENSS* fits to experimental SAXS data. Fits to scattering data are used to refine the electron density maps.

3.4 Pair distance distribution function *P(r)* fits

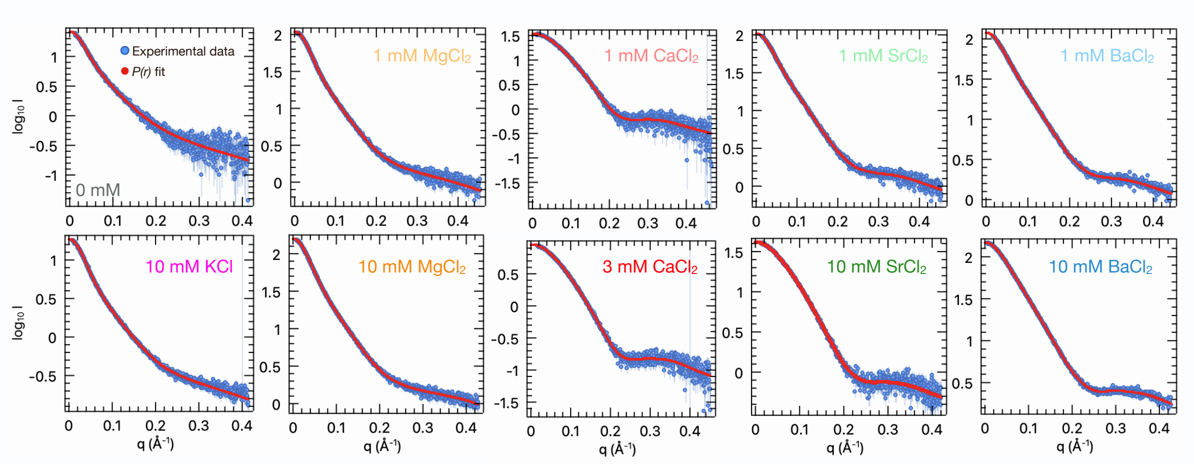

Figure S6: $P(r)$ fits to experimental SAXS data.

3.5 Radius of gyration comparison across methods

Table S2: Radii of gyration calculated by either Guinier analysis, $P(r)$, or *DENSS* reconstructions are in good agreement.

Sample	R_g (nm) Guinier	P(r)	*DENSS*
0 mM	3.8 ± 0.1	3.9	3.8 ± 0.1
1 mM $MgCl_2$	3.5 ± 0.1	3.7	3.6 ± 0.1
10 mM $MgCl_2$	3.8 ± 0.1	3.9	3.8 ± 0.1
1 mM $CaCl_2$	2.0 ± 0.1	2.0	2.0 ± 0.1
3 mM $CaCl_2$	2.0 ± 0.1	2.0	2.0 ± 0.1
1 mM $SrCl_2$	3.1 ± 0.1	3.3	3.1 ± 0.1
10 mM $SrCl_2$	2.1 ± 0.1	2.1	2.0 ± 0.1
1 mM $BaCl_2$	3.0 ± 0.1	3.2	3.1 ± 0.1
10 mM $BaCl_2$	2.7 ± 0.1	2.8	2.6 ± 0.2

3.6 Protein aggregation during SEC-SAXS

RTX-v aggregation was observed during SEC-SAXS under the following conditions: 2 mM $SrCl_2$, 3 mM $SrCl_2$, 1 mM $BaCl_2$, and 3 mM $BaCl_2$. In these cases, we determined that aggregation was not a confounding factor in extracting R_g due to agreement between R_g derived from Guinier analyses and R_g derived from $P(r)$ analyses (Table S4).

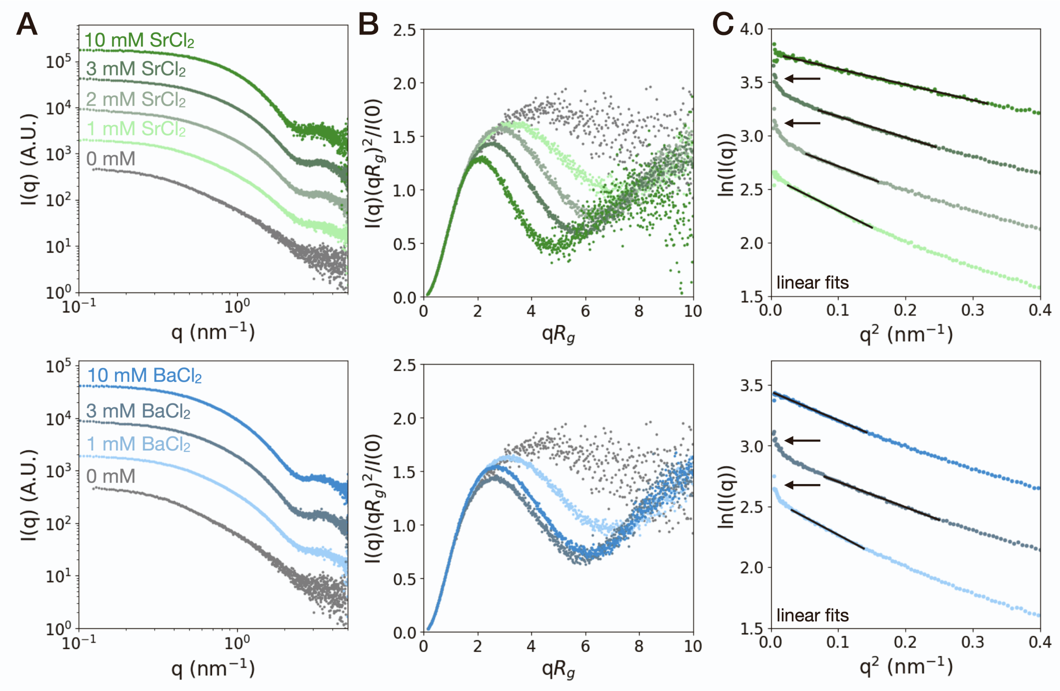

Figure S7: RTX-v adopts partially folded conformations in the presence of intermediate concentrations of $SrCl_2$ (top) and $BaCl_2$ (bottom), which induce protein aggregation. A) 1D scattering profiles. All conditions with $SrCl_2$ or $BaCl_2$ demonstrate slightly enhanced scattering in mid- and high-q regions that correspond to structure formation. Plots vertically offset for clarity. B) Kratky plots. All $SrCl_2$ and $BaCl_2$ conditions demonstrate enhanced globularity compared to the cation-free condition. C) Guinier plots with linear fits overlaid in black. An upturn in $\ln(I(q))$ at low q is characteristic of protein aggregation (indicated with arrows). Plots vertically offset for clarity.

3.7 SEC-SAXS data collection and analysis

Table S3: Data collection parameters for all SEC-SAXS samples.

**Data collection**	
Instrument	SSRL BL4-2
Type of Experiment	SEC-SAXS
Beam Current (mA)	500
Defining slits size (H mm × V mm)	0.3 × 0.30
Detector distance (m)	1.1
Detector	Pilatus3 X 1M
Beam energy (keV)	11.0
Sample cell	Quartz capillary (I*D* = ~1.3 mm)
Temperature (K)	295
Exposure time/frame (s)	2
Frames per SEC-SAXS data set	500
# of blank images used for averaging	50
# of sample images used for averaging	5
SEC column	Superdex 200 Increase 3.2/300
HPLC flow rate (mL/min)	0.05
Sample concentration (mg/ml)	10
**Software employed**	
Primary data reduction	*SasTool/SECPipe*
Data processing	*PRIMUS*
P(r) analysis	*GNOM*
ab initio modeling	*DENSS*

Table S4: Sample-specific data collection and structural parameters.

**Data collection**	0 mM	10 mM KCl	1 mM $MgCl_2$	10 mM $MgCl_2$	1 mM $CaCl_2$
q range ($Å^{-1}$)	0.012–0.487	0.007–0.507	0.006–0.505	0.007–0.502	0.00–0.505
Image #s used for averaging	345-349	345–349	335–339	345–349	385–389
SEC injection volume (μL)	50	30	50	50	50
Buffer	50mM Tris, pH 7.5	50mM Tris, 10 mM KCl, pH 7.5	50mM Tris, 1 mM $MgCl_2$, pH 7.5	50mM Tris, 10 mM $MgCl_2$, pH 7.5	50mM Tris, 1 mM $CaCl_2$, pH 7.5
**Guinier analysis**					
I(0) (arb. unit)	25.23 ± 0.15	18.24 ± 0.051	106.21 ± 0.26	156.39 ± 0.32	33.93 ± 0.068
R_g (Å)	37.59 ± 0.32	37.07 ± 0.15	35.35 ± 0.12	38.09 ± 0.16	19.68 ± 0.06
qR_g range	0.5–1.28	0.32–1.27	0.3–1.3	0.31–1.09	0.26–1.29
**P(r) analysis**					
I(0), Guinier (arb. unit)	25.90	18.55	108.40	157.40	34.20
R_g (Å), Guinier	40.39	39.45	37.78	39.69	20.45
I(0), P(r) (arb. unit)	25.91	18.55	108.40	157.40	34.20
R_g (Å), P(r)	40.68	39.56	37.87	39.8	20.47
D_{max} (Å)	154	156	149	163	81
q range ($Å^{-1}$)	0.001–0.412	0.008–0.415	0.009–0.454	0.009–0.423	0.013–0.466
Porod volume estimate ($Å^3$)	46881	49036	51334	74307	28888
DENSS					
Mode	Slow	-	Slow	Slow	Slow
# of reconstructions	20	-	20	20	20
χ^2	1.364 ± 0.035	-	1.607 ± 0.22	1.740 ± 0.36	1.318 ± 0.11
Resolution (FSC = 0.5)	36.4 ± 5.2	-	32.0 ± 7.6	35.5 ± 7.4	24.8 ± 5.2
Average R_g	37.62 ± 0.77	-	35.92 ± 0.73	37.98 ± 1.57	19.66 ± 0.22

cont. on next page

**Data collection**	3 mM $CaCl_2$	1 mM $SrCl_2$	2 mM $SrCl_2$	3 mM $SrCl_2$	10 mM $SrCl_2$
q range ($Å^{-1}$)	0.007-0.507	0.006–0.505	0.006–0.507	0.006–0.507	0.007–0.503
Image #s used for averaging	405-409	350–354	375-379	380–384	400–404
SEC injection volume (μL)	30	50	50	50	50
Buffer	50mM Tris, 1 mM $CaCl_2$, pH 7.5	50mM Tris, 1 mM $SrCl_2$, pH 7.5	50mM Tris, 2 mM $SrCl_2$, pH 7.5	50mM Tris, 3 mM $SrCl_2$, pH 7.5	50mM Tris, 10 mM $SrCl_2$, pH 7.5
**Guinier analysis**					
I(0) (arb. unit)	8.86 ± 0.016	100.96 ± 0.28	40.2 ± 0.8	42.6 ± 0.1	40.67 ± 0.089
R_g (Å)	19.64 ± 0.05	30.80 ± 0.14	26.9 ± 0.1	24.3 ± 0.1	20.92 ± 0.08
qR_g range	0.37–1.3	0.49–1.21	0.62–1.09	0.65–1.22	0.29–1.2
**P(r) analysis**					
I(0), Guinier (arb. unit)	8.95	103.10	40.73	43.76	41.22
R_g (Å), Guinier	20.52	33.06	28.37	26.50	22.36
I(0), P(r) (arb. unit)	8.95	103.20	40.74	43.76	41.22
R_g (Å), P(r)	20.58	33.32	28.61	26.70	22.43
D_{max} (Å)	88	130	115	110	101
q range ($Å^{-1}$)	0.019–0.451	0.017–0.454	0.023–0.430	0.027–0.430	0.014–0.421
Porod volume estimate ($Å^3$)	30392	46839	41148	39093	33892
DENSS					
Mode	Slow	Slow	-	-	Slow
# of reconstructions	20	20	-	-	20
χ^2	1.54 ± 0.19	1.94 ± 0.44	-	-	1.70 ± 0.21
Resolution (FSC = 0.5)	26.8 ± 4.5	29.8 ± 4.3	-	-	27.3 ± 4.8
Average R_g	19.75 ± 0.22	31.38 ± 0.93	-	-	20.29 ± 0.32

cont. on next page

**Data collection**	1 mM $BaCl_2$	3 mM $BaCl_2$	10 mM $BaCl_2$
q range ($Å^{-1}$)	0.007–0.503	0.006–0.507	0.007–0.503
Image #s used for averaging	370–374	385–389	390–394
SEC injection volume (μL)	50	50	50
Buffer	50mM Tris, 1 mM $BaCl_2$, pH 7.5	50 mM Tris, 3 mM $BaCl_2$, pH 7.5	50mM Tris, 10 mM $BaCl_2$, pH 7.5
**Guinier analysis**			
I(0) (arb. unit)	117.64 ± 0.26	37.7 ± 0.1	146.02 ± 0.18
R_g (Å)	29.78 ± 0.12	25.1 ± 0.1	26.59 ± 0.08
qR_g range	0.53–1.12	0.71–1.27	0.19–1.02
**P(r) analysis**			
I(0), Guinier (arb. unit)	118.90	38.78	147.00
R_g (Å), Guinier	31.27	27.14	27.89
I(0), P(r) (arb. unit)	118.90	38.78	147.00
R_g (Å), P(r)	31.50	27.31	27.93
D_{max} (Å)	131	108	117
q range ($Å^{-1}$)	0.018–0.444	0.029–0.430	0.001–0.429
Porod volume estimate ($Å^3$)	43570	39833	41295
DENSS			
Mode	Slow	-	Slow
# of reconstructions	20	-	20
χ^2	1.62 ± 0.12	-	3.73 ± 1.17
Resolution (FSC = 0.5)	27.7 ± 1.2	-	27.8 ± 5.0
Average R_g	30.88 ± 0.56	-	25.7 ± 1.26

4 CIRCULAR DICHROISM (CD) SPECTROSCOPY

4.1 Triplicate data

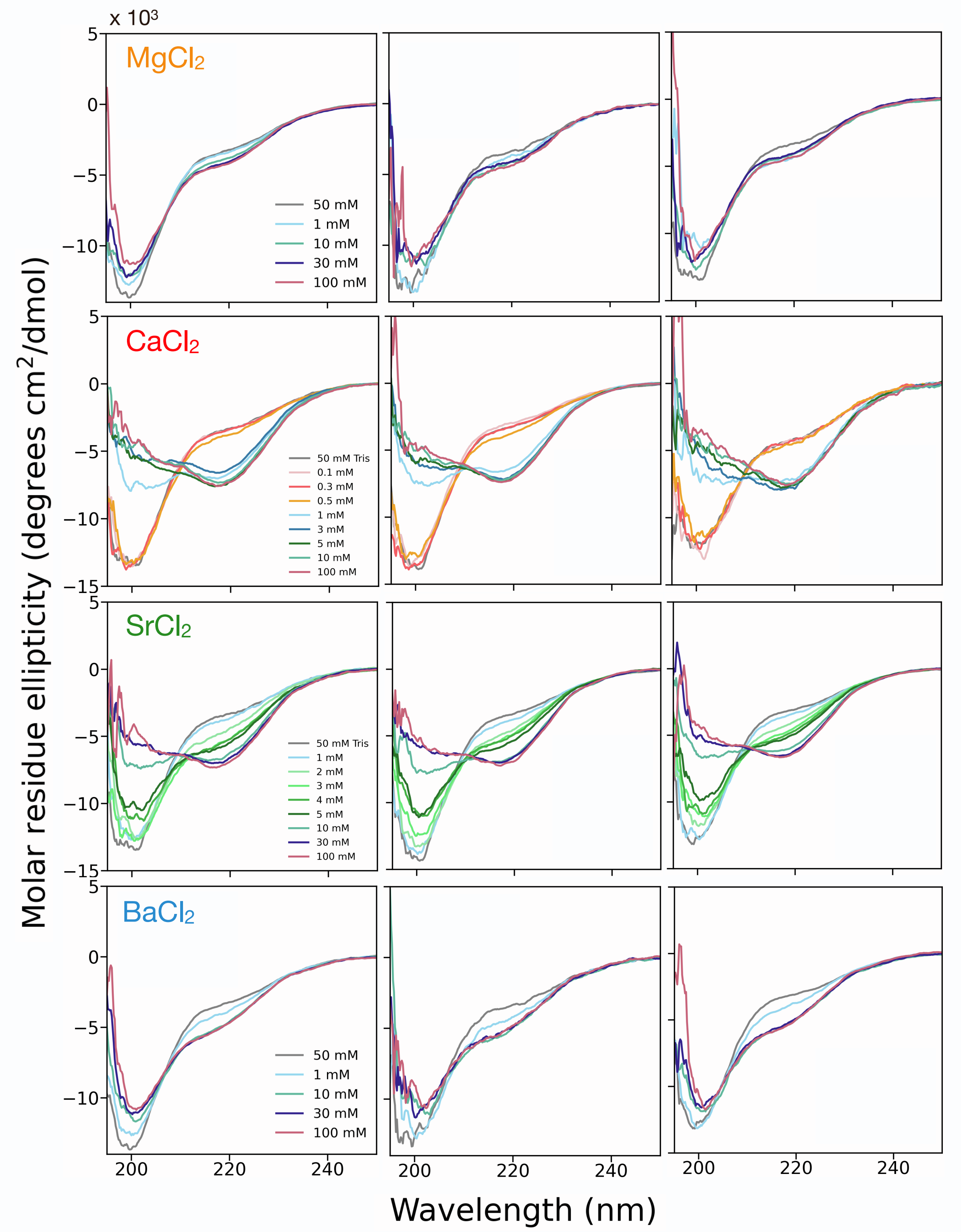

Figure S8: Triplicate CD spectra for each group II ion condition. Each column corresponds to a distinct biological sample. Different noise levels across replicates are attributed to aging of the UV lamp in the CD spectrometer.

4.2 Monovalent cation samples

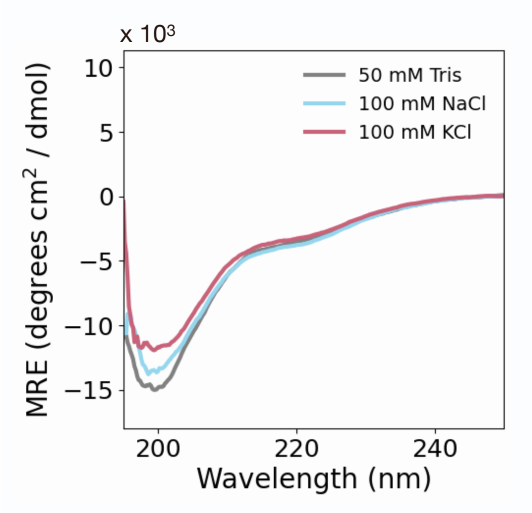

Figure S9: RTX-v does not form β-sheets in the presence of K^+ or Na^+ ions. CD spectra of RTX-v with 100 mM KCl and 100 mM NaCl overlaid with 50 mM Tris.

4.3 Hill–Langmuir fits

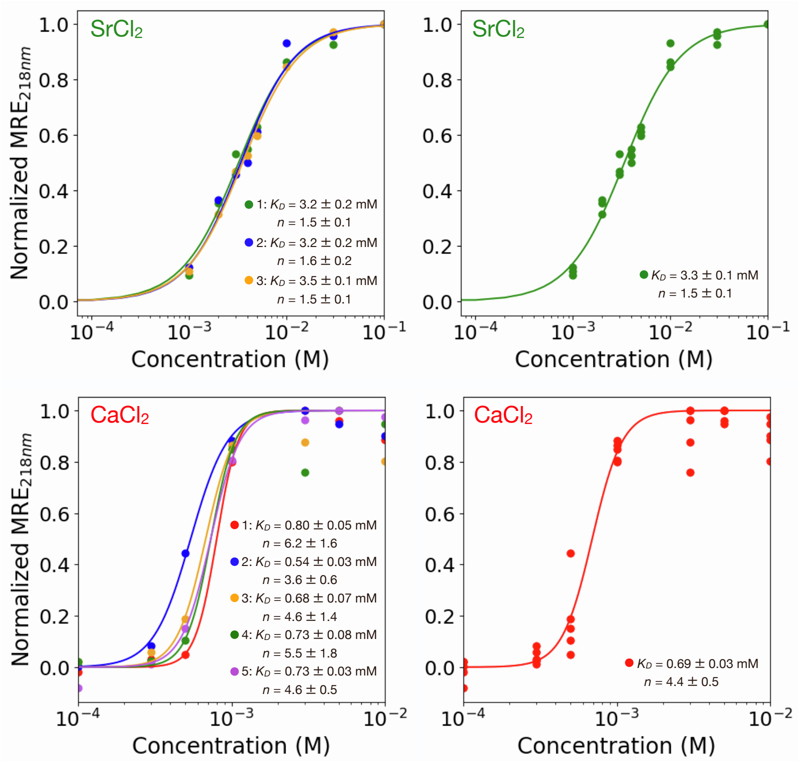

Figure S10: Hill–Langmuir fits to normalized $MRE_{218\ nm}$ upon titration with $CaCl_2$ or $SrCl_2$ (2). Fits to the Hill equation were applied to either individual replicates (left) or the combined data set (right). K_D and n reported in the main text are fit from combined data sets. The reported errors on K_D and n are the root mean square errors derived from the Hill fits to the combined data sets.

Table S5: Parameters computed from Hill fits.

**Sample**	K_D **(mM)**	n
RTX-v-Ca^{2+}	0.69 ± 0.03	4.4 ± 0.5
RTX-v-Sr^{2+}	3.3 ± 0.1	1.5 ± 0.1

4.4 CD Pro

Spectral deconvolution was performed from 200 nm to 250 nm with CDPro software using the reference set SPD48, which is the largest available reference set that includes denatured proteins (3). The results from CDSSTR, CONTIN/LL, and SELCON3 methods from each replicate were normalized and averaged to facilitate quantitative comparisons.

Table S6: Structural components of each sample, listed as a percentage of the total structure (%). Errors represent the standard deviation between replicates.

**Sample**	Helix	Sheet	Turn	Unstructured
50 mM Tris	4.3 ± 0.8	15.3 ± 1.8	10.3 ± 0.7	70.1 ± 3.1
1 mM $MgCl_2$	5.5 ± 0.1	16.3 ± 1.5	11.4 ± 1.0	66.7 ± 2.4
100 mM $MgCl_2$	5.6 ± 0.2	16.2 ± 0.6	12.1 ± 0.2	66.1 ± 1.0
1 mM $CaCl_2$	7.4 ± 0.8	25.9 ± 0.6	18.6 ± 0.3	48.2 ± 0.4
100 mM $CaCl_2$	7.7 ± 0.7	28.3 ± 0.6	20.2 ± 0.6	43.9 ± 0.3
1 mM $SrCl_2$	5.0 ± 0.6	16.3 ± 1.3	10.8 ± 0.6	67.9 ± 1.6
100 mM $SrCl_2$	7.0 ± 0.6	28.6 ± 0.2	19.9 ± 0.1	44.6 ± 0.4
1 mM $BaCl_2$	5.3 ± 0.6	15.9 ± 0.4	11.0 ± 0.3	67.8 ± 1.0
100 mM $BaCl_2$	6.1 ± 0.7	21.1 ± 3.7	16.0 ± 2.0	56.8 ± 6.3

4.5 Sample-to-sample variation

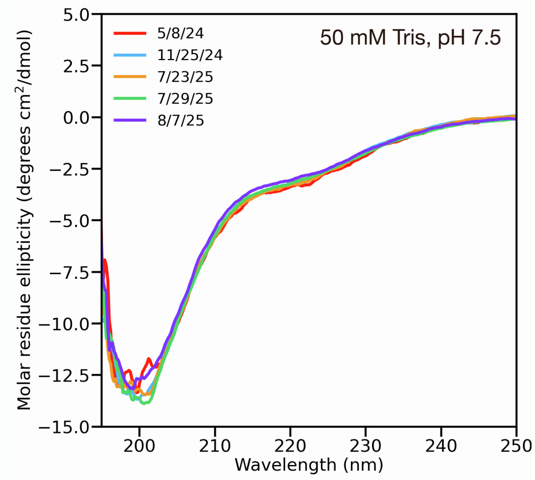

Figure S11: Sample-to-sample variation between circular dichroism experiments with RTX-v, measured in 50 mM Tris, pH 7.5. Each sample is a distinct biological replicate. Differences in noise and absolute absorbance of the spectra are attributed to aging of the UV lamp in the CD spectrometer.

Table S7: Structural components of each sample replicate in 50 mM Tris, listed as a percentage of the total structure (%). The standard deviation of the mean of all replicates is given by σ.

**Sample**	Helix	Sheet	Turn	Unstructured
5/8/24	3.6	13.2	9.4	74.0
11/25/24	3.6	13.2	9.6	74.0
7/23/25	3.8	16.7	10.8	68.8
7/29/25	5.3	15.9	10.6	68.2
8/7/25	5.3	17.6	11.2	65.9
mean ± σ	4.3 ± 0.8	15.3 ± 1.8	10.3 ± 0.7	70.1 ± 3.1

5 X-RAY CRYSTALLOGRAPHY (XRC)

5.1 Electron density maps

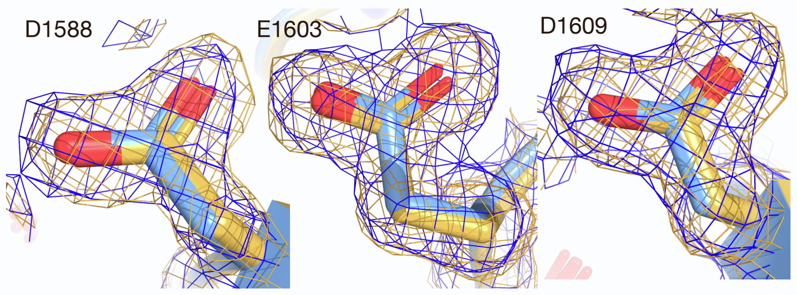

Figure S12: Overlaid 2Fo-Fc electron density maps for RTX-v-Ca^{2+} (blue) and RTX-v-Sr^{2+} (yellow) contoured at 1.8 Å from each atom. Selected residues Asp1588, Glu1603, and Asp1609 coordinate Ca^{2+}(7) or Sr^{2+}(7). Subtle shifts in side chain positions occur upon Sr^{2+} binding. Electron density maps were aligned using phenix.superpose_maps in *Phenix* (4).

5.2 Ligand–ion distances

Table S8: Distances between coordinating ligands and Ca^{2+}or Sr^{2+} in X-ray structures for RTX-v-Ca^{2+} or RTX-v-Sr^{2+}, respectively (Å). The ions are numbered in order from N(1)- to C(8)-termini. Oδ1 refers to the double-bonded oxygen atom in aspartic acid side chain carboxyls, and Oδ2 refers to the single-bonded oxygen. Oϵ1 refers to the double-bonded oxygen atom in glutamic acid side chain carboxyls, and Oϵ2 refers to the single-bonded oxygen. Each average difference was computed from the sum of all differences between Ca^{2+}–ligand and Sr^{2+}–ligand distances, divided by the number of coordinating ligands.

Ion	Ligand	Ca^{2+}	Sr^{2+}	Avg. difference
1	Asp1539O	2.4	2.4	+ 0.03 Å
	Gl1541O	2.3	2.4	
	Asn1543Oδ1	2.4	2.4	
	Gly1556O	2.3	2.4	
	Ala1558O	2.5	2.4	
	Asp1561Oδ2	2.8	2.9	
	Asp1561Oδ1	2.4	2.4	
2	Gly1557O	2.4	2.4	+ 0.03 Å
	Gly1559O	2.3	2.4	
	Asp1561Oδ2	2.3	2.3	
	Gly1574O	2.4	2.4	
	Ala1576O	2.4	2.3	
	Asp1579Oδ2	2.8	3.0	
	Asp1579Oδ1	2.5	2.5	
3	Asp1575O	2.3	2.3	- 0.02 Å
	Gly1577O	2.2	2.3	
	Asp1579Oδ2	2.4	2.3	
	Tyr1596O	2.3	2.2	
	Asp1599Oδ2	2.5	2.5	
	Asp1599Oδ1	2.3	2.3	
4	Ser1530O	2.3	2.4	+ 0.01 Å
	Arg1532O	2.3	2.3	
	Asp1534Oδ2	2.3	2.3	
	Gly1547O	2.3	2.3	
	Ala1549O	2.3	2.3	
	Asp1552Oδ2	2.8	2.9	
	Asp1552Oδ1	2.4	2.3	
5	Leu1548O	2.4	2.4	+ 0.03 Å
	Gly1550O	2.4	2.5	
	Asp1552Oδ2	2.3	2.4	
	Gly1565O	2.3	2.3	
	Glu1567O	2.3	2.3	
	Asp1570Oδ1	3.1	3.0	
	Asp1570Oδ2	2.4	2.5	
6	Asp1566O	2.3	2.5	+ 0.07 Å
	Gly1568O	2.4	2.5	
	Asp1570Oδ2	2.3	2.4	
	Gly1583O	2.4	2.5	
	Gln1585O	2.3	2.4	
	Asp1588Oδ2	3.0	2.9	
	Asp1588Oδ1	2.5	2.5	

7	Gly1584O	2.4	2.5	+ 0.1 Å
	Gly1586O	2.3	2.5	
	Asp1588Oδ2	2.3	2.5	
	Glu1603Oϵ2	2.6	2.6	
	Glu1603Oϵ1	2.5	2.6	
	Gly1605O	2.3	2.4	
	Asp1609Oδ1	2.6	2.6	
8	Gly1606O	2.3	2.3	0.00 Å
	Asp1609Oδ2	2.2	2.4	
	H2O1	2.4	2.4	
	H2O2	2.5	2.4	
	Arg1652O	2.4	2.4	
	Glu1654Oϵ2	2.5	2.4	
	Glu1654Oϵ1	2.6	2.6	

5.3 Turn-to-turn distances

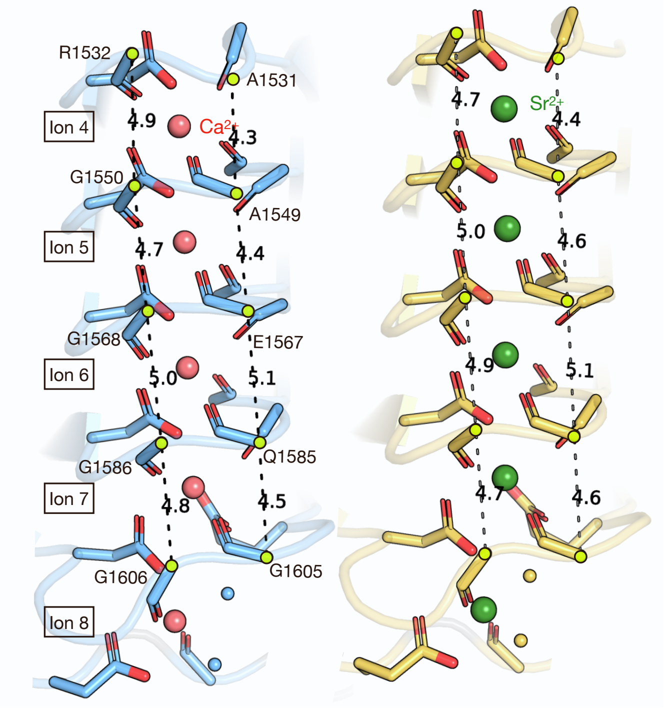

Figure S13: Side view of RTX-v-Ca^{2+} (blue) and RTX-v-Sr^{2+} (yellow), where outlined lime circles denote the α-carbon positions used to measure turn-to-turn distances. Two pairs of α-carbon atoms were selected for every turn.

Table S9: Turn-to-turn distances surrounding each ion for RTX-v-Ca^{2+} and RTX-v-Sr^{2+} (Å). Distances between two pairs of α-carbons above and below each ion were measured. Each average difference was computed from the sum of differences in α-carbon distances between the RTX-v-Ca^{2+} and RTX-v-Sr^{2+} structures divided by two.

Ion	Residues	Ca^{2+}	Sr^{2+}	Avg. difference
1	Ala1540-Ala1558	4.2	4.3	0.0 Å
	Gly1541-Gly1559	4.8	4.7	
2	Ala1558-Ala1576	4.5	4.6	+ 0.1 Å
	Gly1559-Gly1577	4.5	4.6	
3	Ala1576-Tyr1596	4.3	4.3	+ 0.05 Å
	Gly1577-Gly1597	5.3	5.4	
4	Ala1531-Ala1549	4.3	4.4	- 0.05 Å
	Arg1532-Gly1550	4.9	4.7	
5	Ala1549-Glu1567	4.4	4.6	+ 0.25 Å
	Gly1550-Gly1568	4.7	5.0	
6	Glu1567-Gln1585	5.1	5.1	- 0.05 Å
	Gly1568-Gly1586	5.0	4.9	
7	Gln1585-Gly1605	4.5	4.6	0.0 Å
	Gly1586-Gly1606	4.8	4.7	

5.4 Agreement with SAXS data

We used the FoXS software web interface to fit the theoretical scattering curves from protein crystal structures to corresponding SAXS profiles (5). We selected the 3 mM $CaCl_2$ and 10 mM $SrCl_2$ scattering profiles for comparison with the protein structure, since RTX-v is most likely to be folded in these solution conditions. For both scattering profiles, we used the RTX-v-Ca^{2+} (PDB: 9P0C) structure as the model input. RTX-v-Sr^{2+} (PDB: 9P0D) was not used as a model because the crystal structure is missing a total of 23 residues at the N- and C-termini, whereas the RTX-v-Ca^{2+} structure lacks only 7 residues. The missing residues are cloning artifacts, which are present in the protein samples used for SEC-SAXS. To achieve accurate fits with FoXS or CRYSOL, it is critical for the input protein structure or model to include all residues present in the sample measured during SAXS (6). Given the similarity between the RTX-v-Ca^{2+} and RTX-v-Sr^{2+} structures, we consider RTX-v-Ca^{2+} as an appropriate model to fit both the 3 mM $CaCl_2$ and 10 mM $SrCl_2$ scattering profiles. As a second comparison, we used AlphaFold3 (AF3) to predict a protein model from the full RTX-v sequence (7). Fits computed from both the RTX-v-Ca^{2+} structure and AF3 prediction are shown in Figure S14 for each scattering profile.

We find that fits generated from both the RTX-v-Ca^{2+} structure and AF3 prediction agree well with the scattering profiles. Notably, fits generated from AF3 predictions show better agreement with both scattering profiles (given by χ^2 values). We attribute better agreement with the AF3 prediction to its inclusion of all residues found in the SAXS protein sample.

χ^2 values for both fits to the 10 mM $SrCl_2$ scattering profile are larger than for fits to the 3 mM $CaCl_2$ profile. We attribute poorer fits to the 10 mM $SrCl_2$ data to the possible presence of multiple conformations of RTX-v. RTX-v has lower affinity to Sr^{2+} than Ca^{2+}, so it is possible that some unfolded or partially folded conformations remain at 10 mM $SrCl_2$. The presence of multiple protein conformations in solution SAXS can result in worse fits to protein models, which assume a single conformation (8).

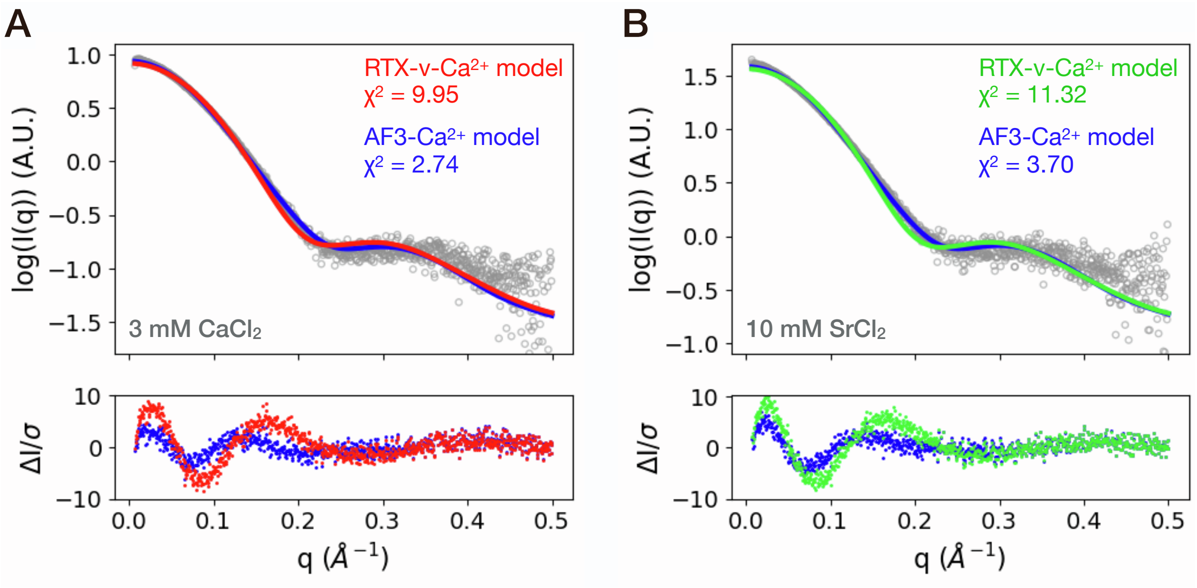

Figure S14: Theoretical scattering profiles generated from RTX-v-Ca^{2+} and the AlphaFold3 prediction agree well with experimental data collected at 3 mM $CaCl_2$ and 10 mM $SrCl_2$. Fits generated from AF3 predictions show better agreement with both experimental scattering profiles, given by χ^2 values. A) Top: fits generated from the RTX-v-Ca^{2+} structure and AF3 prediction compared to the experimental 3 mM $CaCl_2$ scattering profile. Bottom: residuals from the fits. B) Top: fits generated from the RTX-v-Ca^{2+} structure and AF3 prediction compared to the experimental 10 mM $SrCl_2$ scattering profile. χ^2 values for both fits are larger than for fits to the 3 mM $CaCl_2$ profile. We attribute the poorer fits to the 10 mM $SrCl_2$ data to the possible presence of multiple conformations of RTX-v at 10 mM $SrCl_2$.

5.5 XRC data collection and refinement

Table S10: Data collection parameters and structure refinement results for RTX-v-Ca^{2+} and RTX-v-Sr^{2+}.

**Data collection**	RTX-v-Ca^{2+}	RTX-v-Sr^{2+}
X-ray source	SSRL BL9-2	SSRL BL12-2
Wavelength (Å)	0.97946	0.76910
Space group	$P2_1$	I222
Cell dimensions		
a, b, c (Å)	37.722, 66.759, 59.072	62.420, 72.870, 73.755
α, β, γ (°)	90.00, 93.22, 90.00	90.00, 90.00, 90.00
Matthews coefficient ($Å^3$/Da)[a]	2.07	2.61
Solvent content (%)	40.5	52.8
Wilson B value ($Å^2$)	17.0	15.6
Mosaicity	0.31	0.14
Resolution (Å)[b]	37.7(1.7)	47.65(1.50)
R_{merge}[c]	0.097(0.991)	0.303(1.860)
I / σI ratio[d]	7.2(1.5)	5.0(1.2)
Completeness (%)[e]	98.4(98.2)	98.6(94.2)
Reflections (total/unique)	121,050(31,714)	354,965(26,872)
Redundancy[f]	3.8(3.8)	13.2(12.0)
**Refinement**		
Resolution (Å)	30.66-1.70	30.95-1.50
No. reflections/test set	30,046/1,581	25,532/1,314
R_{work}/R_{free}[g]	16.7/20.9	17.1/20.2
Mean B value ($Å^2$)	24.9	21.5
F_{obs}-F_{calc} correlation[h]	0.97	0.97
No. atoms		
Protein	1,281 (chain A), 1,262 (chain B)	1,151
Ligand/ion	33 (16x Ca^{2+})/(4x Zn^{2+})/(1x Cl^-)/(2x glycerol)	36 (8x Sr^{2+})/(1x Cl^-, 1x Na^+, 1x formaldehyde, 1x glycerol, 2x tris)
Water	187	111
B-factors		
Protein	22.2 (chain A)/22.0 (B)	17.4 (chain A)
Ligand/ion	17.2 (Ca^{2+} chain A)/15.7 (Ca^{2+} chain B)/21.1 (Zn^{2+}) 59.8 (Cl^-)/45.2 (glyc-erol)	12.1 (Sr^{2+}), 34.9 (Cl^-), 40.8 (Na^+), 24.1 (formaldehyde), 49.9 (glycerol), 43.3 (tris)
Water	30.4	35.4
Deviation from ideality (Rmsd values)	0.020 Å (bond length), 1.945°(bond angle)	0.022 Å (bond length), 2.424°(bond angle)
Ramachandran statistics[i]		
Most favored/allowed regions (%)	99.0 (278 over 281)	100.0 (127 over 127)
Disallowed regions (%)	1.0 (3 over 281)	0.0 (0 over 127)
PDB code	9P0C	9P0D

[a] Ratio of the volume of the asymmetric unit to the molecular weight of all protein in the asymmetric unit.
[b] Value in parentheses is for the highest-resolution shell: 1.70-1.73 Å (RTX-Ca^{2+}) and 1.50-1.58 Å (RTX-v-Sr^{2+}).
[c] Reliability factor for symmetry-related reflections calculated as: $R_{merge} = \Sigma_{hkl}\Sigma$j=1 to N | I_{hkl} – I_{hkl} (j) | / Σ_{hkl} Σj=1 to N I_{hkl} (j), where N is the redundancy of the data. The cumulative value at the highest-resolution shell is in parentheses.
[d] Ratio of mean intensity to the mean standard deviation of the intensity over the entire resolution range.
[e] Fraction of measured reflections to possible observations at the resolution range.
[f] Number of measurements of individual, symmetry unique reflections.
[g] Average deviation between the observed and calculated structure factors calculated as: $R_{work} = \Sigma_{hkl}||F_{obs}| - |F_{calc}|| / \Sigma_{hkl}|F_{obs}|$, where the F_{obs} and F_{calc} are the observed and calculated structure factor amplitudes of reflection hkl. R_{free} is equal to R_{factor} but for a randomly selected 5.0 % subset of the total reflections that were held aside throughout refinement for cross-validation.
[h] Correlation coefficient between observed and calculated structure factor amplitudes.
[i] According to Procheck for non-glycine and non-proline residues.

5.6 AlphaFold3 predictions

To investigate whether AlphaFold3 (AF3) can resolve ion-dependent RTX-v conformations, we predicted RTX-v structures in the presence of different ions (7). AF3 was developed to predict biomolecular interactions, including ionic interactions. Currently, the only ion ligands supported in AF3 are Mg^{2+}, Zn^{2+}, Cl^{-}, Ca^{2+}, Na^{+}, Mn^{2+}, K^{+}, Fe^{3+}, Cu^{2+}, and Co^{2+}. We tested AF3 predictions of RTX-v structure under the conditions depicted in Figure S15. We compared conditions with either 2 or 8 ion ligands to test whether AF3 can predict partially folded structures. Additionally, we included Mn^{2+} and Co^{2+} to probe whether AF3 may predict alternative RTX-v structures. Our results demonstrate that based on available crystallography data of RTX-v, AF3 is unable to predict disordered or partially folded states. All ionic conditions, including the ion-free condition, produced similar structure predictions with high pLDDT scores that closely resemble the RTX-v-Ca^{2+} structure. The predicted local distance difference test (pLDDT) score indicates local confidence in the predicted structure.

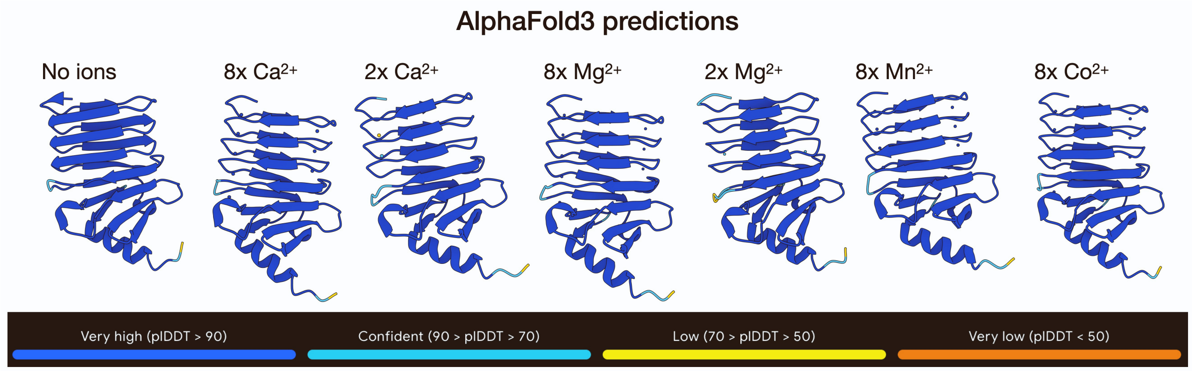

Figure S15: AlphaFold3 predictions of RTX-v structures in the presence of different ions. Under all conditions, AF3 predict similar structures with high pLDDT sctores that closely resemble the RTX-v-Ca^{2+} structure.

REFERENCES

1. Grant, T. D., 2018. Ab initio electron density determination directly from solution scattering data. *Nature Methods* 15:191–193. DOI: 10.1038/nmeth.4581.

2. Gesztelyi, R., J. Zsuga, A. Kemeny-Beke, B. Varga, B. Juhasz, and A. Tosaki, 2012. The Hill equation and the origin of quantitative pharmacology. *Archive for History of Exact Sciences* 66:427–438. DOI: 10.1007/s00407-012-0098-5.

3. Sreerama, N., and R. W. Woody, 2000. Estimation of Protein Secondary Structure from Circular Dichroism Spectra: Comparison of CONTIN, SELCON, and CDSSTR Methods with an Expanded Reference Set. *Analytical Biochemistry* 287:252–260. DOI: 10.1006/abio.2000.4880.

4. Liebschner, D., P. V. Afonine, M. L. Baker, G. Bunkóczi, V. B. Chen, T. I. Croll, B. Hintze, L.-W. Hung, S. Jain, A. J. McCoy, N. W. Moriarty, R. D. Oeffner, B. K. Poon, M. G. Prisant, R. J. Read, J. S. Richardson, D. C. Richardson, M. D. Sammito, O. V. Sobolev, D. H. Stockwell, T. C. Terwilliger, A. G. Urzhumtsev, L. L. Videau, C. J. Williams, and P. D. Adams, 2019. Macromolecular structure determination using X-rays, neutrons and electrons: recent developments in *Phenix*. *Acta Crystallographica Section D* 75:861–877. DOI: 10.1107/S2059798319011471.

5. Schneidman-Duhovny, D., M. Hammel, and A. Sali, 2010. FoXS: a web server for rapid computation and fitting of SAXS profiles. *Nucleic Acids Research* 38:W540–W544. DOI: 10.1093/nar/gkq461.

6. Vestergaard, B., S. Sanyal, M. Roessle, L. Mora, R. H. Buckingham, J. S. Kastrup, M. Gajhede, D. I. Svergun, and M. Ehrenberg, 2005. The SAXS Solution Structure of RF1 Differs from Its Crystal Structure and Is Similar to Its Ribosome Bound Cryo-EM Structure. *Molecular Cell* 20:929–938. DOI: 10.1016/j.molcel.2005.11.022.

7. Abramson, J., J. Adler, J. Dunger, R. Evans, T. Green, A. Pritzel, O. Ronneberger, L. Willmore, A. J. Ballard, J. Bambrick, S. W. Bodenstein, D. A. Evans, C.-C. Hung, M. O'Neill, D. Reiman, K. Tunyasuvunakool, Z. Wu, A. Žemgulytė, E. Arvaniti, C. Beattie, O. Bertolli, A. Bridgland, A. Cherepanov, M. Congreve, A. I. Cowen-Rivers, A. Cowie, M. Figurnov, F. B. Fuchs, H. Gladman, R. Jain, Y. A. Khan, C. M. R. Low, K. Perlin, A. Potapenko, P. Savy, S. Singh, A. Stecula, A. Thillaisundaram,

C. Tong, S. Yakneen, E. D. Zhong, M. Zielinski, A. Žídek, V. Bapst, P. Kohli, M. Jaderberg, D. Hassabis, and J. M. Jumper, 2024. Accurate structure prediction of biomolecular interactions with AlphaFold 3. *Nature* 630:493–500. DOI: 10.1038/s41586-024-07487-w.

8. Round, A., E. Brown, R. Marcellin, U. Kapp, C. S. Westfall, J. M. Jez, and C. Zubieta, 2013. Determination of the GH3.12 protein conformation through HPLC-integrated SAXS measurements combined with X-ray crystallography. *Acta Crystallographica Section D* 69:2072–2080. DOI: 10.1107/S0907444913019276.
