# Supplementary material for: Ion-selective conformational stabilization of a disordered repeats-in-toxin protein domain
Source: Biophys J. 2025 Oct 13;124(23):4243–54. doi: 10.1016/j.bpj.2025.10.014 (PMC12821022; doi:10.1016/j.bpj.2025.10.014)
Supplement: Document S2. Article plus supporting material [file mmc3.pdf]

Biophysical Journal
Article

Biophysical Society

Ion-selective conformational stabilization of a disordered repeats-in-toxin protein domain

Alana P. Gudinas,[1] Gatha M. Shambharkar,[2] Marina P. Chang,[2] Daniel Fernández,[3] Tsutomu Matsui,[4] and Danielle J. Mai[2,5,*]

[1]Department of Physics, Stanford University, Stanford, California; [2]Department of Materials Science & Engineering, Stanford University, Stanford, California; [3]Macromolecular Structure Group, Nucleus at Sarafan ChEM-H, Stanford University, Stanford, California; [4]Stanford Synchrotron Radiation Lightsource, SLAC National Accelerator Laboratory, Menlo Park, California; and [5]Department of Chemical Engineering, Stanford University, Stanford, California

ABSTRACT Ion-binding intrinsically disordered proteins (IDPs) recruit and bind to specific metal ions to perform critical biological functions. In proteins where ion binding and structural transitions are coupled, interactions with off-target toxic metals can dramatically disrupt protein structure and function, exemplified by lead and mercury poisoning. Understanding the complex mechanisms underlying how IDPs exclude or allow binding to different ionic species is crucial for addressing the origins of metal toxicity in biological systems. Here, we elucidate mechanisms of ion selectivity in an IDP that adopts a structure upon Ca^{2+} binding. We probed ion-induced conformational changes of a repeats-in-toxin (RTX) protein domain in the presence of different ion ligands—Mg^{2+}, Ca^{2+}, Sr^{2+}, and Ba^{2+}—with chemical similarities but drastically different ionic radii. RTX adopts ion-selective conformations measured by x-ray crystallography, small-angle x-ray scattering, and circular dichroism. High-resolution x-ray structures reveal that Sr^{2+} induces a nearly identical RTX structure as natively binding Ca^{2+}, enabled by the intrinsic flexibility and disorder of the protein. Small-angle x-ray scattering and circular dichroism indicate that smaller Mg^{2+} does not induce a significant conformational change in RTX, whereas larger Ba^{2+} induces a partially folded structure. These results highlight the importance of geometric constraints imposed by protein structure in determining metal ion selectivity, yielding insights into how off-target ion binding may result in protein misfolding and malfunction.

SIGNIFICANCE The mechanisms of metal selectivity in ion-binding proteins are complex, which is exacerbated in the case of conformationally flexible, intrinsically disordered proteins. In this study, we elucidate the roles of ion size and protein binding site geometry in the ion selectivity of a calcium-ion-binding intrinsically disordered protein. Ion-induced structural transitions are probed using multiscale structural experiments to find that protein flexibility and disorder allow promiscuous binding to strontium ions, whereas the geometric constraints of binding sites prevent full structural formation with smaller magnesium ions or larger barium ions. Importantly, barium stabilizes partially folded structures, uncovering how off-target metal binding may induce protein misfolding and downstream biological malfunction.

INTRODUCTION

Intrinsically disordered proteins (IDPs) adopt dynamic and heterogeneous conformations to perform critical biological functions (1–3). Many IDPs undergo drastic conformational changes upon binding to molecular and ionic ligands, sometimes transitioning from disordered states to folded structures (4,5). Proteins that fold upon binding to metal ions include charged sequence motifs, which have evolved selectivity for specific ionic species (6,7). In these cases, interactions with off-target ions can dramatically disrupt protein structure and function, leading to downstream biological malfunction (8). Well-studied examples include cadmium-induced protein aggregation (9), lead competition for calcium-binding sites (10), and mercury binding to cysteine residues (11). In IDPs where folding and ligand binding are coupled, off-target ion binding can promote aggregation linked to neurodegenerative disorders (12–14). The mechanisms underlying how IDPs exclude or allow binding to different ionic species are complex. These complexities are exacerbated when IDPs

Submitted June 6, 2025, and accepted for publication October 8, 2025.
*Correspondence: djmai@stanford.edu
Editor: Scott Showalter.
https://doi.org/10.1016/j.bpj.2025.10.014
© 2025 The Author(s). Published by Elsevier Inc. on behalf of Biophysical Society.
This is an open access article under the CC BY license (http://creativecommons.org/licenses/by/4.0/).

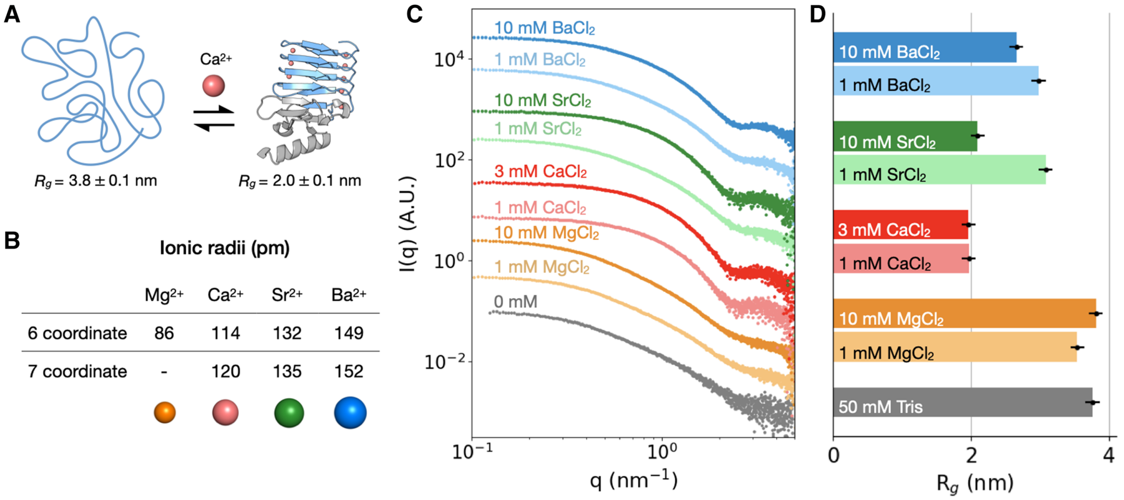

FIGURE 1 SAXS quantifies conformational changes in RTX-v upon interacting with group II ions. (*A*) RTX-v transitions from an intrinsically disordered conformation to a folded structure comprising repeats of ion-binding turns and hydrophobic β sheets. The Ca^{2+}-driven conformational change is marked by a decrease in radius of gyration (R_g). (*B*) Six- and seven-coordinate ionic radii for group II ions Mg^{2+}, Ca^{2+}, Sr^{2+}, and Ba^{2+} (19). Ions were selected for similar chemical characteristics as Ca^{2+} and differing ionic radii. (*C*) SAXS profiles ($I(q)$ vs. q) of RTX-v incubated with 1–10 mM group II metal chlorides, vertically offset for clarity. (*D*) R_g computed from Guinier analysis of SAXS scattering profiles. Error bars represent the order of magnitude of fitting errors, which range from 0.01 nm to 0.03 nm. Ca^{2+}, Sr^{2+}, and Ba^{2+} induced compaction of the protein compared with the divalent cation-free condition, whereas incubation with Mg^{2+} did not reduce RTX-v R_g. For a Figure360 author presentation of Figure 1, see https://doi.org/10.1016/j.bpj.2025.10.014#mmc2.

undergo structural transitions upon binding (6,15,16). To understand the coupling between ion-dependent folding and intrinsic ion selectivity, it is critical to investigate ion-induced conformational changes in IDPs.

A family of disordered proteins that undergo exemplary ion-induced conformational changes are repeats-in-toxin (RTX) proteins, which fold upon binding to calcium ions (Fig. 1 *A*) (17,18). RTX proteins are disordered in calcium-poor bacterial intracellular environments (100 nM Ca^{2+}) and undergo calcium-dependent folding upon translocation and secretion to calcium-rich extracellular environments (10 μM to over 10 mM Ca^{2+}) (20). A well-studied RTX protein is adenylate cyclase toxin ($CyaA^{1\text{-}1706}$) from *Bordetella pertussis*, which contains 40 repeats of a Ca^{2+}-binding motif described by the amino acid sequence GGXGXDXUX. In this motif, X can be any amino acid, and U is an aliphatic amino acid (21). Glycine residues confer flexibility to the protein, and aspartic acid provides negative charge to recruit cations. The repeat region forms five distinct blocks denoted with Roman numerals i to v proceeding from N- to C-terminal domains, respectively. The fifth block—denoted RTX-v ($CyaA^{1529\text{-}1680}$)—binds most strongly to Ca^{2+} and consists of nine repeats of the consensus sequence flanked by a C-terminal capping domain. The C-terminal RTX-v binds to eight Ca^{2+} ions and initiates folding as the protein is secreted through the type I secretion system of Gram-negative bacteria. Cooperative Ca^{2+}-induced folding of the entire RTX protein then proceeds successively from the C-terminus to the N-terminus (22–24).

The coordination environment of RTX-v is most flexible at the solvent-exposed C-terminal Ca^{2+} at position 8—denoted Ca^{2+}[8]—where two water molecules assist in Ca^{2+} coordination. The remaining seven Ca^{2+} ions are periodically coordinated by backbone carbonyls and side-chain carboxyls within the ion-binding turns (GGXGXD) in a pentagonal bipyramidal geometry. Successive binding of each ion triggers partial folding of the structure and increases the Ca^{2+} affinity of the remaining sites. Ca^{2+}-induced folding of the disordered protein forces hydrophobic residues (XUX) to form a β-roll structure. Without Ca^{2+}, electrostatic repulsion between aspartic acid carboxylate groups drives the intrinsic disorder of RTX-v (25).

Off-target binding of CyaA to ions beyond Ca^{2+} prevents formation of the full β-roll structure and disrupts CyaA activity (26). RTX-v binds to Ca^{2+} with micromolar to millimolar affinity, showing selectivity over high background concentrations of Mg^{2+} (100 mM); however, Ca^{2+} can be replaced by other ions (26–29). In CyaA, small fractions of Ca^{2+}-binding sites can be replaced with barium and cadmium, which hinders toxin activity (30).

Recently, the distinct ion-dependent conformational changes of RTX-v have been used as a proxy to measure its affinity to ions beyond Ca^{2+}. Förster resonance energy transfer measurements suggest that RTX-v binds to Sr^{2+} with 10-fold weaker affinity than Ca^{2+}. Interestingly, trivalent cations including rare earth elements induce a different compact structure entirely (27,31,32). It is clear RTX-v may bind to ions beyond Ca^{2+}, but its ion-dependent conformational states remain poorly characterized.

We elucidate mechanisms of ion selectivity in RTX-v by probing ion-induced conformational changes in the presence of different ion ligands. We combined small-angle x-ray

scattering, x-ray crystallography, and circular dichroism to study the structure of RTX-v in the presence of group II divalent cations. Mg^{2+}, Ca^{2+}, Sr^{2+}, and Ba^{2+} were chosen for their chemical similarities but drastically different ionic radii (Fig. 1 *B*). We found that Sr^{2+} successfully replaces all Ca^{2+}-binding sites in RTX-v, stabilizing a nearly identical β-roll structure. Smaller Mg^{2+} does not induce a conformational change in RTX-v, whereas larger Ba^{2+} appears to promote partially folded structures. The disorder and flexibility of RTX-v give rise to promiscuous ion binding, but its repetitive sequence restricts cooperative binding to geometrically favorable ions (33).

MATERIALS AND METHODS

All materials are listed in Table S1.

Protein preparation

RTX-v was produced using directional cloning and recombinant protein expression (28). A gene fragment encoding RTX-v was designed with an N-terminal 6×His tag to aid purification. The fragment was subcloned using BamHI and HindIII restriction sites into expression vector pQE-9. DNA and amino acid sequences are provided in the supporting material. The plasmid is available for use from Addgene (catalog #225964). Recombinant RTX-v was expressed in T7 Express lysY/I^q *Escherichia coli*. Briefly, 10 mL freshly grown overnight culture was inoculated into 1 L LB media supplemented with 10 mg L^{-1} ampicillin. Cultures were incubated at 37°C and 220 rpm. Once the optical density at 600 nm reached 0.8–1.0 (2–3 h), cultures were induced with 1 mM isopropyl-β-D-1-thiogalactopyranoside. Expression proceeded for 6 h at 37°C and 220 rpm. Cells were harvested by centrifugation at 4136 × *g* for 10 min. Pelleted cells were resuspended in 25 mL denaturing lysis buffer (8 M urea, 1.0 M NaCl, 100 mM sodium phosphate, and 10 mM Tris (pH 8.0)) and stored at −70°C.

Expressed RTX-v was recovered from cell pellets and isolated using immobilized metal affinity chromatography to capture 6×His-tagged proteins, dialysis to remove excess ions, and lyophilization to remove water. Cell pellets were defrosted with an additional 25 mL of denaturing lysis buffer and lysed by sonication. Crude lysates were clarified by centrifugation (9250 rpm for 1 h) and filtration (0.45 μm). Clarified lysates were incubated with NiNTA resin for at least 2 h at ambient temperature or overnight at 4°C. Protein-bound resins were washed with denaturing lysis buffer supplemented with 10 mM imidazole, followed by elution with 250 mM imidazole. Protein purity was assessed by sodium dodecyl sulfate polyacrylamide gel electrophoresis (Fig. S1). Pure fractions were dialyzed against chelating buffer (1 mM EGTA, 50 mM NaCl, and 10 mM Tris (pH 8.0), three exchanges) and ultrapure water (seven exchanges). Water was removed by lyophilization, and purified proteins were stored at −20°C. Protein expression yields ranged from 100 mg to 125 mg per 1 L culture.

Small-angle x-ray scattering data collection and analysis

Ion-dependent conformations of RTX-v were obtained using size-exclusion chromatography coupled to small-angle x-ray scattering (SEC-SAXS, SASBDB: SASDXM8, SASDXN8, SASDXP8, SASDXQ8, SASDXR8, SASDXS8, SASDXT8, SASDXU8, SASDXV8, SASDXW8, SASDXX8, SASDXY8, and SASDXZ8). SEC-SAXS was conducted at the Stanford Synchrotron Radiation Lightsource (SSRL, Menlo Park, CA), beamline 4-2, with a 1.7-m sample-to-detector distance and 11-keV beam energy (wavelength $\lambda = 1.127$ Å) (34). Lyophilized protein was resuspended at 10 mg mL^{-1} in running buffer (5 mM dithiothreitol and 50 mM Tris (pH 7.5)) supplemented with up to 10 mM $MgCl_2$, $CaCl_2$, $SrCl_2$, $BaCl_2$, or KCl. Dithiothreitol protects protein samples from radiation damage (35). Samples were dissolved overnight at 4°C and filtered (0.22 μm) before measuring protein concentration (absorbance at 280 nm, molar extinction coefficient 18,450 M^{-1} cm^{-1}). Filtered protein solutions are stable for at least 4 days.

SAXS data were collected downstream of an in-line SEC, outfitted with a Superdex 200 column equilibrated with running buffer supplemented with metal chlorides. Using a 50 μL sample injection volume (30 μL for 10 mM KCl and 3 mM $CaCl_2$) and a 0.05 mL/min flow rate, scattering images were collected with a 2-s exposure every 5 s for each metal chloride condition. Data reduction and initial analyses were performed using the BL4-2 automated SEC-SAXS data processing and analysis pipeline, *SECPipe* (https://www-ssrl.slac.stanford.edu/smb-saxs/node/1860), which implements the program *SASTOOL* (https://www-ssrl.slac.stanford.edu/smb-saxs/node/1914) and ATSAS AUTORG (36). Briefly, 50 image frames in the first 100 frames were scaled and averaged to create a buffer scattering profile, which was subtracted from each subsequent profile. Scattering images were reduced to one dimension and presented as scattered intensity I as a function of scattering vector $q = 4\pi\sin(\theta)/\lambda$, where 2θ is the scattering angle, and λ is the x-ray wavelength. Guinier analysis using AUTORG was conducted to approximate the radius of gyration, R_g, in each scattering profile. R_g is approximated as follows:

$$I(q) \approx I(0) e^{-q^2 R_g^2 / 3},$$

where $I(0)$ is the intensity at $q = 0$. R_g and $I(0)$ were extracted from linear fits to the Guinier plots limited to $qR_g < 1.3$ (37). Rg are reported as the fit values and order of magnitude of fitting errors, which range from 0.01 nm to 0.03 nm. For each SEC-SAXS elution profile, an average of five scattering profiles where R_g was constant were selected for further analysis. Visual inspection of the selected curves was performed using ATSAS PRIMUS (38). Automatically computed *I(q)* scale factors from PRIMUS were applied in all scattering profiles shown in this work. To independently compute R_g, pairwise distance distributions $P(r)$ were generated in ATSAS GNOM (Fig. S6) (39). The D_{max} value that ensured $P(D_{max}) = 0$ and maximized the GNOM total quality estimate was selected. The data collection and structural parameters are summarized in Tables S3 and S4, respectively.

To visualize solution conformations of RTX-v, 3D electron density maps were reconstructed using the Density from Solution Scattering (DENSS) algorithm (40). Twenty electron density maps were generated from each SAXS profile before alignment with the Ca^{2+}-bound RTX-v structure. Maps were visualized as transparent surfaces in PyMOL, colored from lowest (blue, 2σ) to highest (red, 15σ) electron densities (Figs. S4 and S5) (40).

Protein crystallization

To resolve ion-bound protein structures, RTX-v was incubated with Mg^{2+}, Ca^{2+}, Sr^{2+}, and Ba^{2+} during protein crystallization. Fresh protein solutions were prepared at 10 mg mL^{-1} in tris-buffered saline (TBS, 50 mM Tris, and 150 mM NaCl (pH 8)) and equilibrated overnight at 4°C. Two milliliters of protein solution was loaded onto a Superdex 75 size-exclusion chromatography column and fractionated using an Äkta pure chromatography system. Fraction purity was analyzed using sodium dodecyl sulfate polyacrylamide gel electrophoresis. Pure fractions were concentrated using Amicon spin columns (3 kDa MWCO) before crystallization screens.

Protein crystallization conditions were screened in sitting drop vapor diffusion plates. Protein solutions were supplemented with 30 mM $MgCl_2$, 10 mM $CaCl_2$, 10 mM $SrCl_2$, or 30 mM $BaCl_2$. Some precipitate was observed upon supplementing with $CaCl_2$; in this case, the precipitate was removed by centrifugation, and the protein concentration in the supernatant remained unchanged. Ion-supplemented protein solutions were mixed 1:1 with crystallization screening formulations using a Douglas Oryx8 Crystallization Robot for automated screen setup. All protein crystallization screens were incubated at 16°C. RTX-v with 10 mM $CaCl_2$ was incubated with Morpheus I and II and JCSG core suite I-IV screens.

RTX-v with 10 mM $SrCl_2$ was incubated with Morpheus I and II, JCSG core suites I and II, MCSG-4, and Shotgun I screens. Conditions with 30 mM $MgCl_2$ or $BaCl_2$ were incubated with MCSG-4, Memgold, Morpheus I and II, and Shotgun I screens. For $MgCl_2$ and $BaCl_2$ screens, incubation for over 3 months did not produce diffraction-quality crystals comprising both protein and target cations. Crystal formation was monitored using a Formulatrix RockImager2 UV-Vis Imager. For screens containing 10 mM $CaCl_2$ or 10 mM $SrCl_2$, approximately 10 crystallization conditions produced crystals. In general, crystals harvested from different crystallization conditions showed variation in x-ray diffracting power, and therefore, several were screened for initial data quality assessment. The best candidates were selected for high-resolution data collection.

RTX-v-Ca^{2+}

Diffraction-quality crystals were grown from a 1:1 mixture of 7.5 mg/mL protein solution (50 mM Tris, 150 mM NaCl, and 10 mM $CaCl_2$ (pH 8)) and reservoir solution (Morpheus II H12: 40 mM polyamines, 100 mM glycylglycine, and 2-amino-2-methyl-1,3-propanediol buffer, 31% v/v: 10% w/v PEG 20k; 50% w/v trimethylpropane, 2% w/v NDSB-195 (pH 8.5)). Crystals were harvested 2.5 months after setting up screening plates and immediately frozen in liquid N_2 in a 3:1 mixture of the Morpheus II H12 reservoir solution and glycerol.

RTX-v-Sr^{2+}

Diffraction-quality crystals were grown from a 1:1 mixture of 6.9 mg/mL protein solution (50 mM Tris, 150 mM NaCl, 10 mM $SrCl_2$ (pH 8)) and reservoir solution (Morpheus I H4: 0.1 M amino acids, 100 mM imidazole and 2-(N-morpholino)ethanesulfonic acid monohydrate buffer, 37.5% v/v: 25% v/v 2-methyl-2,4-pentanediol; 25% w/v PEG 1000, 25% w/v PEG 3350 (pH 6.5)). Crystals were harvested 1.5 months after setting up the screens and immediately frozen in liquid N_2 in a 3:1 mixture of the Morpheus I H4 reservoir solution and glycerol.

X-ray data collection, structure solution, and refinement

Data for single Ca^{2+}-bound and Sr^{2+}-bound protein crystals were collected at SSRL beamlines 9-2 and 12-2, respectively (41). Data collection parameters and structure refinement results are detailed in Table S10.

RTX-v-Ca^{2+}

High-resolution x-ray data were collected for the Ca^{2+}-bound RTX-v crystal to a Bragg spacing of 1.70 Å (PDB: 9P0C). Data were integrated using *XDS* (42) and scaled with AIMLESS (43). The crystal belonged to the monoclinic space group $P2_1$ and contained two polypeptide chains per asymmetry unit. The structure was solved by the molecular replacement method with Phaser (44), using the Block V RTX domain of the adenylate cyclase toxin from *Bordetella pertussis* (PDB: 5CXL (18)). The search model included the polypeptide chain stripped of nonprotein atoms. Residues 1513–1684 (chain A) and 1514–1682 (chain B) were unambiguously traced in the electron density maps. Extra electron density was attributed to residues in the N-terminal 6×His tag, which coordinated zinc ions. Additional peaks in electron density were detected and assigned to eight bound Ca^{2+} ions. Throughout refinement with REFMAC5 (45), manual adjustments of the polypeptide chain were made in COOT (46). Solvent water molecules, glycerol, and a chloride ion were assigned. Water molecules were placed based on their hydrogen bonding properties. Refinement continued until convergence of R_{work} and R_{free} and reached an agreement between the model and experimental data. Throughout this manuscript, the chain A polypeptide is referenced as RTX-v-Ca^{2+}.

RTX-v-Sr^{2+}

High-resolution x-ray data were collected for the Sr^{2+}-bound RTX-v crystal to a Bragg spacing of 1.50 Å using a 15 × 15 μm microfocused beam (PDB: 9P0D). Data were integrated using DIALS (47) and scaled with SCALA (48). The presence of strontium in the sample was detected by employing an excitation scan to measure the fluorescence counts from any element present in the sample with an excitation energy of 16.605 KeV. A fluorescence peak at 14.100 keV was detected, confirming strontium in the sample. To use this signal to solve the structure via experimental phasing, a multiwavelength anomalous diffraction (MAD) scan was performed to define the energies for MAD data collection at the strontium peak. Energy values using calculated anomalous scattering factors from the fluorescence data were selected with the program *autochooch* implemented in Web-Ice (49). The f" and f' values were obtained as well as the suggested peak (maximum f"), inflection (minimum f'), and remote (high f" and f') energies for MAD data collection. MAD data were collected at energies 16.120 (peak), 16.112 (inflection), and 9.000 (remote) KeV. The anomalous signal was significant at 2.27 Å. Data at the strontium peak were sufficient for structure solution. The CRANK2 (50) automated pipeline (via programs SFtools, PEAKMAX, SHELXC/D/E (51), REFMAC5 (45), MAPROT (52), Solomon (53), Multicomb, Parrot (46), and Buccaneer (54)) within the CCP4 (55) suite using combined iterative model building with density modification with phased refinement led to an almost completely built model (152 residues out of 156; R-factor/R_{free}: 0.36/0.39). The model was completed by manual adjustments of the polypeptide chain in COOT (46). A REFMAC-SAD substructure refinement was used to determine the occupancy of the strontium ions. In the final stages of refinement, their occupancies were fixed (the occupancies range from 0.65 to 1.00). The crystal belonged to the orthorhombic space group I222 and contained one polypeptide chain per asymmetry unit. The single polypeptide chain encompasses residues Leu1525–Asp1680. Eight copies of the strontium ion are bound to the structure making 45 contacts with protein backbone and side-chain atoms. Further ligands include tris and glycerol possibly arising from the buffer condition and cryoprotectant solution. Ions chloride and sodium and a tentatively assigned formaldehyde molecule (possibly a PEG impurity (56)) were placed at occupancy = 0.50 in special positions.

Circular dichroism spectroscopy

Ion-dependent protein structural changes were monitored using CD spectroscopy (PCDDB: deposition in progress). Lyophilized protein was resuspended in 50 mM Tris (pH 7.5), equilibrated overnight at 4°C, filtered (0.2 μm polyethersulfone membrane), and measured using UV-vis to determine protein concentration. Protein solutions were supplemented with up to 100 mM metal chlorides, and final protein concentrations were between 10 and 13 μM. CD experiments were conducted on at least three biological replicates using a Jasco J-815 spectropolarimeter (Fig. S8). Samples were loaded into a 1-mm pathlength cuvette (Hellma) and held at 20°C. Scans were performed from 250 nm to 190 nm with 0.2-nm steps, 1-nm bandwidth, and 2-s integration times, at a scanning speed of 50 nm/min. Spectra were averaged between 10 scans, and all spectra were corrected by background subtraction of the protein-free buffer (0–100 mM metal chloride, 50 mM Tris (pH 7.5)).

Spectral deconvolution was performed from 200 nm to 250 nm with CDPro software using the reference set SPD48, which is the largest available reference set that includes denatured proteins (57). The results from CDSSTR, CONTIN/LL, and SELCON3 methods were normalized and averaged to facilitate quantitative comparisons.

RESULTS

Group II ions induce nonmonotonic changes in RTX-v size and flexibility

We observe distinct behaviors in 1D scattering profiles of unfolded and folded proteins (Fig. 1 *C*, page 2). For unfolded proteins in divalent cation-free buffer, the

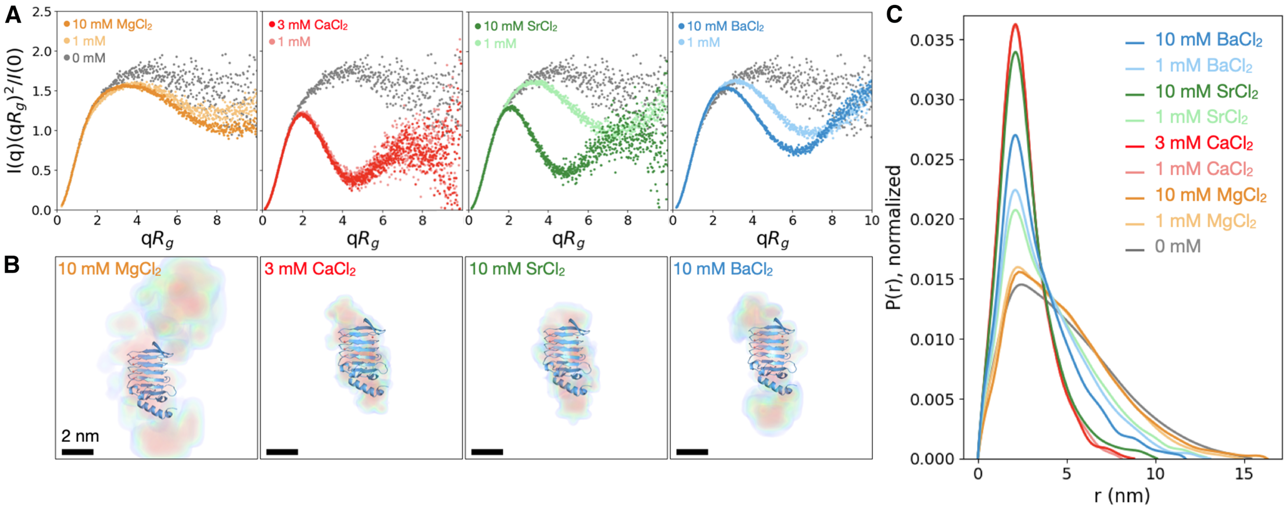

FIGURE 2 Diverse conformational states of RTX-v measured by SAXS. (*A*) Dimensionless Kratky plots for SAXS profiles shown in Fig. 1 reveal enhanced globular character in RTX-v with Ca^{2+}, Sr^{2+}, and Ba^{2+}. (*B*) DENSS projections of electron density from SAXS profiles overlaid with the Ca^{2+}-bound RTX-v crystal structure for scale. Electron density maps are colored from lowest (*blue*, 2σ) to highest (*red*, 15σ) electron densities, where σ represents the standard deviation of the mean electron density (40). (*C*) $P(r)$ distributions for each SAXS profile.

scattering intensity decreases as a function of q and lacks distinct features. Similar scattering profiles are observed with up to 10 mM $MgCl_2$. In comparison, the full-length CyaA[1-1706] does not fold in response to Mg^{2+}, though Mg^{2+} can occupy its ion-binding sites (30). For folded proteins in the presence of 1 mM and 3 mM $CaCl_2$ (henceforth referred to as RTX-v-Ca^{2+}), the scattering intensity increases in the mid-q region between 0.3 nm^{-1} and 2 nm^{-1} (Fig. 1 *C*). Emergent features in the high-q region are attributed to enhanced structural order within the folded protein (58,59).

Upon incubation with Sr^{2+} or Ba^{2+}, RTX-v adopts intermediate conformations between those of the cation-free unfolded protein and Ca^{2+}-bound folded protein. With 1 mM $SrCl_2$ or 1 mM $BaCl_2$, the scattered intensities resemble the cation-free sample, with slight enhancement of the high-q scattering intensity characteristic of structure formation. With 10 mM $SrCl_2$, the structural features of the folded protein are fully recovered, indicated by enhanced mid-q scattering and high-q features. This folded protein state is hereafter referred to as RTX-v-Sr^{2+}. With 10 mM $BaCl_2$, similar features appear in the high-q region. However, the mid-q scattering intensity with $BaCl_2$ is weaker than with $CaCl_2$ and $SrCl_2$. We attribute suppressed mid-q scattering and a high-q peak to partial folding in the presence of sufficient Ba^{2+} (60).

To quantify ion-dependent RTX-v sizes, we analyzed the low q regions using the Guinier approximation to compute R_g (Fig. S3). R_g ranged from 3.8 $\pm$ 0.1 nm for unfolded RTX-v in 50 mM Tris to 2.0 $\pm$ 0.1 nm for RTX-v-Ca^{2+} (Fig. 1 *D*, page 2). With 1 mM and 10 mM $MgCl_2$, respective radii of 3.5 $\pm$ 0.1 nm and 3.8 $\pm$ 0.1 nm are consistent with the cation-free R_g, indicating that RTX-v remains disordered in the presence of Mg^{2+}. With 1 mM $SrCl_2$, R_g of 3.1 $\pm$ 0.1 nm indicates partial folding of RTX-v. With 10 mM $SrCl_2$, RTX-v adopts a conformation with R_g of 2.1 $\pm$ 0.1 nm, resembling the R_g of RTX-v-Ca^{2+}. This resemblance reflects the similar scattering profiles between RTX-v-Ca^{2+} and RTX-v-Sr^{2+}. With 1 mM $BaCl_2$ and 10 mM $BaCl_2$, respective radii of 3.0 $\pm$ 0.1 nm and 2.7 $\pm$ 0.1 nm indicate a distinct Ba^{2+}-induced protein conformation. Intermediate R_g suggests that $BaCl_2$ contributes to the compaction of RTX-v, but not to the extent of folded RTX-v-Ca^{2+}. Inspection of the Guinier plot with 1 mM $BaCl_2$ revealed aggregation of RTX-v, indicated by an upturn at low q (60). RTX-v aggregates in the presence of Ca^{2+} (61,62) and may be more prone to aggregation in a partially folded state. To further resolve intermediate RTX-v states, additional scattering profiles were collected at 2 mM $SrCl_2$, 3 mM $SrCl_2$, and 3 mM $BaCl_2$ (Fig. S7). We observed similar upturns at low q in corresponding Guinier plots, indicating RTX-v aggregation. At these intermediate metal chloride concentrations, partially folded RTX-v appears more prone to aggregation than its fully disordered or structured states. We attribute this aggregation to solvent-exposed hydrophobic residues that are more likely to form intermolecular associations.

Dimensionless Kratky plots reveal that RTX-v is partially flexible in its unfolded and folded states. Across all scattering profiles, an increase in $I(q)q^2$ at high q is characteristic of a protein with a flexible region (Fig. 2 *A*) (63). Without divalent cations, the Kratky plot best matches a primarily disordered protein, with a shallow peak suggesting locally folded regions (58). Addition of $MgCl_2$ slightly distinguishes the shallow peak while maintaining the flexible high-q region. In the presence of 1 mM and 3 mM $CaCl_2$, a sharp peak indicates the protein is compacted upon adopting the RTX-v-Ca^{2+} structure. The same features appear with 10 mM $SrCl_2$.

With only 1 mM $SrCl_2$, RTX-v is slightly compacted without adopting the folded structure, consistent with the trend in R_g. Compact protein features become more pronounced at intermediate concentrations of 2 mM and 3 mM $SrCl_2$. Addition of 1 mM $BaCl_2$ widens the low q peak with respect to 1 mM $CaCl_2$ and 1 mM $SrCl_2$. This peak is more pronounced than in the cation-free and $MgCl_2$ conditions and weaker than with Ca^{2+} or 10 mM $SrCl_2$. The peak magnitude and sharpness are enhanced with 3 mM or 10 mM $BaCl_2$, indicating partial compaction of the protein (63). The partial compaction of RTX-v with 1–3 mM $SrCl_2$ or 1–10 mM $BaCl_2$ warranted further analysis to distinguish between protein size changes and structure formation.

To further resolve the compacted protein states, we reconstructed 3D electron density maps from scattering profiles using the DENSS algorithm (40) (Fig. 2 *B*). In the absence of divalent cations, the reconstructed electron density map for RTX-v indicates a flexible and disordered conformation (data not shown). The electron density maps remain spatially extended upon adding 1 mM and 10 mM $MgCl_2$. With 1 mM $CaCl_2$ or 10 mM $SrCl_2$, the electron density is consistent with the slightly elongated shape of the protein crystal structure. An elongated shape is expected from the repetitive structure of RTX-v-Ca^{2+} (18,64). The electron density with 10 mM $BaCl_2$ is more elongated than RTX-v-Ca^{2+} with a similar width, suggesting partial β-roll formation while part of the protein remains disordered. All R_g values computed directly from DENSS reconstructions agree with the Guinier fits (Table S2).

Pair-distance distribution functions $P(r)$ of intermediate RTX-v conformations show comparable length scales to folded proteins while retaining an extended and flexible region (Fig. 2 *C*). Unfolded RTX-v with no divalent cations, 1 mM, or 10 mM $MgCl_2$ produces a broad pair-distance distribution with a large maximum protein dimension D_{max} (15.4 nm, 14.9 nm, and 16.3 nm, respectively). Shallow shoulders indicate disorder (63). These shoulders disappear with 1 mM and 3 mM $CaCl_2$, or 10 mM $SrCl_2$ (D_{max} = 8.1 nm, 8.8 nm, and 10.1 nm). In these conditions, $P(r)$ is more symmetric and narrow, characteristic of folded proteins. Both unfolded and folded features emerge in the intermediate RTX-v states with 1 mM $SrCl_2$, or 1 mM and 10 mM $BaCl_2$ (D_{max} = 13.0 nm, 13.1 nm, and 11.7 nm). In these conditions, $P(r)$ is narrower than for disordered proteins, with a shallow shoulder and trailing tail indicating a partially extended conformation. All R_g values computed from $P(r)$ agree with the Guinier fits and DENSS reconstructions (Table S2).

Sr^{2+} stabilizes RTX-v β-roll structure

X-ray structures of RTX-v-Ca^{2+} and RTX-v-Sr^{2+} are remarkably similar (Fig. 3 *A*). Structural similarity was quantified by the root-mean-square distance of 0.25 Å. In RTX-v-Sr^{2+}, all eight canonical Ca^{2+}-binding sites in RTX-v contained Sr^{2+} with identical coordinating ligands and geometries. Coordinating residues are highlighted throughout the RTX-v sequence in Fig. 3 *A*. Prior studies have crystallized other RTX domains with Sr^{2+} (65); to the authors' knowledge, this structure is the first report of CyaA RTX-v bound to Sr^{2+}. We assessed the quality of agreement between RTX-v-Ca^{2+} and experimental SAXS

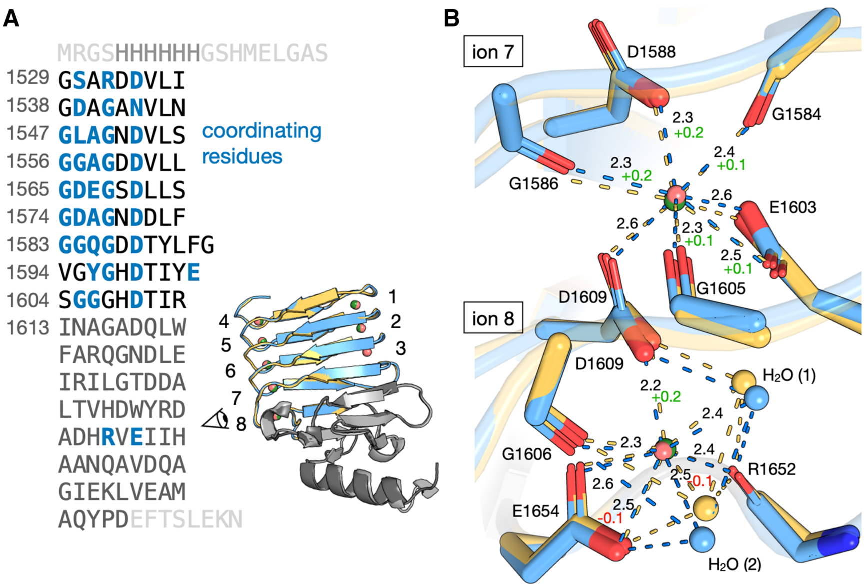

FIGURE 3 Both Ca^{2+} and Sr^{2+} stabilize the folded RTX-v structure with identical coordinating geometries. (*A*) The native RTX-v sequence and superimposed Ca^{2+}-bound (*blue*) and Sr^{2+}-bound (*yellow*) structures, with coordinating residues in blue font (*bolded*). The C-terminal capping domain is highlighted in gray, and the repeat ion-binding structure is shown in blue/yellow. Ca^{2+} and Sr^{2+} ions are shown as pink and green spheres, respectively. A cartoon eye indicates the viewer perspective in (*B*). (*B*) Close-up view of seventh- and eighth-position ion binding loops for the overlaid RTX-v-Ca^{2+} and RTX-v-Sr^{2+} structures. Ca^{2+} and Sr^{2+} ions are depicted with relative sizes proportional to their seven-coordinate ionic radii. Noncoordinating residues are omitted for clarity. Coordination and hydrogen bonds are shown as blue or yellow dashed lines, and the two water molecules involved in the coordination of the eighth ion are shown as blue or yellow spheres. Ion–ligand distances (Å) for the RTX-v-Ca^{2+} structure are annotated in black font. Changes in distances for the Sr^{2+}-bound structures are indicated in green (increased) or red (decreased) text. Shifts in solvent molecule positions at the eighth ion site allow RTX-v to accommodate the larger Sr^{2+} ion.

profiles using the FoXS webserver (66). The theoretical scattering profile from RTX-v-Ca^{2+} agreed well with data collected at 3 mM $CaCl_2$ and 10 mM $SrCl_2$ (Fig. S14). Crystallization attempts of RTX-v incubated with up to 10 mM $MgCl_2$ or 10 mM $BaCl_2$ did not yield diffraction-quality crystals containing both protein and cation.

For RTX-v-Sr^{2+} to accommodate the larger strontium ion, its coordinating backbone carbonyls and side-chain carboxyls shift positions (Fig. S12). At the solvent-exposed, C-terminal ion-binding site—denoted as Sr^{2+}[8]—two coordinating water molecules and the side-chain carboxyls of Asp1609 and Glu1654 are rotated when compared with Ca^{2+}[8]. Typically, these carboxyls and the backbone carbonyls of Gly1606 and Arg1652 bind to Ca^{2+}[8] to nucleate folding of the RTX-v capping domain and stabilize the β-roll structure (18). This mechanism and structure appear to be preserved during Sr^{2+} binding, except the Asp1609 side-chain carboxyl is positioned 0.2 Å farther from Sr^{2+}[8] than from Ca^{2+}[8]. Conversely, the Glu1654 carboxyl and the second coordinating water molecule H_2 O(2) both shift 0.1 Å closer to Sr^{2+}[8]. Measurements of all ion-ligand distances in both structures reveal that position shifts are most pronounced at the seventh ion-binding site, where five of seven coordinating carbonyls or carboxyls are 0.1 or 0.2 Å farther from Sr^{2+}[7] than Ca^{2+}[7] (Fig. 3 *B*; Table S8).

Most of the remaining ion-binding sites of RTX-v expand slightly to accommodate larger Sr^{2+}, with less pronounced distance changes toward the N-terminal ion-binding sites (Fig. 4). The average changes in ion-ligand distances for Sr^{2+}[1] to Sr^{2+}[5] range from −0.02 Å to +0.03 Å, in contrast to average increases of +0.07 Å and +0.10 Å for Sr^{2+}[6] and Sr^{2+}[7], respectively. We attribute smaller N-terminal changes to increased protein flexibility in its unbound, disordered state, which enables larger structural shifts near the folding nucleation site of the eighth, C-terminal ion-binding site. Expanded ion-binding sites for Sr^{2+}[6] and Sr^{2+}[7] force some β-roll turns to be farther away from each other than with Ca^{2+}. Modest displacements of turns surrounding Sr^{2+}[2] and Sr^{2+}[5] are measured as the distances between α-carbons above and below the ion site (Fig. S13; Table S9). Turns surrounding Sr^{2+}[2] and Sr^{2+}[5] increased by an average of 0.1 Å and 0.25 Å, respectively, compared with RTX-v-Ca^{2+} (Table S8). Less significant changes around the other ions imply fewer constraints at the edges of the β-roll structure.

We hypothesized that Sr^{2+} would nucleate folding in RTX-v due to its similarities to Ca^{2+} (67,68), but we did not anticipate such subtle expansion of ion-binding loops upon Sr^{2+} substitution. Indeed, solvent-exposed, flexible Ca^{2+}-binding sites are prone to Sr^{2+} substitution (69). Ca-binding sites fitting these criteria are found in alkaline

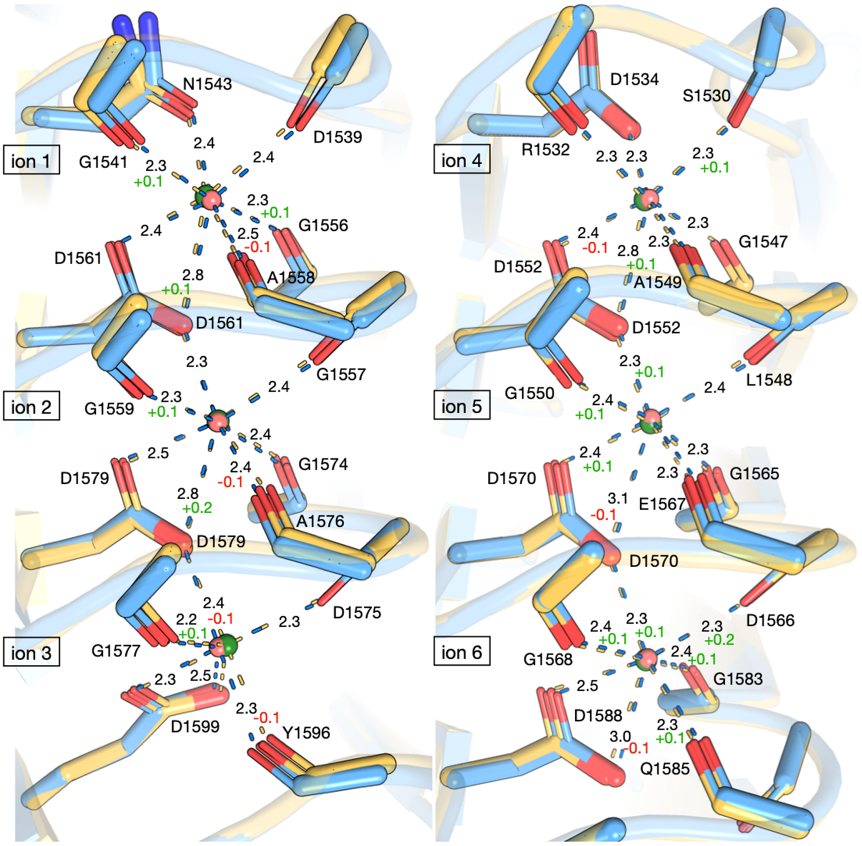

FIGURE 4 Shifts in coordinating ligand positions between the Ca^{2+}-bound (*blue*) and Sr^{2+}-bound (*yellow*) structures are less pronounced toward the N-terminus of the protein (*top*, ions 1 and 4). A close-up view of the first- through sixth-position ion-binding loops is shown for the overlaid protein structures. Ca^{2+} and Sr^{2+} are shown as pink and green spheres, respectively, with relative sizes proportional to their seven-coordinate ionic radii. Nucleation of ion-dependent folding at the C-terminus reduces flexibility at the N-terminus.

phosphatase (70), parvalbumin (71), and bacterial surface layer proteins (65), wherein Sr^{2+} successfully substitutes for Ca^{2+} with nearly identical ion-ligand coordination. Our results suggest the intrinsic flexibility of apo-RTX-v and presence of two coordinating waters enable promiscuous binding at Sr^{2+}[8]. We attribute the similarity between RTX-v-Ca^{2+} and RTX-v-Sr^{2+} to the repetitive structure of RTX-v, which forces Sr^{2+} to adopt identical coordinating geometries as Ca after the nucleation of folding at the C-terminal ion-binding site. The binding site geometries are further rigidified by $\delta 2$ oxygens of carboxylic acids, which coordinate two ions simultaneously (72). As the protein successively folds from C- to N-termini, the repetitive sequence constrains available ion-ligand distances, forcing near-identical folds of RTX-v-Sr and RTX-v-Ca^{2+}. These tight constraints may disallow RTX-v folding in the presence of ions with drastically different sizes, including Mg^{2+} and Ba^{2+}.

RTX-v secondary structure formation is tuned by ion

CD spectroscopy reveals a fivefold stronger affinity of RTX-v to Ca^{2+} over Sr^{2+} (Fig. 5). In the absence of divalent cations, a negative peak at 200 nm indicates a disordered protein. Upon addition of at least 1 mM $CaCl_2$, RTX-v formed β-roll structures indicated by the appearance of a negative peak at 218 nm and the disappearance of the negative peak at 200 nm (17,27,73). Upon addition of at least 10 mM $SrCl_2$, RTX-v underwent the same structural transition from disordered to a β-roll, with spectra matching those of RTX-v-Ca^{2+}. The binding affinities of RTX-v-Ca^{2+} and RTX-v-Sr^{2+} were compared using the Hill-Langmuir equation, which determines apparent disassociation constants K_D and Hill coefficients n (Fig. S10) (74). For Ca, K_D of 0.69 $\pm$ 0.03 mM and n of 4.4 $\pm$ 0.5 agree with previous results (17,27,28). For Sr^{2+}, K_D and n of 3.3 $\pm$ 0.1 mM and 1.5 $\pm$ 0.1 indicated weaker affinity with less cooperative binding.

Mg^{2+} and Ba^{2+} induce subtle structural changes in RTX-v. The disordered 200-nm peak weakened slightly upon introducing at least 10 mM $MgCl_2$ or 10 mM $BaCl_2$. With both ions, a shoulder emerged between 210 nm and 230 nm, which suggested β-structure formation. Spectral deconvolution quantified a modest increase in β-sheet content from 13.2% (50 mM Tris) to 16.2% (100 mM $MgCl_2$) or a more dramatic increase to 21.1% (100 mM $BaCl_2$) (57). Together, these features indicate that Mg^{2+} and Ba^{2+} may occupy a small number of C-terminal ion binding sites without driving the full structural transition of RTX-v.

Affinity of RTX-v to different divalent cations is heavily influenced by the distinct sizes and coordination preferences of each ion. We hypothesize the reduced affinity of RTX-v to Sr^{2+} over Ca^{2+} is partially due to the lower charge density of Sr^{2+}, given its larger ionic radius than Ca^{2+} but similar coordination preferences (71,75). Mg^{2+} has a higher charge density, suggesting that aspartic acid-rich RTX-v might more easily recruit the ion, but it prefers an octahedral coordination and smaller binding sites (72,76). In Ca^{2+}-binding EF-hand proteins, Mg^{2+} can occupy Ca^{2+} sites with the aid of additional coordinating waters (77,78). Given the two coordinating water molecules at Ca^{2+}[8]/Sr^{2+}[8], it is plausible Mg^{2+} could occupy this binding site without stabilizing the rest of the β-roll structure, since the remaining binding sites are buried and thus inaccessible to coordinating waters.

DISCUSSION

To gain insights into the origins of metal selectivity in disordered proteins, we characterized ion-induced conformational changes of the Ca^{2+}-binding RTX-v domain. Ions beyond Ca^{2+} can occupy Ca^{2+}-binding sites in RTX-v, but the mechanisms dictating how RTX-v promiscuously binds other ions are unclear (32,79). Probing conformational changes in RTX-v and other ion-binding proteins is often used as a proxy for metal binding while providing critical insight into how ions may stabilize different protein conformations (27,80). We find that RTX-v adopts diverse conformations upon incubation with group II ions with similar chemical characteristics as natively binding Ca^{2+}.

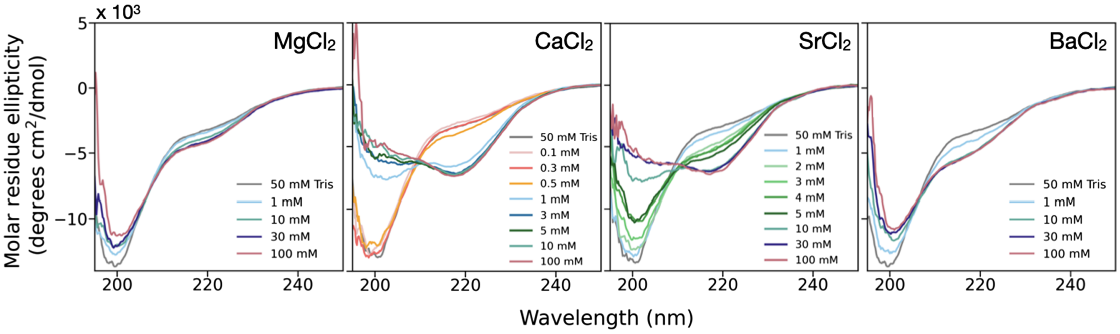

FIGURE 5 Circular dichroism experiments reveal secondary structure formation in RTX-v in response to group II ions. CD spectra were collected with RTX-v incubated with 0.1–100 mM metal chlorides. At 1 mM and 10 mM, respectively, $CaCl_2$ and $SrCl_2$ induce β-roll structure formation indicated by a negative peak at 218 nm. A slight shoulder between 210 and 230 nm appears upon incubation with 10 mM $MgCl_2$ or $BaCl_2$.

We demonstrate that RTX-v adopts its native fold upon Sr^{2+} substitution by determining the atomic structure of Sr^{2+}-bound RTX-v. Although RTX-v-Ca^{2+} and RTX-v-Sr^{2+} are nearly identical, RTX-v binds to Sr^{2+} with fivefold less affinity than to Ca^{2+} (Fig. S10). Slight increases in ion-ligand distances in RTX-v-Sr^{2+} are required for Sr^{2+} binding, consistent with simulations of an extracellular calcium-sensing receptor (75).

CD reveals modest secondary structure formation of RTX-v upon incubation with Mg^{2+} and Ba^{2+}. Mg^{2+} did not induce compaction in the protein as measured by SAXS, whereas Ba^{2+} reduced RTX-v R_g compared with its disordered state. The compaction of RTX-v with 10 mM $BaCl_2$ suggests that Ba^{2+} can stabilize partial folding of the protein. In contrast to the reduced R_g also seen with 1–3 mM $SrCl_2$, a distinct mechanism underlies Ba^{2+}-induced compaction, as only modest secondary structure formation was observed in CD spectra up to 100 mM $BaCl_2$.

Flexibility in apo-RTX-v enables promiscuous binding

Flexibility of the disordered apo-RTX-v enables initial binding at Sr^{2+}[8], where water molecules shift positions to accommodate Sr^{2+}. The repetitive structure of RTX-v then restricts binding promiscuity by constraining the available coordination geometry of the remaining binding sites. RTX-v-Ca^{2+} and RTX-v-Sr^{2+} form nearly identical structures as a result, as Sr^{2+} is forced to favor identical coordinating ligands and geometries. At the intermediate 1–3 mM $SrCl_2$ concentrations, RTX-v adopts a conformation in between its disordered and folded states, suggesting a dynamic equilibrium or partially folded state (Figs. 1 *B*, 2, and S7) (81).

Conformational flexibility may allow RTX-v to partially fold in response to Mg^{2+} and Ba^{2+}. SAXS and CD data reveal that partial folding may be nucleated with Mg^{2+} and Ba^{2+}, evidenced by size changes and slight secondary structure formation, whereas a portion of the protein remains flexible (Fig. 2). We attribute Ba^{2+}-induced compaction to direct coordination of Ba^{2+}, as opposed to compaction in response to increased ionic strength. Scattering profiles of RTX-v with 10 mM KCl exactly matched that of the cation-free sample (Fig. S2). Likewise, CD spectra collected with 10 mM KCl and 10 mM NaCl matched the cation-free spectra (Fig. S9).

DENSS reconstructions of electron density maps show the compact Ba^{2+}-induced conformation is more similar to RTX-v-Ca^{2+} and RTX-v-Sr^{2+} than samples with Mg^{2+}. Ba^{2+}-induced compaction was easily observed via a change in R_g of 2.7 nm versus 3.8 nm in the cation-free sample. We hypothesize that Ba^{2+} stabilizes one or more ion-binding turns at the C-terminus, but that the ion is too large for full β-roll formation. This hypothesis explains how a portion of the protein remains flexible (as seen in Kratky plots), whereas the $P(r)$ distribution more closely resembles the folded protein structure than the disordered structure. Ba^{2+} has been shown to substitute for Ca^{2+}-binding sites in EF-hand proteins with similar coordinating ligands (82).

The behavior of RTX-v with Ba^{2+} is distinct from with Mg^{2+}, where very little compaction is observed in SAXS profiles, and the protein remains flexible upon incubation with $MgCl_2$. DENSS reconstructions show a largely extended conformation of RTX-v with Mg^{2+}, in line with a disordered structure. Prior studies suggest Mg^{2+} may occupy binding sites in RTX-v, explaining the very subtle changes in CD spectra (30,83).

CONCLUSIONS

The factors dictating how proteins selectively bind metal ions differ widely between environmental fluctuations, protein sequence and structural constraints, and specific ionic properties (6). In IDPs that undergo ion-dependent folding, elucidating mechanisms of metal ion selectivity is especially challenging. To provide insight into how proteins coordinate ions in order to fold, we investigated ion-dependent conformations in an intrinsically disordered Ca^{2+}-binding protein from the RTX family. We find that the flexibility and disorder of RTX-v promote promiscuous ion binding to Sr^{2+}, which successfully substitutes all Ca^{2+}-binding sites. For smaller Mg and larger Ba^{2+}, the tightly constrained coordination geometry conferred from the repetitive sequence of RTX-v prevents full recovery of its folded structure. Critically, we show that Ba^{2+} induces a partially folded conformation, yielding insights into how off-target ion binding may result in protein misfolding. Many open questions remain, including quantifying Ba^{2+} binding to RTX-v and exploring sequence mutation effects on ion coordination. Sequence-to-structure prediction programs like AlphaFold3 can predict biomolecular interactions (84), but they often struggle to capture partially folded conformations in the presence of metal ions (Fig. S15). We anticipate future investigations with protein solution nuclear magnetic resonance (18,85) or molecular dynamics simulations (86,87) would provide powerful insights into partially folded conformations of RTX-v with Ba^{2+} by identifying coordinating residues. Overall, this work lays important groundwork for future studies investigating how conformational states of IDPs influence ion selectivity.

DATA AND CODE AVAILABILITY

SAXS data are deposited in the Small Angle Scattering Biological Data Bank (SASBDB: SASDXM8, SASDXN8, SASDXP8, SASDXQ8, SASDXR8, SASDXS8, SASDXT8, SASDXU8, SASDXV8, SASDXW8, SASDXX8, SASDXY8, and SASDXZ8). CD data are deposited in the Protein Circular Dichrosim Data Bank (PCDDB) at pcddb.cryst.bbk.ac.uk/. The x-ray structures are available in the Protein Data Bank (PDB: 9P0C and 9P0D).

ACKNOWLEDGMENTS

This material is based upon work supported by the Air Force Office of Scientific Research under award number FA9550-22-1-0241. We acknowledge

training program support by the Macromolecular Structure Knowledge Center (MSKC) at Sarafan ChEM-H (RRID: SCR_023233) and the Stanford Community of Shared Research Platforms (C-ShaRP) Experiential Learning Program (RRID: SCR_022986). A.P.G. is partially supported by the National Science Foundation Graduate Research Fellowship Program (DGE-1656518). We thank Prof. Possu Huang for access to the CD spectrophotometer and Dr. Olivia N. Pattelli for conducting crystallographic screens (Macromolecular Structure Group, Nucleus at Sarafan ChEM-H). We also thank Dr. Thomas Weiss (SSRL/SLAC), Dr. Patrick Dennis (Air Force Research Laboratory), Prof. Soichi Wakatsuki, and all members of the Mai Lab for helpful conversations.

Use of the Stanford Synchrotron Radiation Lightsource, SLAC National Accelerator Laboratory, is supported by the US Department of Energy, Office of Science, Office of Basic Energy Sciences under contract no. DE-AC02-76SF00515. The SSRL Structural Molecular Biology Program is supported by the DOE Office of Biological and Environmental Research and by the National Institutes of Health, National Institute of General Medical Sciences (P30GM133894). The contents of this publication are solely the responsibility of the authors and do not necessarily represent the official views of NIGMS or NIH.

AUTHOR CONTRIBUTIONS

A.P.G., G.M.S., M.P.C., and D.J.M. conceptualized the project. A.P.G., G.M.S., M.P.C., D.F., and T.M. conducted the experiments. A.P.G., D.F., T.M., and D.J.M. analyzed the results. A.P.G. and D.J.M. wrote the manuscript. All authors reviewed the manuscript.

DECLARATION OF INTERESTS

The authors declare no competing interests.

SUPPORTING CITATIONS

References (88–90) appear in the supporting material.

SUPPORTING MATERIAL

Supporting material can be found online at https://doi.org/10.1016/j.bpj.2025.10.014.

REFERENCES

1. Dunker, A. K., J. D. Lawson, …, Z. Obradovic. 2001. Intrinsically disordered protein. *J. Mol. Graph. Model.* 19:26–59. https://doi.org/10.1016/S1093-3263(00)00138-8.
2. Dyson, H. J., and P. E. Wright. 2005. Intrinsically unstructured proteins and their functions. *Nat. Rev. Mol. Cell Biol.* 6:197–208. https://doi.org/10.1038/nrm1589.
3. Moses, D., G. M. Ginell, …, S. Sukenik. 2023. Intrinsically disordered regions are poised to act as sensors of cellular chemistry. *Trends Biochem. Sci.* 48:1019–1034. https://doi.org/10.1016/j.tibs.2023.08.001.
4. Wright, P. E., and H. J. Dyson. 2009. Linking folding and binding. *Curr. Opin. Struct. Biol.* 19:31–38. https://doi.org/10.1016/j.sbi.2008.12.003.
5. Arai, M., K. Sugase, …, P. E. Wright. 2015. Conformational propensities of intrinsically disordered proteins influence the mechanism of binding and folding. *Proc. Natl. Acad. Sci. USA.* 112:9614–9619. https://doi.org/10.1073/pnas.1512799112.
6. Dudev, T., and C. Lim. 2014. Competition among metal ions for protein binding sites: Determinants of metal ion selectivity in proteins. *Chem. Rev.* 114:538–556. https://doi.org/10.1021/cr4004665.
7. Newcombe, E. A., C. B. Fernandes, …, B. B. Kragelund. 2021. Insight into Calcium-Binding Motifs of Intrinsically Disordered Proteins. *Biomolecules.* 11:1173. https://doi.org/10.3390/biom11081173.
8. Wicky, B. I. M., S. L. Shammas, and J. Clarke. 2017. Affinity of IDPs to their targets is modulated by ion-specific changes in kinetics and residual structure. *Proc. Natl. Acad. Sci. USA.* 114:9882–9887. https://doi.org/10.1073/pnas.1705105114.
9. Tamás, M. J., B. Fauvet, …, P. Goloubinoff. 2018. Misfolding and aggregation of nascent proteins: a novel mode of toxic cadmium action in vivo. *Curr. Genet.* 64:177–181. https://doi.org/10.1007/s00294-017-0748-x.
10. de Souza, I. D., A. S. de Andrade, and R. J. S. Dalmolin. 2018. Lead-interacting proteins and their implication in lead poisoning. *Crit. Rev. Toxicol.* 48:375–386. https://doi.org/10.1080/10408444.2018.1429387.
11. Witkowska, D., J. Słowik, and K. Chilicka. 2021. Review heavy metals and human health: Possible exposure pathways and the competition for protein binding sites. *Molecules.* 26:6060. https://doi.org/10.3390/molecules26196060.
12. Uversky, V. N. 2007. Neuropathology, biochemistry, and biophysics of alpha-synuclein aggregation. *J. Neurochem.* 103:17–37. https://doi.org/10.1111/j.1471-4159.2007.04764.x.
13. Faller, P., C. Hureau, and G. La Penna. 2014. Metal Ions and Intrinsically Disordered Proteins and Peptides: From Cu/Zn Amyloid-β to General Principles. *Acc. Chem. Res.* 47:2252–2259. https://doi.org/10.1021/ar400293h.
14. Breydo, L., and V. N. Uversky. 2011. Role of metal ions in aggregation of intrinsically disordered proteins in neurodegenerative diseases. *Metallomics.* 3:1163–1180. https://doi.org/10.1039/c1mt00106j.
15. Waldron, K. J., and N. J. Robinson. 2009. How do bacterial cells ensure that metalloproteins get the correct metal? *Nat. Rev. Microbiol.* 7:25–35. https://doi.org/10.1038/nrmicro2057.
16. Mazmanian, K., K. Sargsyan, and C. Lim. 2020. How the Local Environment of Functional Sites Regulates Protein Function. *J. Am. Chem. Soc.* 142:9861–9871. https://doi.org/10.1021/jacs.0c02430.
17. Bauche, C., A. Chenal, …, D. Ladant. 2006. Structural and Functional Characterization of an Essential RTX Subdomain of Bordetella pertussis Adenylate Cyclase Toxin. *J. Biol. Chem.* 281:16914–16926. https://doi.org/10.1074/jbc.M601594200.
18. Bumba, L., J. Masin, …, P. Sebo. 2016. Calcium-Driven Folding of RTX Domain β-Rolls Ratchets Translocation of RTX Proteins through Type I Secretion Ducts. *Mol. Cell.* 62:47–62. https://doi.org/10.1016/j.molcel.2016.03.018.
19. Shannon, R. D. 1976. Revised effective ionic radii and systematic studies of interatomic distances in halides and chalcogenides. *Acta Cryst. A.* 32:751–767. https://doi.org/10.1107/S0567739476001551.
20. Gangola, P., and B. P. Rosen. 1987. Maintenance of intracellular calcium in Escherichia coli. *J. Biol. Chem.* 262:12570–12574. https://doi.org/10.1016/S0021-9258(18)45243-X.
21. Bulutoglu, B., and S. Banta. 2017. Block V RTX domain of adenylate cyclase from Bordetella pertussis: A conformationally dynamic scaffold for protein engineering applications. *Toxins.* 9:289. https://doi.org/10.3390/toxins9090289.
22. Wang, H., X. Gao, and H. Li. 2019. Single Molecule Force Spectroscopy Reveals the Mechanical Design Governing the Efficient Translocation of the Bacterial Toxin Protein RTX. *J. Am. Chem. Soc.* 141:20498–20506. https://doi.org/10.1021/jacs.9b11281.
23. Motlova, L., N. Klimova, …, L. Bumba. 2020. Continuous Assembly of Beta-Roll Structures Is Implicated in the Type I-Dependent Secretion of Large Repeat-in-Toxins (RTX) Proteins. *J. Mol. Biol.* 432:5696–5710. https://doi.org/10.1016/j.jmb.2020.08.020.
24. Wang, H., G. Chen, and H. Li. 2022. Templated folding of the RTX domain of the bacterial toxin adenylate cyclase revealed by single molecule force spectroscopy. *Nat. Commun.* 13:2784. https://doi.org/10.1038/s41467-022-30448-8.
25. Sotomayor-Pérez, A.-C., D. Ladant, and A. Chenal. 2014. Disorder-to-Order Transition in the CyaA Toxin RTX Domain: Implications

for Toxin Secretion. *Toxins.* 7:1–20. https://doi.org/10.3390/toxins7010001.

26. Knapp, O., E. Maier, …, R. Benz. 2003. Channel Formation in Model Membranes by the Adenylate Cyclase Toxin of *Bordetella pertussis* : Effect of Calcium. *Biochemistry.* 42:8077–8084. https://doi.org/10.1021/bi034295f.
27. Szilvay, G. R., M. A. Blenner, …, S. Banta. 2009. A FRET-based method for probing the conformational behavior of an intrinsically disordered repeat domain from Bordetella pertussis adenylate cyclase. *Biochemistry.* 48:11273–11282. https://doi.org/10.1021/bi901447j.
28. Chang, M. P., W. Huang, …, D. J. Mai. 2024. Sequence-defined structural transitions by calcium-responsive proteins. *Polym. Chem.* 15:4864–4874. https://doi.org/10.1039/D4PY00907J.
29. Baumann, U. 2019. Structure-function relationships of the repeat domains of RTX toxins. *Toxins.* 11:657. https://doi.org/10.3390/toxins11110657.
30. Rhodes, C. R., M. C. Gray, …, C. M. Grisham. 2001. Structural Consequences of Divalent Metal Binding by the Adenylyl Cyclase Toxin of Bordetella pertussis. *Arch. Biochem. Biophys.* 395:169–176. https://doi.org/10.1006/abbi.2001.2553.
31. Scotter, A. J., M. Guo, …, P. L. Davies. 2007. Metal ion-dependent, reversible, protein filament formation by designed beta-roll polypeptides. *BMC Struct. Biol.* 7:63. https://doi.org/10.1186/1472-6807-7-63.
32. Khoury, F., Z. Su, and S. Banta. 2024. Rare Earth Element Binding and Recovery by a Beta Roll-Forming RTX Domain. *Inorg. Chem.* 63:13223–13230. https://doi.org/10.1021/acs.inorgchem.4c00420.
33. Kloss, E., N. Courtemanche, and D. Barrick. 2008. Repeat-protein folding: New insights into origins of cooperativity, stability, and topology. *Arch. Biochem. Biophys.* 469:83–99. https://doi.org/10.1016/j.abb.2007.08.034.
34. Matsui, T., I. Rajkovic, …, T. M. Weiss. 2024. Adaptable SEC-SAXS data collection for higher quality structure analysis in solution. *Protein Sci.* 33:e4946. https://doi.org/10.1002/pro.4946.
35. Choi, K. H., and M. Morais. 2014. Use of Small-Angle X-Ray Scattering to Investigate the Structure and Function of Dengue Virus NS3 and NS5. Springer New York, New York, NY, pp. 241–252.
36. Petoukhov, M. V., P. V. Konarev, …, D. I. Svergun. 2007. *ATSAS* 2.1 – Towards automated and web-supported small-angle scattering data analysis. *J. Appl. Crystallogr.* 40:s223–s228. https://doi.org/10.1107/S0021889807002853.
37. Hopkins, J. B. 2024. *BioXTAS RAW 2*: New developments for a free open-source program for small-angle scattering data reduction and analysis. *J. Appl. Crystallogr.* 57:194–208. https://doi.org/10.1107/S1600576723011019.
38. Konarev, P. V., V. V. Volkov, …, D. I. Svergun. 2003. *PRIMUS*: a Windows PC-based system for small-angle scattering data analysis. *J. Appl. Crystallogr.* 36:1277–1282. https://doi.org/10.1107/S0021889803012779.
39. Svergun, D. I. 1992. Determination of the regularization parameter in indirect-transform methods using perceptual criteria. *J. Appl. Crystallogr.* 25:495–503. https://doi.org/10.1107/S0021889892001663.
40. Grant, T. D. 2018. Ab initio electron density determination directly from solution scattering data. *Nat. Methods.* 15:191–193. https://doi.org/10.1038/nmeth.4581.
41. Smith, C. A., G. L. Card, …, S. M. Soltis. 2010. Remote access to crystallography beamlines at SSRL: novel tools for training, education and collaboration. *J. Appl. Crystallogr.* 43:1261–1270. https://doi.org/10.1107/S0021889810024696.
42. Kabsch, W. 2010. XDS. *Acta Crystallogr. D.* 66:125–132. https://doi.org/10.1107/S0907444909047337.
43. Evans, P. R., and G. N. Murshudov. 2013. How good are my data and what is the resolution? *Acta Crystallogr. D.* 69:1204–1214. https://doi.org/10.1107/S0907444913000061.
44. McCoy, A. J., R. W. Grosse-Kunstleve, …, R. J. Read. 2007. *Phaser* crystallographic software. *J. Appl. Crystallogr.* 40:658–674. https://doi.org/10.1107/S0021889807021206.
45. Murshudov, G. N., A. A. Vagin, and E. J. Dodson. 1997. Refinement of Macromolecular Structures by the Maximum-Likelihood Method. *Acta Crystallogr. D.* 53:240–255. https://doi.org/10.1107/S0907444996012255.
46. Emsley, P., B. Lohkamp, …, K. Cowtan. 2010. Features and development of *Coot*. *Acta Crystallogr. D.* 66:486–501. https://doi.org/10.1107/S0907444910007493.
47. Winter, G., D. G. Waterman, …, G. Evans. 2018. *DIALS*: implementation and evaluation of a new integration package. *Acta Crystallogr. D Struct. Biol.* 74:85–97. https://doi.org/10.1107/S2059798317017235.
48. Evans, P. 2006. Scaling and assessment of data quality. *Acta Crystallogr. D.* 62:72–82. https://doi.org/10.1107/S0907444905036693.
49. González, A., P. Moorhead, …, S. M. Soltis. 2008. *Web-Ice*: integrated data collection and analysis for macromolecular crystallography. *J. Appl. Crystallogr.* 41:176–184. https://doi.org/10.1107/S0021889807057822.
50. Skubák, P. 2018. Substructure determination using phase-retrieval techniques. *Acta Crystallogr. D.* 74:117–124. https://doi.org/10.1107/S2059798317014462.
51. Sheldrick, G. M. 2010. Experimental phasing with *SHELXC/D/E*: combining chain tracing with density modification. *Acta Crystallogr. D.* 66:479–485. https://doi.org/10.1107/S0907444909038360.
52. Stein, P. E., A. Boodhoo, …, R. J. Read. 1994. The crystal structure of pertussis toxin. *Structure.* 2:45–57. https://doi.org/10.1016/S0969-2126(00)00007-1.
53. Abrahams, J. P., and A. G. Leslie. 1996. Methods used in the structure determination of bovine mitochondrial F 1ATPase. *Acta Crystallogr. D.* 52:30–42. https://doi.org/10.1107/S0907444995008754.
54. Cowtan, K. 2006. The *Buccaneer* software for automated model building. 1. Tracing protein chains. *Acta Crystallogr. D.* 62:1002–1011. https://doi.org/10.1107/S0907444906022116.
55. Winn, M. D., C. C. Ballard, …, K. S. Wilson. 2011. Overview of the *CCP*4 suite and current developments. *Acta Crystallogr. D.* 67:235–242. https://doi.org/10.1107/S0907444910045749.
56. Hildebrandt, C., L. Joos, …, G. Winter. 2015. The "New Polyethylene Glycol Dilemma": Polyethylene Glycol Impurities and Their Paradox Role in mAb Crystallization. *J. Pharmacol. Sci.* 104:1938–1945. https://doi.org/10.1002/jps.24424.
57. Sreerama, N., and R. W. Woody. 2000. Estimation of Protein Secondary Structure from Circular Dichroism Spectra: Comparison of CONTIN, SELCON, and CDSSTR Methods with an Expanded Reference Set. *Anal. Biochem.* 287:252–260. https://doi.org/10.1006/abio.2000.4880.
58. O'Brien, D. P., B. Hernandez, …, A. Chenal. 2015. Structural models of intrinsically disordered and calcium-bound folded states of a protein adapted for secretion. *Sci. Rep.* 5:14223. https://doi.org/10.1038/srep14223.
59. O'Brien, D. P., S. Brier, …, P. Vachette. 2018. SEC-SAXS and HDX-MS: A powerful combination. The case of the calcium-binding domain of a bacterial toxin. *Biotechnol. Appl. Biochem.* 65:62–68. https://doi.org/10.1002/bab.1577.
60. Skou, S., R. E. Gillilan, and N. Ando. 2014. Synchrotron-based small-angle X-ray scattering of proteins in solution. *Nat. Protoc.* 9:1727–1739. https://doi.org/10.1038/nprot.2014.116.
61. Sotomayor-Pérez, A. C., D. Ladant, and A. Chenal. 2011. Calcium-induced folding of intrinsically disordered Repeat-in-Toxin (RTX) motifs via changes of protein charges and oligomerization states. *J. Biol. Chem.* 286:16997–17004. https://doi.org/10.1074/jbc.M110.210393.
62. Zhang, L., J. Franks, …, P. H. Thibodeau. 2014. Inducible Polymerization and Two-Dimensional Assembly of the Repeats-in-Toxin (RTX) Domain from the Pseudomonas aeruginosa Alkaline Protease. *Biochemistry.* 53:6452–6462. https://doi.org/10.1021/bi5007546.
63. Receveur-Brechot, V., and D. Durand. 2012. How Random are Intrinsically Disordered Proteins? A Small Angle Scattering Perspective. *Curr. Protein Pept. Sci.* 13:55–75. https://doi.org/10.2174/138920312799277901.
64. Zhang, Y., M. Berghaus, …, C. A. Royer. 2018. High-Pressure NMR and SAXS Reveals How Capping Modulates Folding Cooperativity

of the pp32 Leucine-rich Repeat Protein. *J. Mol. Biol.* 430:1336–1349. https://doi.org/10.1016/j.jmb.2018.03.005.

65. Herrmann, J., P.-N. Li, …, S. Wakatsuki. 2020. A bacterial surface layer protein exploits multistep crystallization for rapid self-assembly. *Proc. Natl. Acad. Sci. USA.* 117:388–394. https://doi.org/10.1073/pnas.1909798116.

66. Schneidman-Duhovny, D., M. Hammel, and A. Sali. 2010. FoXS: a web server for rapid computation and fitting of SAXS profiles. *Nucleic Acids Res.* 38:W540–W544. https://doi.org/10.1093/nar/gkq461.

67. Jalilehvand, F., D. Spångberg, …, M. Sandström. 2001. Hydration of the Calcium Ion. An EXAFS, Large-Angle X-ray Scattering, and Molecular Dynamics Simulation Study. *J. Am. Chem. Soc.* 123:431–441. https://doi.org/10.1021/ja001533a.

68. Pappalardo, R. R., D. Z. Caralampio, …, E. Sánchez Marcos. 2021. Hydration of Heavy Alkaline-Earth Cations Studied by Molecular Dynamics Simulations and X-ray Absorption Spectroscopy. *Inorg. Chem.* 60:13578–13587. https://doi.org/10.1021/acs.inorgchem.1c01888.

69. Mazmanian, K., C. Grauffel, …, C. Lim. 2023. Protein Ca2+-Sites Prone to Sr2+ Substitution: Implications for Strontium Therapy. *J. Phys. Chem. B.* 127:5588–5600. https://doi.org/10.1021/acs.jpcb.3c01637.

70. Llinas, P., M. Masella, …, M. H. Le Du. 2006. Structural studies of human alkaline phosphatase in complex with strontium: Implication for its secondary effect in bones. *Protein Sci.* 15:1691–1700. https://doi.org/10.1110/ps.062123806.

71. Vologzhannikova, A. A., M. P. Shevelyova, …, S. E. Permyakov. 2021. Strontium binding to alpha-parvalbumin, a canonical calcium-binding protein of the "EF-hand" family. *Biomolecules.* 11:1158. https://doi.org/10.3390/biom11081158.

72. Wang, Y., R. Tadayon, …, G. S. Shaw. 2021. Calcium binds and rigidifies the dysferlin C2A domain in a tightly coupled manner. *Biochem. J.* 478:197–215. https://doi.org/10.1042/BCJ20200773.

73. Chenal, A., J. I. Guijarro, …, D. Ladant. 2009. RTX Calcium Binding Motifs Are Intrinsically Disordered in the Absence of Calcium: Implication for Protein Secretion. *J. Biol. Chem.* 284:1781–1789. https://doi.org/10.1074/jbc.M807312200.

74. Gesztelyi, R., J. Zsuga, …, A. Tosaki. 2012. The Hill equation and the origin of quantitative pharmacology. *Arch. Hist. Exact Sci.* 66:427–438. https://doi.org/10.1007/s00407-012-0098-5.

75. Cheshmedzhieva, D., S. Ilieva, …, T. Dudev. 2021. Ca2+/Sr2+ Selectivity in Calcium-Sensing Receptor (CaSR): Implications for Strontium's Anti-Osteoporosis Effect. *Biomolecules.* 11:1576. https://doi.org/10.3390/biom11111576.

76. Kuppuraj, G., M. Dudev, and C. Lim. 2009. Factors Governing Metal-Ligand Distances and Coordination Geometries of Metal Complexes. *J. Phys. Chem. B.* 113:2952–2960. https://doi.org/10.1021/jp807972e.

77. Gifford, J. L., M. P. Walsh, and H. J. Vogel. 2007. Structures and metal-ion-binding properties of the Ca2+-binding helix-loop-helix EF-hand motifs. *Biochem. J.* 405:199–221. https://doi.org/10.1042/BJ20070255.

78. Lai, R., G. Li, and Q. Cui. 2024. Flexibility of Binding Site is Essential to the Ca2+ Selectivity in EF-Hand Calcium-Binding Proteins. *J. Am. Chem. Soc.* 146:7628–7639. https://doi.org/10.1021/jacs.3c13981.

79. Jung, H., Z. Su, …, S. Banta. 2023. Genetic Modification of Acidithiobacillus ferrooxidans for Rare-Earth Element Recovery under Acidic Conditions. *Environ. Sci. Technol.* 57:19902–19911. https://doi.org/10.1021/acs.est.3c05772.

80. Cotruvo, J. A., E. R. Featherston, …, T. N. Laremore. 2018. Lanmodulin: A Highly Selective Lanthanide-Binding Protein from a Lanthanide-Utilizing Bacterium. *J. Am. Chem. Soc.* 140:15056–15061. https://doi.org/10.1021/jacs.8b09842.

81. Choi, U. B., H. Sanabria, …, K. R. Weninger. 2019. Spontaneous Switching among Conformational Ensembles in Intrinsically Disordered Proteins. *Biomolecules.* 9:114. https://doi.org/10.3390/biom9030114.

82. Kumar, S., E. Ahmad, …, S. Gourinath. 2012. Flexibility of EF-hand motifs: structural and thermodynamic studies of Calcium Binding Protein-1 from Entamoeba histolytica with Pb2+, Ba2+, and Sr2+. *BMC Biophys.* 5:15. https://doi.org/10.1186/2046-1682-5-15.

83. Shur, O., J. Wu, …, S. Banta. 2011. Monitoring the conformational changes of an intrinsically disordered peptide using a quartz crystal microbalance. *Protein Sci.* 20:925–930. https://doi.org/10.1002/pro.625.

84. Abramson, J., J. Adler, …, J. M. Jumper. 2024. Accurate structure prediction of biomolecular interactions with AlphaFold 3. *Nature.* 630:493–500. https://doi.org/10.1038/s41586-024-07487-w.

85. Markham, K. A., G. P. Roseman, …, G. L. Millhauser. 2019. Molecular Features of the Zn2+ Binding Site in the Prion Protein Probed by 113Cd NMR. *Biophys. J.* 116:610–620. https://doi.org/10.1016/j.bpj.2019.01.005.

86. Muhammedkutty, F. N. K., M. MacAinsh, and H.-X. Zhou. 2025. Atomistic molecular dynamics simulations of intrinsically disordered proteins. *Curr. Opin. Struct. Biol.* 92:103029. https://doi.org/10.1016/j.sbi.2025.103029.

87. Khatua, P., S. Mondal, and S. Bandyopadhyay. 2019. Effects of Metal Ions on A β42 Peptide Conformations from Molecular Simulation Studies. *J. Chem. Inf. Model.* 59:2879–2893. https://doi.org/10.1021/acs.jcim.9b00098.

88. Liebschner, D., P. V. Afonine, …, P. D. Adams. 2019. Macromolecular structure determination using X-rays, neutrons and electrons: recent developments in *Phenix*. *Acta Crystallogr. D Struct. Biol.* 75:861–877. https://doi.org/10.1107/S2059798319011471.

89. Vestergaard, B., S. Sanyal, …, M. Ehrenberg. 2005. The SAXS Solution Structure of RF1 Differs from Its Crystal Structure and Is Similar to Its Ribosome Bound Cryo-EM Structure. *Mol. Cell.* 20:929–938. https://doi.org/10.1016/j.molcel.2005.11.022.

90. Round, A., E. Brown, …, C. Zubieta. 2013. Determination of the GH3.12 protein conformation through HPLC-integrated SAXS measurements combined with X-ray crystallography. *Acta Crystallogr. D.* 69:2072–2080. https://doi.org/10.1107/S0907444913019276.

Biophysical Journal, Volume *124*

Supplemental information

Ion-selective conformational stabilization of a disordered repeats-in-toxin protein domain

Alana P. Gudinas, Gatha M. Shambharkar, Marina P. Chang, Daniel Fernández, Tsutomu Matsui, and Danielle J. Mai

Supplemental Information

Ion-selective conformational stabilization of a disordered repeats-in-toxin protein domain

Alana P. Gudinas[1], Gatha M. Shambharkar[2], Marina P. Chang[2], Daniel Fernández[3], Tsutomu Matsui[4], and Danielle J. Mai[2,5,*]

[1]Department of Physics, Stanford University, Stanford, CA, USA
[2]Department of Materials Science & Engineering, Stanford University, Stanford, CA, USA
[3]Macromolecular Structure Group, Nucleus at Sarafan ChEM-H, Stanford University, Stanford, CA, USA
[4]Stanford Synchrotron Radiation Lightsource, SLAC National Accelerator Laboratory, Menlo Park, CA, USA
[5]Department of Chemical Engineering, Stanford University, Stanford, CA, USA
[*]Correspondence: djmai@stanford.edu

CONTENTS

1 Materials . . . **2**

2 Protein preparation . . . **3**
- 2.1 RTX-v DNA sequence . . . 3
- 2.2 RTX-v amino acid sequence . . . 3
- 2.3 SDS-PAGE . . . 3

3 Size exclusion chromatography with small angle X-ray scattering (SEC-SAXS) . . . **4**
- 3.1 Scattering profiles with KCl . . . 4
- 3.2 Scattering profiles with divalent cations . . . 4
- 3.3 *DENSS* reconstructions . . . 5
- 3.4 Pair distance distribution function *P(r)* fits . . . 6
- 3.5 Radius of gyration comparison across methods . . . 6
- 3.6 Protein aggregation during SEC-SAXS . . . 7
- 3.7 SEC-SAXS data collection and analysis . . . 8

4 Circular dichroism (CD) spectroscopy . . . **12**
- 4.1 Triplicate data . . . 12
- 4.2 Monovalent cation samples . . . 13
- 4.3 Hill–Langmuir fits . . . 13
- 4.4 CD Pro . . . 14
- 4.5 Sample-to-sample variation . . . 15

5 X-ray Crystallography (XRC) . . . **16**
- 5.1 Electron density maps . . . 16
- 5.2 Ligand–ion distances . . . 17
- 5.3 Turn-to-turn distances . . . 18
- 5.4 Agreement with SAXS data . . . 20
- 5.5 XRC data collection and refinement . . . 22
- 5.6 AlphaFold3 predictions . . . 23

1 MATERIALS

Table S1: Materials with vendor and catalog information.

Inorganic Salts and Buffer Reagents	Source	Identifier
Magnesium chloride hexahydrate	Thermo Fisher	CAS 7791-18-6
Calcium chloride dihydrate	Thermo Fisher	CAS 10035-04-8
Strontium chloride hexahydrate	Thermo Fisher	CAS 10025-7-4
Barium chloride dihydrate	Acros Organics	CAS 10326-27-9
Potassium chloride	Thermo Fisher	CAS 7447-40-7
Tris hydrochloride	Thermo Fisher	CAS 1185-53-1
Dithiothreitol (DTT)	Thermo Scientific	CAS 3483-12-3
**Water Purification System**		
Milli-Q Advantage A10 with BioPak Polisher, 18.2 MΩ cm	Millipore Sigma	Cat#Z00Q0V0WW
**Bacterial Strains and Competent Cells**		
NEB 5-alpha Competent E. coli (High Efficiency)	New England Biolabs	Cat#C2987H
T7 Express lysY/I^q Competent Cells	New England Biolabs	Cat#C3013I
Mix & Go! E. coli Transformation Kit and Buffer Set	Zymo Research Corporation	Cat#T3002
**Cloning**		
BamHI	New England Biolabs	Cat#R0136S
HindIII-HF	New England Biolabs	Cat#R3104S
T4 DNA Ligase	New England Biolabs	Cat#M0202L
Quick CIP	New England Biolabs	Cat#M0525S
ZymoPURE Plasmid MiniPrep Kit	Zymo Research Corporation	Cat#50-125-1483
Zymoclean Gel DNA Recovery Kit	Zymo Research Corporation	Cat#11-301
**Protein Purification and Validation**		
Nalgene™ Sterile PES Filter (0.2 μm)	Thermo Scientific	Cat#596-4520
Fisher Science Education Seamless Cellulose Dialysis Tubing (14,000 MWCO)	Thermo Scientific	Cat#S25645lH
Blue Prestained Protein Standard, Broad Range (11-250 kDa)	New England Biolabs	Cat#P7718S
HisPur™ Ni-NTA resin	Thermo Scientific	Cat#88223
Amicon® Ultra Centrifugal Filters (3 kDa MWCO)	Millipore Sigma	Cat#UFC900308
**DNA**		
Gene fragment	Twist Bioscience	N/A
pQE-9-RTXWT-C	Addgene	Cat#225964
**Crystallography**		
CrystalMation Intelli-Plates	Hampton Research	Cat#HR3-118
Morpheus I	Molecular Dimensions	Cat#MD1-47
Morpheus II	Molecular Dimensions	Cat#MD1-92
JCSG Core Suite I-IV	NeXtal	Cat#13092(4-7)
MCSG-4	Molecular Dimensions	Cat#MCSG-4
SG1 (ShotGun)	Molecular Dimensions	Cat#MD1-89
Memgold	Molecular Dimensions	Cat#MD1-41

2 PROTEIN PREPARATION

The gene encoding the RTX-v protein was flanked with restriction sites for directional cloning and purchased as gene fragments. Genes were subcloned into pQE-9 using BamHI and HindIII restriction sites, bolded in the DNA sequence below.

Protein concentration was quantified by measuring solution absorbance at 280 nm with a NanoDrop One C Spectrometer, then applying the Beer-Lambert law using a molar extinction coefficient of 18450 $M^{-1}cm^{-1}$ and a molecular mass of 19.1 kDa. Protein purity was assessed via sodium dodecyl sulfate–polyacrylamide gel electrophoresis (SDS-PAGE).

2.1 RTX-v DNA sequence

CCCCGTCACCTTTGGCTTATCAGT**GGATCC**CATATGGAGCTCGGCGCTAGCGGCAGCGCACGCGACGATGTCCTTATCGGCGATGCGGGCGCTAACGTC
CTCAACGGACTGGCTGGTAATGACGTATTATCAGGAGGGGCAGGTGACGATGTGTTATTAGGGGACGAAGGCAGTGATTTGCTGTCTGGGGATGCAGGA
AACGATGATCTGTTCGGTGGTCAGGGTGATGATACCTATCTGTTTGGGGTTGGTTACGGTCACGAC ACGATCTATGAGTCCGGCGGCGGCCACGATAC
AATCCGTATTAATGCCGGAGCAGACCAACTGTGGTTTGCGCGCCAGGGAAATGATCTTGAAATACGTATTCTCGGTACCGATGATGCGTTGACTGTTCA
TGATTGGTATCGGGACGCTGATCATAGAGTTGAAATAATTCATGC AGCGAATCAGGCTGTGGATCAAGCCGGTATTGAAAAACTGGTAGAGGCCATGG
CCCAGTACCCGGACGAATTCACTAGTCTCGAG**AAGCTT**AGATCTAGTGACATCTGGACGCTAAGACCG

2.2 RTX-v amino acid sequence

MRGSHHHHHHGSHMELGASGSARDDVLIGDAGANVLNGLAGNDVLSGGAGDDVLLGDEGSDLLSGDAGNDDLFGGQGDDTYLFGVGYGHD
TIYESGGGHDTIRINAGADQLWFARQGNDLEIRILGTDDALTVHDWYRDADHRVEIIHAANQAVDQAGIEKLVEAMAQYPDEFTSLEKLN*

2.3 SDS-PAGE

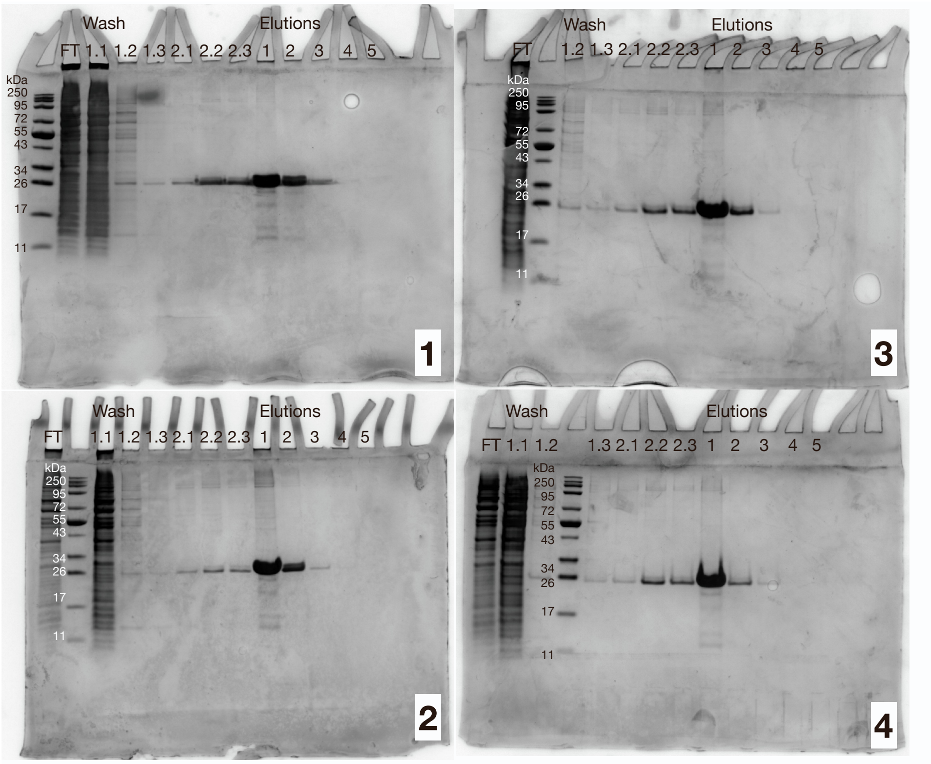

Figure S1: Representative SDS-PAGE of RTX-v fractions from flow through (FT), washes (1.1, 1.2, 1.3, 2.1, 2.2, 2.3), and elution (1-5) stages of Ni-NTA purification. Experimental conditions: 12% polyacrylamide, 200 V, 45 minutes.

3 SIZE EXCLUSION CHROMATOGRAPHY WITH SMALL ANGLE X-RAY SCATTERING (SEC-SAXS)

3.1 Scattering profiles with KCl

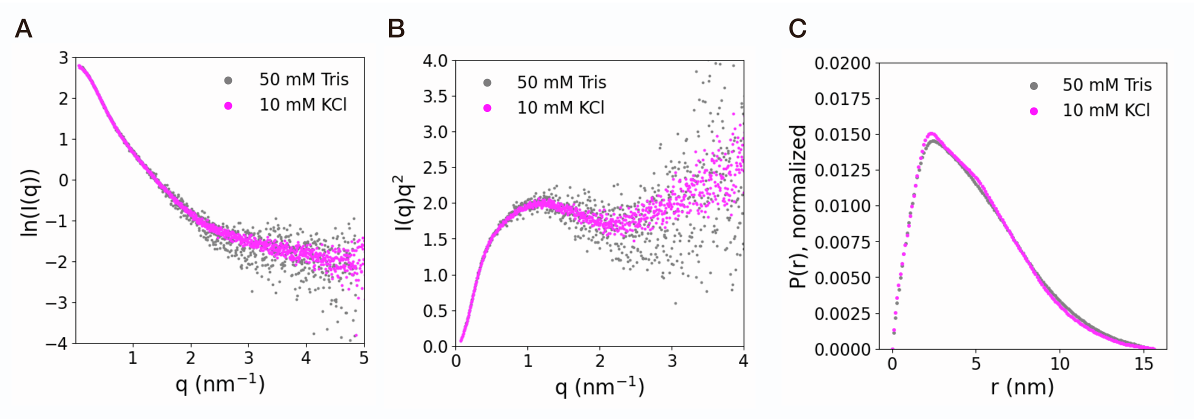

Figure S2: RTX-v exhibits identical scattering behavior when supplemented with 10 mM KCl (magenta) as in the absence of divalent cations (50 mM Tris, gray). A) Scattering profiles with 50 mM Tris, without or with 10 mM KCl. B) Kratky plots. C) $P(r)$ distributions.

3.2 Scattering profiles with divalent cations

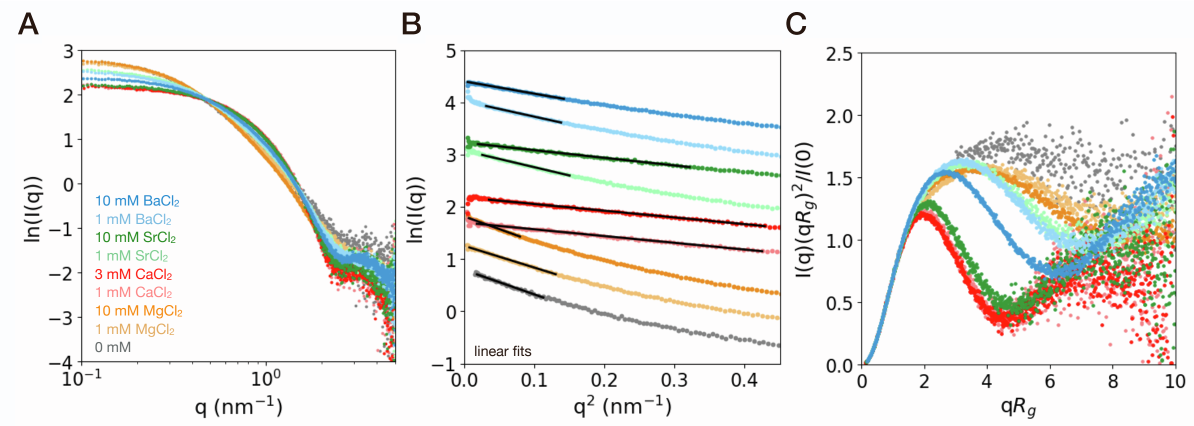

Figure S3: Scattering profiles for divalent cation conditions, overlaid to highlight differences. A) $\ln(I(q))$ vs q. Upon addition of $CaCl_2$, $SrCl_2$, and $BaCl_2$, different features emerge in the mid-q and high-q regions of the scattering profiles. The same legend is used in subsequent panels. B) Guinier plots ($\ln(I(q))$ vs q^2) with linear fits in black. Guinier analysis was applied to determine R_g and I_0 for reach sample. Steeper slopes in the low-q region of the Guinier plots correspond to larger R_g (50 mM Tris, 1 mM $MgCl_2$, 10 mM $MgCl_2$). C) Dimensionless Kratky plots for all conditions overlaid.

3.3 *DENSS* reconstructions

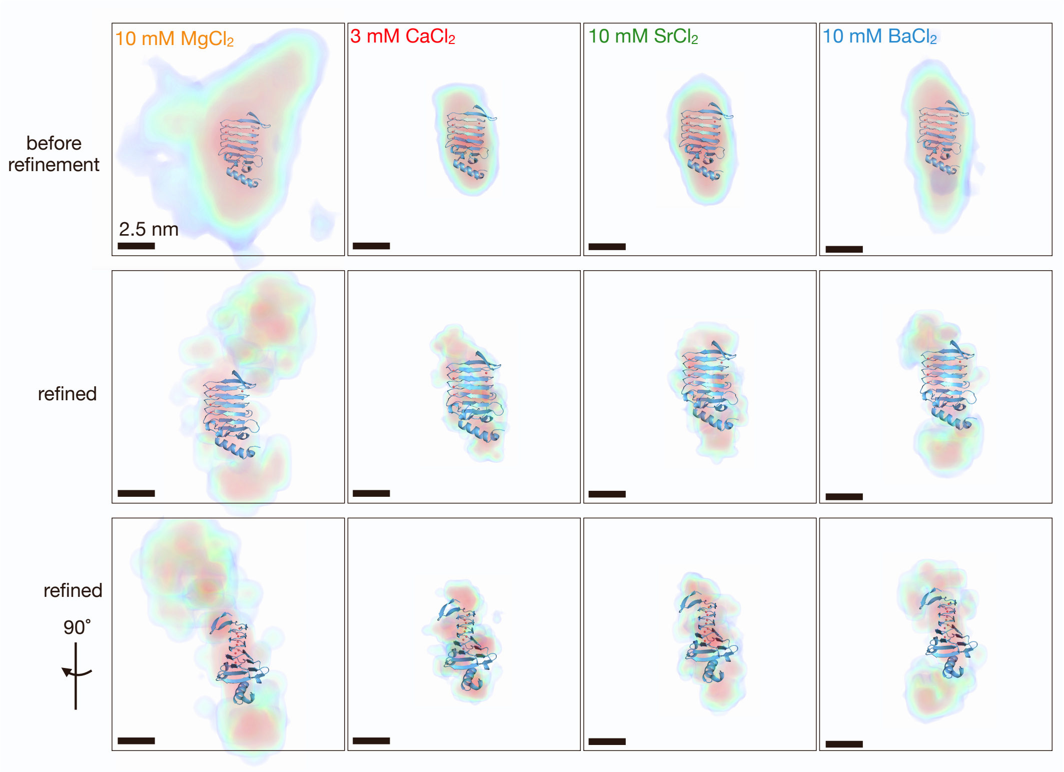

Figure S4: The *DENSS* algorithm was used to calculate 3D electron density maps from 1D SAXS data. Electron density maps are colored from lowest (blue, 2σ) to highest (red, 15σ) electron densities, where σ represents the standard deviation of the mean electron density (1). Top row: 20 *DENSS* projections were averaged and aligned with the X-ray structure of RTX-v-Ca^{2+}. Middle row: the averaged projections were refined against SAXS profiles. Bottom row: side view of refined maps, which were rotated by 90° about the vertical axis.

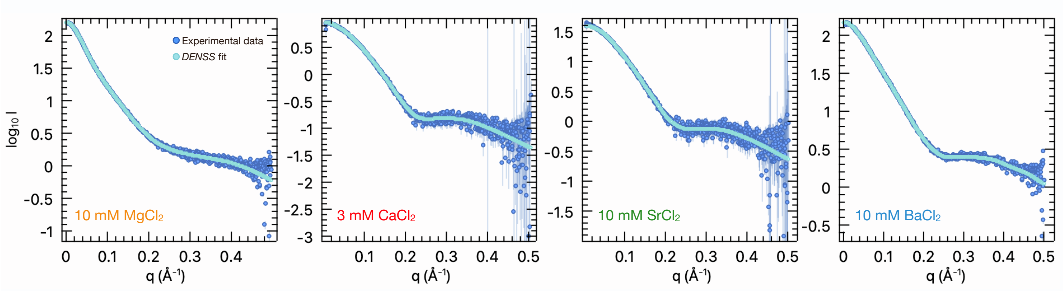

Figure S5: *DENSS* fits to experimental SAXS data. Fits to scattering data are used to refine the electron density maps.

3.4 Pair distance distribution function *P(r)* fits

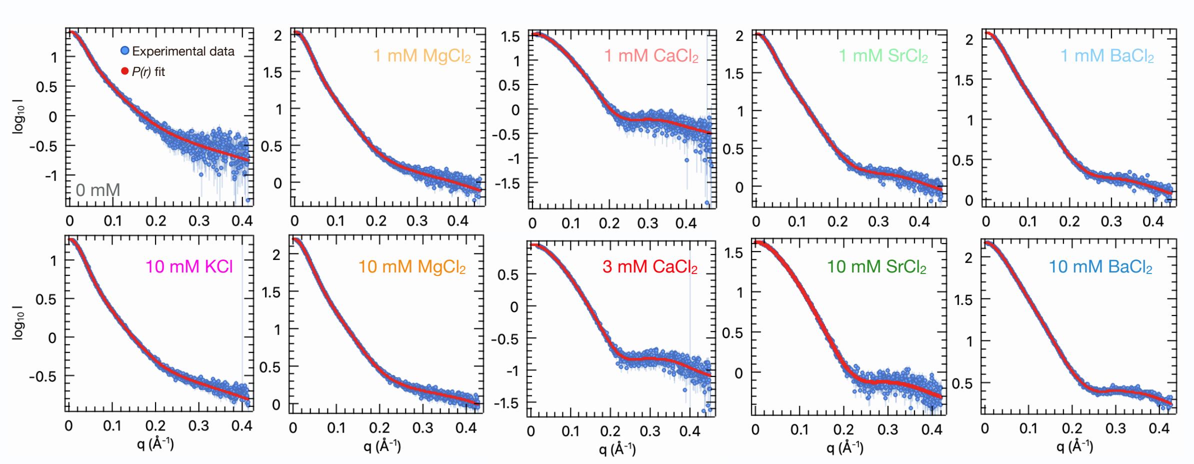

Figure S6: $P(r)$ fits to experimental SAXS data.

3.5 Radius of gyration comparison across methods

Table S2: Radii of gyration calculated by either Guinier analysis, $P(r)$, or *DENSS* reconstructions are in good agreement.

Sample	R_g (nm) Guinier	P(r)	*DENSS*
0 mM	3.8 ± 0.1	3.9	3.8 ± 0.1
1 mM $MgCl_2$	3.5 ± 0.1	3.7	3.6 ± 0.1
10 mM $MgCl_2$	3.8 ± 0.1	3.9	3.8 ± 0.1
1 mM $CaCl_2$	2.0 ± 0.1	2.0	2.0 ± 0.1
3 mM $CaCl_2$	2.0 ± 0.1	2.0	2.0 ± 0.1
1 mM $SrCl_2$	3.1 ± 0.1	3.3	3.1 ± 0.1
10 mM $SrCl_2$	2.1 ± 0.1	2.1	2.0 ± 0.1
1 mM $BaCl_2$	3.0 ± 0.1	3.2	3.1 ± 0.1
10 mM $BaCl_2$	2.7 ± 0.1	2.8	2.6 ± 0.2

3.6 Protein aggregation during SEC-SAXS

RTX-v aggregation was observed during SEC-SAXS under the following conditions: 2 mM $SrCl_2$, 3 mM $SrCl_2$, 1 mM $BaCl_2$, and 3 mM $BaCl_2$. In these cases, we determined that aggregation was not a confounding factor in extracting R_g due to agreement between R_g derived from Guinier analyses and R_g derived from $P(r)$ analyses (Table S4).

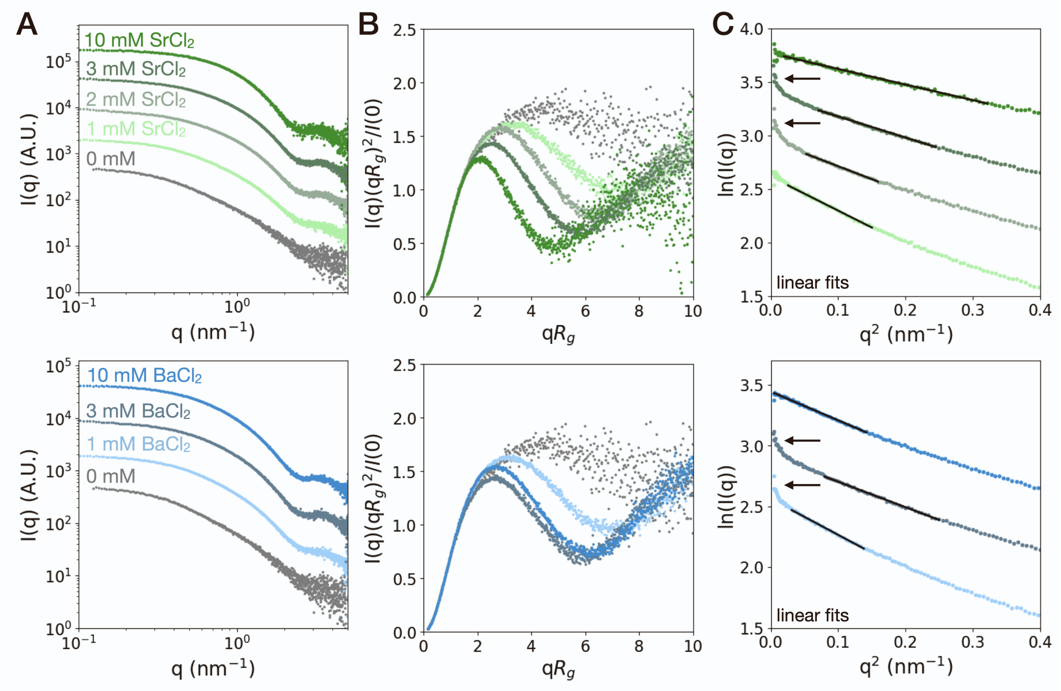

Figure S7: RTX-v adopts partially folded conformations in the presence of intermediate concentrations of $SrCl_2$ (top) and $BaCl_2$ (bottom), which induce protein aggregation. A) 1D scattering profiles. All conditions with $SrCl_2$ or $BaCl_2$ demonstrate slightly enhanced scattering in mid- and high-q regions that correspond to structure formation. Plots vertically offset for clarity. B) Kratky plots. All $SrCl_2$ and $BaCl_2$ conditions demonstrate enhanced globularity compared to the cation-free condition. C) Guinier plots with linear fits overlaid in black. An upturn in $\ln(I(q))$ at low q is characteristic of protein aggregation (indicated with arrows). Plots vertically offset for clarity.

3.7 SEC-SAXS data collection and analysis

Table S3: Data collection parameters for all SEC-SAXS samples.

**Data collection**	
Instrument	SSRL BL4-2
Type of Experiment	SEC-SAXS
Beam Current (mA)	500
Defining slits size (H mm × V mm)	0.3 × 0.30
Detector distance (m)	1.1
Detector	Pilatus3 X 1M
Beam energy (keV)	11.0
Sample cell	Quartz capillary (ID = ~1.3 mm)
Temperature (K)	295
Exposure time/frame (s)	2
Frames per SEC-SAXS data set	500
# of blank images used for averaging	50
# of sample images used for averaging	5
SEC column	Superdex 200 Increase 3.2/300
HPLC flow rate (mL/min)	0.05
Sample concentration (mg/ml)	10
**Software employed**	
Primary data reduction	*SasTool/SECPipe*
Data processing	*PRIMUS*
P(r) analysis	*GNOM*
ab initio modeling	*DENSS*

Table S4: Sample-specific data collection and structural parameters.

**Data collection**	0 mM	10 mM KCl	1 mM $MgCl_2$	10 mM $MgCl_2$	1 mM $CaCl_2$
q range ($Å^{-1}$)	0.012–0.487	0.007–0.507	0.006–0.505	0.007–0.502	0.00–0.505
Image #s used for averaging	345-349	345–349	335–339	345–349	385–389
SEC injection volume (µL)	50	30	50	50	50
Buffer	50mM Tris, pH 7.5	50mM Tris, 10 mM KCl, pH 7.5	50mM Tris, 1 mM $MgCl_2$, pH 7.5	50mM Tris, 10 mM $MgCl_2$, pH 7.5	50mM Tris, 1 mM $CaCl_2$, pH 7.5
**Guinier analysis**					
I(0) (arb. unit)	25.23 ± 0.15	18.24 ± 0.051	106.21 ± 0.26	156.39 ± 0.32	33.93 ± 0.068
R_g (Å)	37.59 ± 0.32	37.07 ± 0.15	35.35 ± 0.12	38.09 ± 0.16	19.68 ± 0.06
qR_g range	0.5–1.28	0.32–1.27	0.3–1.3	0.31–1.09	0.26–1.29
**P(r) analysis**					
I(0), Guinier (arb. unit)	25.90	18.55	108.40	157.40	34.20
R_g (Å), Guinier	40.39	39.45	37.78	39.69	20.45
I(0), P(r) (arb. unit)	25.91	18.55	108.40	157.40	34.20
R_g (Å), P(r)	40.68	39.56	37.87	39.8	20.47
D_{max} (Å)	154	156	149	163	81
q range ($Å^{-1}$)	0.001–0.412	0.008–0.415	0.009–0.454	0.009–0.423	0.013–0.466
Porod volume estimate ($Å^3$)	46881	49036	51334	74307	28888
DENSS					
Mode	Slow	-	Slow	Slow	Slow
# of reconstructions	20	-	20	20	20
χ^2	1.364 ± 0.035	-	1.607 ± 0.22	1.740 ± 0.36	1.318 ± 0.11
Resolution (FSC = 0.5)	36.4 ± 5.2	-	32.0 ± 7.6	35.5 ± 7.4	24.8 ± 5.2
Average R_g	37.62 ± 0.77	-	35.92 ± 0.73	37.98 ± 1.57	19.66 ± 0.22

cont. on next page

Data collection	3 mM $CaCl_2$	1 mM $SrCl_2$	2 mM $SrCl_2$	3 mM $SrCl_2$	10 mM $SrCl_2$
q range ($Å^{-1}$)	0.007-0.507	0.006–0.505	0.006–0.507	0.006–0.507	0.007–0.503
Image #s used for averaging	405-409	350–354	375-379	380–384	400–404
SEC injection volume (μL)	30	50	50	50	50
Buffer	50mM Tris, 1 mM $CaCl_2$, pH 7.5	50mM Tris, 1 mM $SrCl_2$, pH 7.5	50mM Tris, 2 mM $SrCl_2$, pH 7.5	50mM Tris, 3 mM $SrCl_2$, pH 7.5	50mM Tris, 10 mM $SrCl_2$, pH 7.5
**Guinier analysis**					
I(0) (arb. unit)	8.86 ± 0.016	100.96 ± 0.28	40.2 ± 0.8	42.6 ± 0.1	40.67 ± 0.089
R_g (Å)	19.64 ± 0.05	30.80 ± 0.14	26.9 ± 0.1	24.3 ± 0.1	20.92 ± 0.08
qR_g range	0.37–1.3	0.49–1.21	0.62–1.09	0.65–1.22	0.29–1.2
**P(r) analysis**					
I(0), Guinier (arb. unit)	8.95	103.10	40.73	43.76	41.22
R_g (Å), Guinier	20.52	33.06	28.37	26.50	22.36
I(0), P(r) (arb. unit)	8.95	103.20	40.74	43.76	41.22
R_g (Å), P(r)	20.58	33.32	28.61	26.70	22.43
D_{max} (Å)	88	130	115	110	101
q range ($Å^{-1}$)	0.019–0.451	0.017–0.454	0.023–0.430	0.027–0.430	0.014–0.421
Porod volume estimate ($Å^3$)	30392	46839	41148	39093	33892
DENSS					
Mode	Slow	Slow	-	-	Slow
# of reconstructions	20	20	-	-	20
χ^2	1.54 ± 0.19	1.94 ± 0.44	-	-	1.70 ± 0.21
Resolution (FSC = 0.5)	26.8 ± 4.5	29.8 ± 4.3	-	-	27.3 ± 4.8
Average R_g	19.75 ± 0.22	31.38 ± 0.93	-	-	20.29 ± 0.32

cont. on next page

Data collection	1 mM $BaCl_2$	3 mM $BaCl_2$	10 mM $BaCl_2$
q range ($Å^{-1}$)	0.007–0.503	0.006–0.507	0.007–0.503
Image #s used for averaging	370–374	385–389	390–394
SEC injection volume (μL)	50	50	50
Buffer	50mM Tris, 1 mM $BaCl_2$, pH 7.5	50 mM Tris, 3 mM $BaCl_2$, pH 7.5	50mM Tris, 10 mM $BaCl_2$, pH 7.5
**Guinier analysis**			
I(0) (arb. unit)	117.64 ± 0.26	37.7 ± 0.1	146.02 ± 0.18
R_g (Å)	29.78 ± 0.12	25.1 ± 0.1	26.59 ± 0.08
qR_g range	0.53–1.12	0.71–1.27	0.19–1.02
**P(r) analysis**			
I(0), Guinier (arb. unit)	118.90	38.78	147.00
R_g (Å), Guinier	31.27	27.14	27.89
I(0), P(r) (arb. unit)	118.90	38.78	147.00
R_g (Å), P(r)	31.50	27.31	27.93
D_{max} (Å)	131	108	117
q range ($Å^{-1}$)	0.018–0.444	0.029–0.430	0.001–0.429
Porod volume estimate ($Å^3$)	43570	39833	41295
DENSS			
Mode	Slow	-	Slow
# of reconstructions	20	-	20
χ^2	1.62 ± 0.12	-	3.73 ± 1.17
Resolution (FSC = 0.5)	27.7 ± 1.2	-	27.8 ± 5.0
Average R_g	30.88 ± 0.56	-	25.7 ± 1.26

4 CIRCULAR DICHROISM (CD) SPECTROSCOPY

4.1 Triplicate data

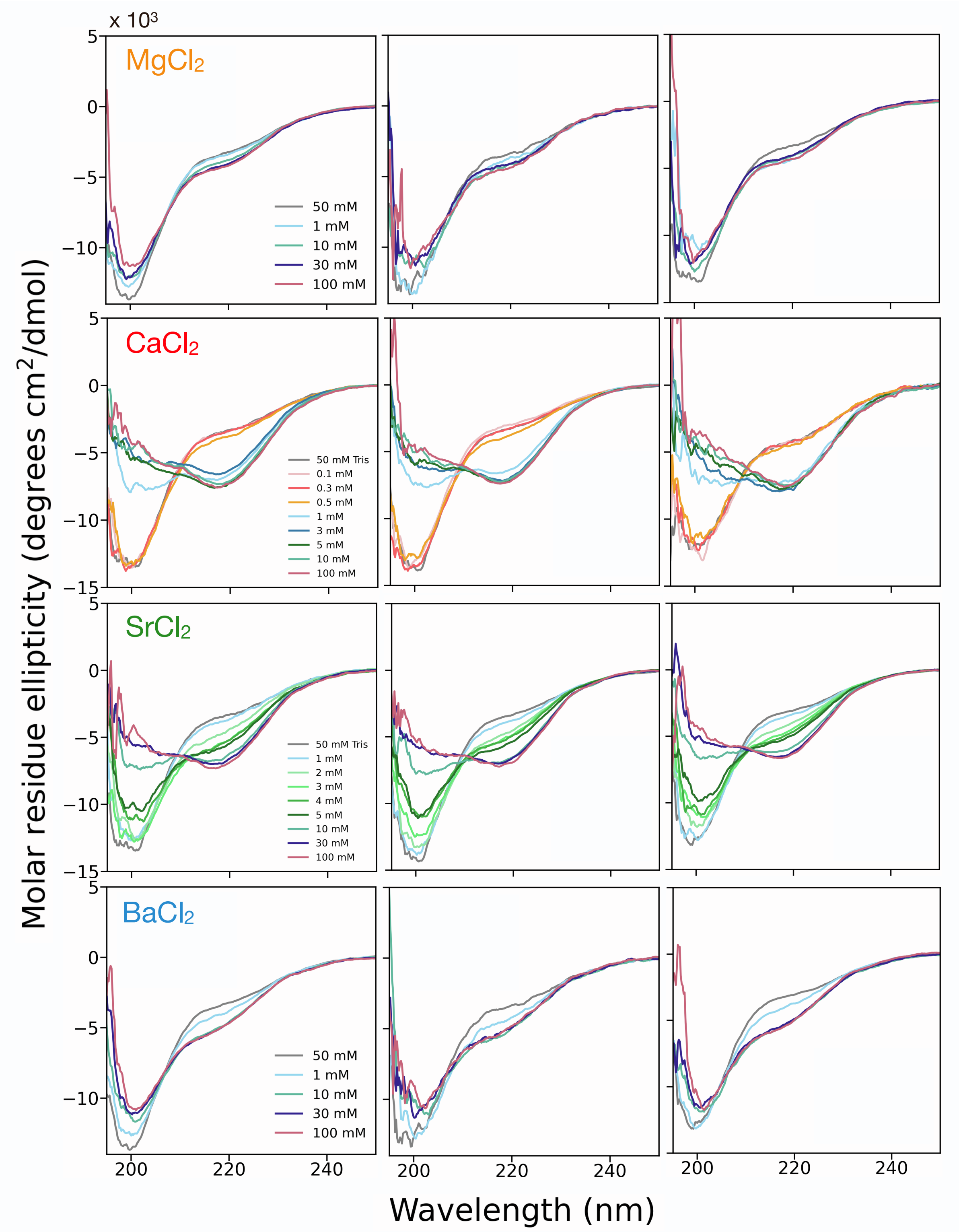

Figure S8: Triplicate CD spectra for each group II ion condition. Each column corresponds to a distinct biological sample. Different noise levels across replicates are attributed to aging of the UV lamp in the CD spectrometer.

4.2 Monovalent cation samples

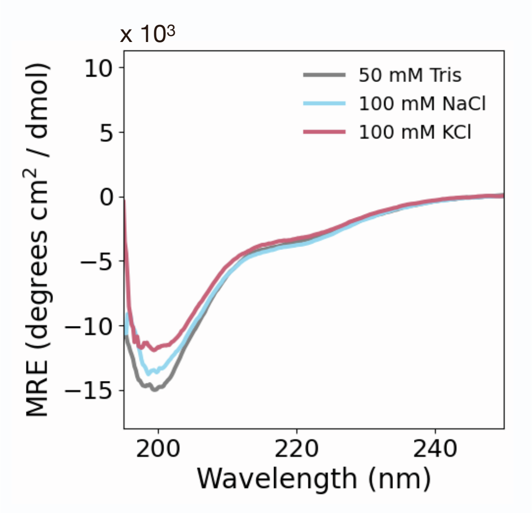

Figure S9: RTX-v does not form β-sheets in the presence of K^+ or Na^+ ions. CD spectra of RTX-v with 100 mM KCl and 100 mM NaCl overlaid with 50 mM Tris.

4.3 Hill–Langmuir fits

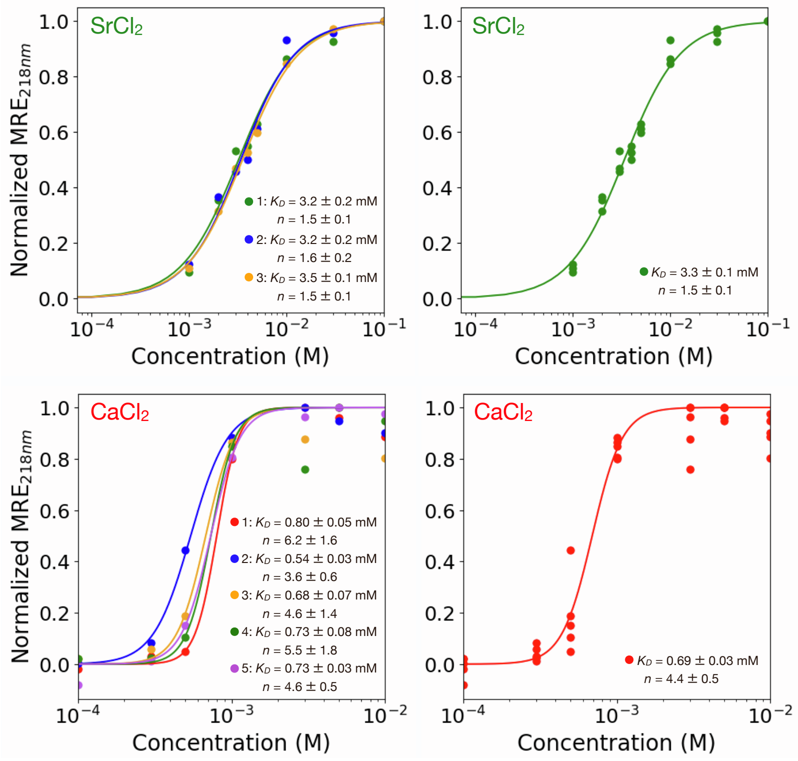

Figure S10: Hill–Langmuir fits to normalized $MRE_{218\text{ nm}}$ upon titration with $CaCl_2$ or $SrCl_2$ (2). Fits to the Hill equation were applied to either individual replicates (left) or the combined data set (right). K_D and n reported in the main text are fit from combined data sets. The reported errors on K_D and n are the root mean square errors derived from the Hill fits to the combined data sets.

Table S5: Parameters computed from Hill fits.

**Sample**	K_D **(mM)**	n
RTX-v-Ca^{2+}	0.69 ± 0.03	4.4 ± 0.5
RTX-v-Sr^{2+}	3.3 ± 0.1	1.5 ± 0.1

4.4 CD Pro

Spectral deconvolution was performed from 200 nm to 250 nm with CDPro software using the reference set SPD48, which is the largest available reference set that includes denatured proteins (3). The results from CDSSTR, CONTIN/LL, and SELCON3 methods from each replicate were normalized and averaged to facilitate quantitative comparisons.

Table S6: Structural components of each sample, listed as a percentage of the total structure (%). Errors represent the standard deviation between replicates.

**Sample**	Helix	Sheet	Turn	Unstructured
50 mM Tris	4.3 ± 0.8	15.3 ± 1.8	10.3 ± 0.7	70.1 ± 3.1
1 mM $MgCl_2$	5.5 ± 0.1	16.3 ± 1.5	11.4 ± 1.0	66.7 ± 2.4
100 mM $MgCl_2$	5.6 ± 0.2	16.2 ± 0.6	12.1 ± 0.2	66.1 ± 1.0
1 mM $CaCl_2$	7.4 ± 0.8	25.9 ± 0.6	18.6 ± 0.3	48.2 ± 0.4
100 mM $CaCl_2$	7.7 ± 0.7	28.3 ± 0.6	20.2 ± 0.6	43.9 ± 0.3
1 mM $SrCl_2$	5.0 ± 0.6	16.3 ± 1.3	10.8 ± 0.6	67.9 ± 1.6
100 mM $SrCl_2$	7.0 ± 0.6	28.6 ± 0.2	19.9 ± 0.1	44.6 ± 0.4
1 mM $BaCl_2$	5.3 ± 0.6	15.9 ± 0.4	11.0 ± 0.3	67.8 ± 1.0
100 mM $BaCl_2$	6.1 ± 0.7	21.1 ± 3.7	16.0 ± 2.0	56.8 ± 6.3

4.5 Sample-to-sample variation

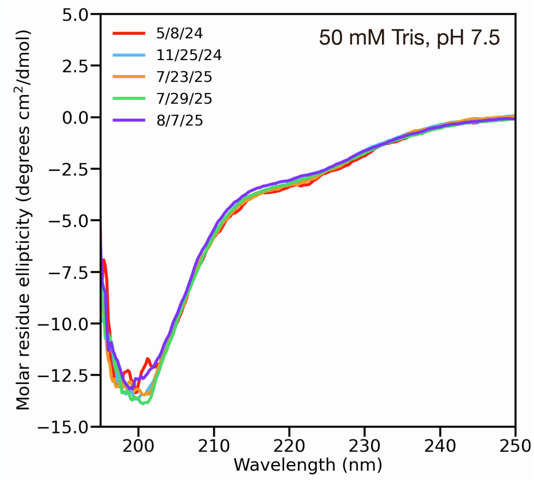

Figure S11: Sample-to-sample variation between circular dichroism experiments with RTX-v, measured in 50 mM Tris, pH 7.5. Each sample is a distinct biological replicate. Differences in noise and absolute absorbance of the spectra are attributed to aging of the UV lamp in the CD spectrometer.

Table S7: Structural components of each sample replicate in 50 mM Tris, listed as a percentage of the total structure (%). The standard deviation of the mean of all replicates is given by σ.

**Sample**	Helix	Sheet	Turn	Unstructured
5/8/24	3.6	13.2	9.4	74.0
11/25/24	3.6	13.2	9.6	74.0
7/23/25	3.8	16.7	10.8	68.8
7/29/25	5.3	15.9	10.6	68.2
8/7/25	5.3	17.6	11.2	65.9
mean ± σ	4.3 ± 0.8	15.3 ± 1.8	10.3 ± 0.7	70.1 ± 3.1

5 X-RAY CRYSTALLOGRAPHY (XRC)

5.1 Electron density maps

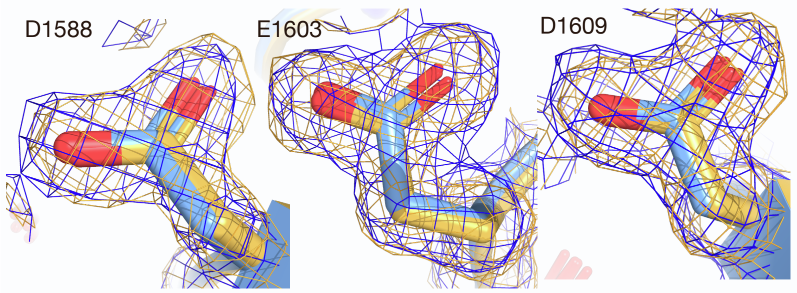

Figure S12: Overlaid 2Fo-Fc electron density maps for RTX-v-Ca^{2+} (blue) and RTX-v-Sr^{2+} (yellow) contoured at 1.8 Å from each atom. Selected residues Asp1588, Glu1603, and Asp1609 coordinate Ca^{2+}(7) or Sr^{2+}(7). Subtle shifts in side chain positions occur upon Sr^{2+} binding. Electron density maps were aligned using phenix.superpose_maps in *Phenix* (4).

5.2 Ligand–ion distances

Table S8: Distances between coordinating ligands and Ca^{2+}or Sr^{2+} in X-ray structures for RTX-v-Ca^{2+} or RTX-v-Sr^{2+}, respectively (Å). The ions are numbered in order from N(1)- to C(8)-termini. Oδ1 refers to the double-bonded oxygen atom in aspartic acid side chain carboxyls, and Oδ2 refers to the single-bonded oxygen. Oϵ1 refers to the double-bonded oxygen atom in glutamic acid side chain carboxyls, and Oϵ2 refers to the single-bonded oxygen. Each average difference was computed from the sum of all differences between Ca^{2+}–ligand and Sr^{2+}–ligand distances, divided by the number of coordinating ligands.

Ion	Ligand	Ca^{2+}	Sr^{2+}	Avg. difference
1	Asp1539O	2.4	2.4	+ 0.03 Å
	Gl1541O	2.3	2.4	
	Asn1543Oδ1	2.4	2.4	
	Gly1556O	2.3	2.4	
	Ala1558O	2.5	2.4	
	Asp1561Oδ2	2.8	2.9	
	Asp1561Oδ1	2.4	2.4	
2	Gly1557O	2.4	2.4	+ 0.03 Å
	Gly1559O	2.3	2.4	
	Asp1561Oδ2	2.3	2.3	
	Gly1574O	2.4	2.4	
	Ala1576O	2.4	2.3	
	Asp1579Oδ2	2.8	3.0	
	Asp1579Oδ1	2.5	2.5	
3	Asp1575O	2.3	2.3	- 0.02 Å
	Gly1577O	2.2	2.3	
	Asp1579Oδ2	2.4	2.3	
	Tyr1596O	2.3	2.2	
	Asp1599Oδ2	2.5	2.5	
	Asp1599Oδ1	2.3	2.3	
4	Ser1530O	2.3	2.4	+ 0.01 Å
	Arg1532O	2.3	2.3	
	Asp1534Oδ2	2.3	2.3	
	Gly1547O	2.3	2.3	
	Ala1549O	2.3	2.3	
	Asp1552Oδ2	2.8	2.9	
	Asp1552Oδ1	2.4	2.3	
5	Leu1548O	2.4	2.4	+ 0.03 Å
	Gly1550O	2.4	2.5	
	Asp1552Oδ2	2.3	2.4	
	Gly1565O	2.3	2.3	
	Glu1567O	2.3	2.3	
	Asp1570Oδ1	3.1	3.0	
	Asp1570Oδ2	2.4	2.5	
6	Asp1566O	2.3	2.5	+ 0.07 Å
	Gly1568O	2.4	2.5	
	Asp1570Oδ2	2.3	2.4	
	Gly1583O	2.4	2.5	
	Gln1585O	2.3	2.4	
	Asp1588Oδ2	3.0	2.9	
	Asp1588Oδ1	2.5	2.5	

7	Gly1584O	2.4	2.5	+ 0.1 Å
	Gly1586O	2.3	2.5	
	Asp1588Oδ2	2.3	2.5	
	Glu1603Oϵ2	2.6	2.6	
	Glu1603Oϵ1	2.5	2.6	
	Gly1605O	2.3	2.4	
	Asp1609Oδ1	2.6	2.6	
8	Gly1606O	2.3	2.3	0.00 Å
	Asp1609Oδ2	2.2	2.4	
	H2O1	2.4	2.4	
	H2O2	2.5	2.4	
	Arg1652O	2.4	2.4	
	Glu1654Oϵ2	2.5	2.4	
	Glu1654Oϵ1	2.6	2.6	

5.3 Turn-to-turn distances

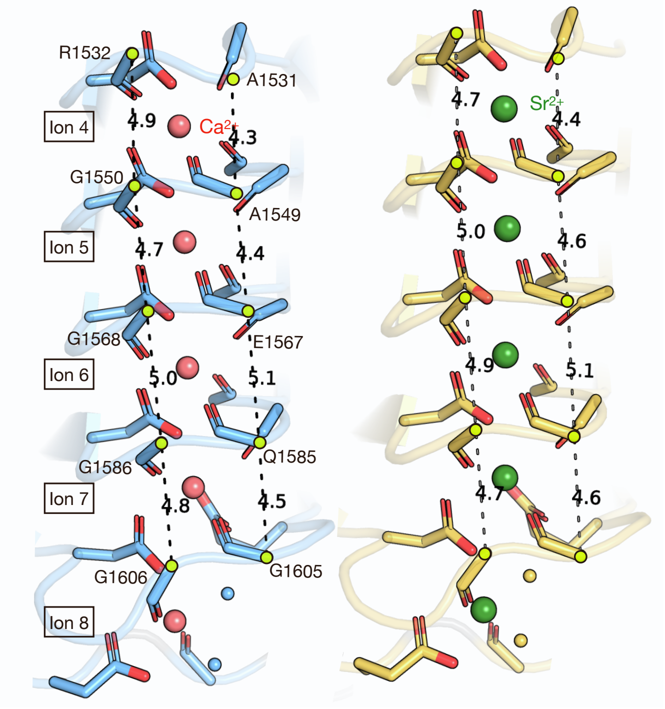

Figure S13: Side view of RTX-v-Ca^{2+} (blue) and RTX-v-Sr^{2+} (yellow), where outlined lime circles denote the α-carbon positions used to measure turn-to-turn distances. Two pairs of α-carbon atoms were selected for every turn.

Table S9: Turn-to-turn distances surrounding each ion for RTX-v-Ca^{2+} and RTX-v-Sr^{2+} (Å). Distances between two pairs of α-carbons above and below each ion were measured. Each average difference was computed from the sum of differences in α-carbon distances between the RTX-v-Ca^{2+} and RTX-v-Sr^{2+} structures divided by two.

Ion	Residues	Ca^{2+}	Sr^{2+}	Avg. difference
1	Ala1540-Ala1558	4.2	4.3	0.0 Å
	Gly1541-Gly1559	4.8	4.7	
2	Ala1558-Ala1576	4.5	4.6	+ 0.1 Å
	Gly1559-Gly1577	4.5	4.6	
3	Ala1576-Tyr1596	4.3	4.3	+ 0.05 Å
	Gly1577-Gly1597	5.3	5.4	
4	Ala1531-Ala1549	4.3	4.4	- 0.05 Å
	Arg1532-Gly1550	4.9	4.7	
5	Ala1549-Glu1567	4.4	4.6	+ 0.25 Å
	Gly1550-Gly1568	4.7	5.0	
6	Glu1567-Gln1585	5.1	5.1	- 0.05 Å
	Gly1568-Gly1586	5.0	4.9	
7	Gln1585-Gly1605	4.5	4.6	0.0 Å
	Gly1586-Gly1606	4.8	4.7	

5.4 Agreement with SAXS data

We used the FoXS software web interface to fit the theoretical scattering curves from protein crystal structures to corresponding SAXS profiles (5). We selected the 3 mM $CaCl_2$ and 10 mM $SrCl_2$ scattering profiles for comparison with the protein structure, since RTX-v is most likely to be folded in these solution conditions. For both scattering profiles, we used the RTX-v-Ca^{2+} (PDB: 9P0C) structure as the model input. RTX-v-Sr^{2+} (PDB: 9P0D) was not used as a model because the crystal structure is missing a total of 23 residues at the N- and C-termini, whereas the RTX-v-Ca^{2+} structure lacks only 7 residues. The missing residues are cloning artifacts, which are present in the protein samples used for SEC-SAXS. To achieve accurate fits with FoXS or CRYSOL, it is critical for the input protein structure or model to include all residues present in the sample measured during SAXS (6). Given the similarity between the RTX-v-Ca^{2+} and RTX-v-Sr^{2+} structures, we consider RTX-v-Ca^{2+} as an appropriate model to fit both the 3 mM $CaCl_2$ and 10 mM $SrCl_2$ scattering profiles. As a second comparison, we used AlphaFold3 (AF3) to predict a protein model from the full RTX-v sequence (7). Fits computed from both the RTX-v-Ca^{2+} structure and AF3 prediction are shown in Figure S14 for each scattering profile.

We find that fits generated from both the RTX-v-Ca^{2+} structure and AF3 prediction agree well with the scattering profiles. Notably, fits generated from AF3 predictions show better agreement with both scattering profiles (given by χ^2 values). We attribute better agreement with the AF3 prediction to its inclusion of all residues found in the SAXS protein sample.

χ^2 values for both fits to the 10 mM $SrCl_2$ scattering profile are larger than for fits to the 3 mM $CaCl_2$ profile. We attribute poorer fits to the 10 mM $SrCl_2$ data to the possible presence of multiple conformations of RTX-v. RTX-v has lower affinity to Sr^{2+} than Ca^{2+}, so it is possible that some unfolded or partially folded conformations remain at 10 mM $SrCl_2$. The presence of multiple protein conformations in solution SAXS can result in worse fits to protein models, which assume a single conformation (8).

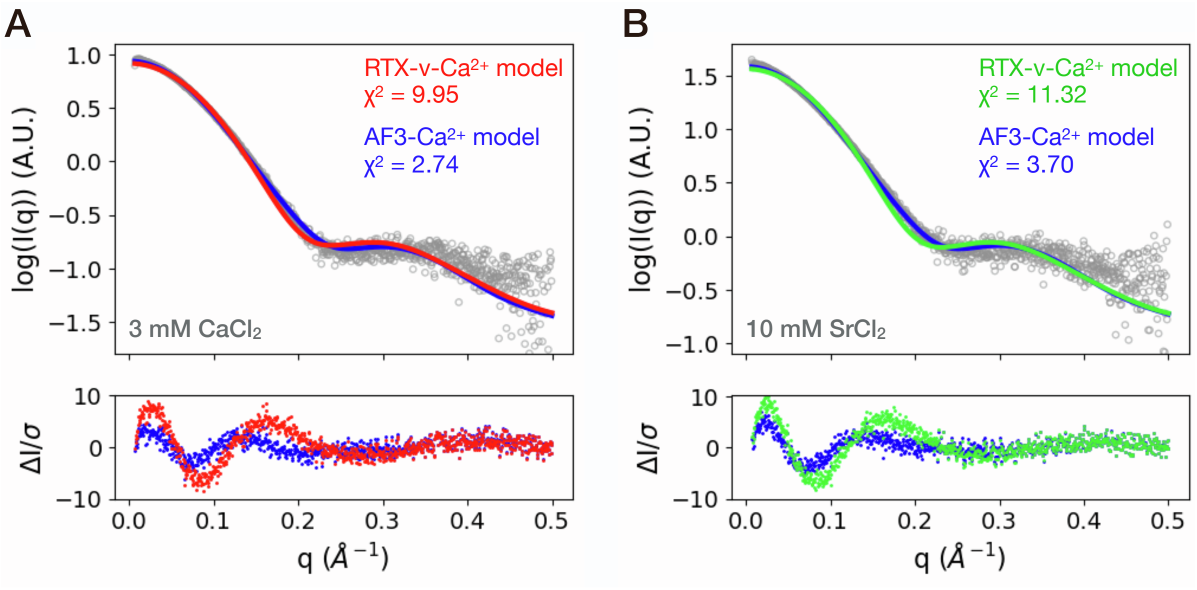

Figure S14: Theoretical scattering profiles generated from RTX-v-Ca^{2+} and the AlphaFold3 prediction agree well with experimental data collected at 3 mM $CaCl_2$ and 10 mM $SrCl_2$. Fits generated from AF3 predictions show better agreement with both experimental scattering profiles, given by χ^2 values. A) Top: fits generated from the RTX-v-Ca^{2+} structure and AF3 prediction compared to the experimental 3 mM $CaCl_2$ scattering profile. Bottom: residuals from the fits. B) Top: fits generated from the RTX-v-Ca^{2+} structure and AF3 prediction compared to the experimental 10 mM $SrCl_2$ scattering profile. χ^2 values for both fits are larger than for fits to the 3 mM $CaCl_2$ profile. We attribute the poorer fits to the 10 mM $SrCl_2$ data to the possible presence of multiple conformations of RTX-v at 10 mM $SrCl_2$.

5.5 XRC data collection and refinement

Table S10: Data collection parameters and structure refinement results for RTX-v-Ca^{2+} and RTX-v-Sr^{2+}.

**Data collection**	RTX-v-Ca^{2+}	RTX-v-Sr^{2+}
X-ray source	SSRL BL9-2	SSRL BL12-2
Wavelength (Å)	0.97946	0.76910
Space group	$P2_1$	I222
Cell dimensions		
a, b, c (Å)	37.722, 66.759, 59.072	62.420, 72.870, 73.755
α, β, γ (°)	90.00, 93.22, 90.00	90.00, 90.00, 90.00
Matthews coefficient (Å^3/Da)[a]	2.07	2.61
Solvent content (%)	40.5	52.8
Wilson B value (Å^2)	17.0	15.6
Mosaicity	0.31	0.14
Resolution (Å)[b]	37.7(1.7)	47.65(1.50)
R_{merge}[c]	0.097(0.991)	0.303(1.860)
I / σI ratio[d]	7.2(1.5)	5.0(1.2)
Completeness (%)[e]	98.4(98.2)	98.6(94.2)
Reflections (total/unique)	121,050(31,714)	354,965(26,872)
Redundancy[f]	3.8(3.8)	13.2(12.0)
**Refinement**		
Resolution (Å)	30.66-1.70	30.95-1.50
No. reflections/test set	30,046/1,581	25,532/1,314
R_{work}/R_{free}[g]	16.7/20.9	17.1/20.2
Mean B value (Å^2)	24.9	21.5
F_{obs}-F_{calc} correlation[h]	0.97	0.97
No. atoms		
Protein	1,281 (chain A), 1,262 (chain B)	1,151
Ligand/ion	33 (16x Ca^{2+})/(4x Zn^{2+})/(1x Cl^-)/(2x glycerol)	36 (8x Sr^{2+})/(1x Cl^-, 1x Na^+, 1x formaldehyde, 1x glycerol, 2x tris)
Water	187	111
B-factors		
Protein	22.2 (chain A)/22.0 (B)	17.4 (chain A)
Ligand/ion	17.2 (Ca^{2+} chain A)/15.7 (Ca^{2+} chain B)/21.1 (Zn^{2+}) 59.8 (Cl^-)/45.2 (glycerol)	12.1 (Sr^{2+}), 34.9 (Cl^-), 40.8 (Na^+), 24.1 (formaldehyde), 49.9 (glycerol), 43.3 (tris)
Water	30.4	35.4
Deviation from ideality (Rmsd values)	0.020 Å (bond length), 1.945°(bond angle)	0.022 Å (bond length), 2.424°(bond angle)
Ramachandran statistics[i]		
Most favored/allowed regions (%)	99.0 (278 over 281)	100.0 (127 over 127)
Disallowed regions (%)	1.0 (3 over 281)	0.0 (0 over 127)
PDB code	9P0C	9P0D

[a] Ratio of the volume of the asymmetric unit to the molecular weight of all protein in the asymmetric unit.
[b] Value in parentheses is for the highest-resolution shell: 1.70-1.73 Å (RTX-Ca^{2+}) and 1.50-1.58 Å (RTX-v-Sr^{2+}).
[c] Reliability factor for symmetry-related reflections calculated as: $R_{merge} = \Sigma_{hkl}\Sigma j{=}1$ to N | $I_{hkl} - I_{hkl}$ (j) | / Σ_{hkl} $\Sigma j{=}1$ to N I_{hkl} (j), where N is the redundancy of the data. The cumulative value at the highest-resolution shell is in parentheses.
[d] Ratio of mean intensity to the mean standard deviation of the intensity over the entire resolution range.
[e] Fraction of measured reflections to possible observations at the resolution range.
[f] Number of measurements of individual, symmetry unique reflections.
[g] Average deviation between the observed and calculated structure factors calculated as: $R_{work} = \Sigma_{hkl}||F_{obs}| - |F_{calc}|| / \Sigma_{hkl}|F_{obs}|$, where the F_{obs} and F_{calc} are the observed and calculated structure factor amplitudes of reflection hkl. R_{free} is equal to R_{factor} but for a randomly selected 5.0 % subset of the total reflections that were held aside throughout refinement for cross-validation.
[h] Correlation coefficient between observed and calculated structure factor amplitudes.
[i] According to Procheck for non-glycine and non-proline residues.

5.6 AlphaFold3 predictions

To investigate whether AlphaFold3 (AF3) can resolve ion-dependent RTX-v conformations, we predicted RTX-v structures in the presence of different ions (7). AF3 was developed to predict biomolecular interactions, including ionic interactions. Currently, the only ion ligands supported in AF3 are Mg^{2+}, Zn^{2+}, Cl^{-}, Ca^{2+}, Na^{+}, Mn^{2+}, K^{+}, Fe^{3+}, Cu^{2+}, and Co^{2+}. We tested AF3 predictions of RTX-v structure under the conditions depicted in Figure S15. We compared conditions with either 2 or 8 ion ligands to test whether AF3 can predict partially folded structures. Additionally, we included Mn^{2+} and Co^{2+} to probe whether AF3 may predict alternative RTX-v structures. Our results demonstrate that based on available crystallography data of RTX-v, AF3 is unable to predict disordered or partially folded states. All ionic conditions, including the ion-free condition, produced similar structure predictions with high pLDDT scores that closely resemble the RTX-v-Ca^{2+} structure. The predicted local distance difference test (pLDDT) score indicates local confidence in the predicted structure.

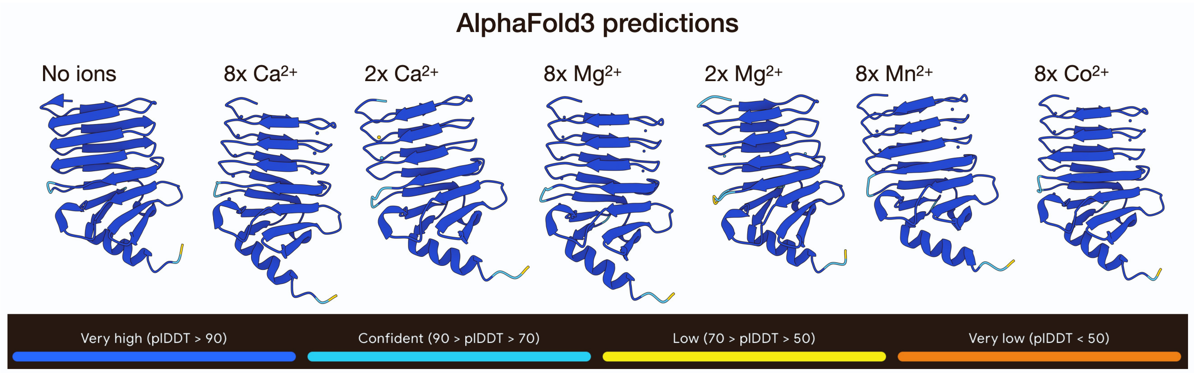

Figure S15: AlphaFold3 predictions of RTX-v structures in the presence of different ions. Under all conditions, AF3 predict similar structures with high pLDDT sctores that closely resemble the RTX-v-Ca^{2+} structure.

REFERENCES

1. Grant, T. D., 2018. Ab initio electron density determination directly from solution scattering data. *Nature Methods* 15:191–193. DOI: 10.1038/nmeth.4581.

2. Gesztelyi, R., J. Zsuga, A. Kemeny-Beke, B. Varga, B. Juhasz, and A. Tosaki, 2012. The Hill equation and the origin of quantitative pharmacology. *Archive for History of Exact Sciences* 66:427–438. DOI: 10.1007/s00407-012-0098-5.

3. Sreerama, N., and R. W. Woody, 2000. Estimation of Protein Secondary Structure from Circular Dichroism Spectra: Comparison of CONTIN, SELCON, and CDSSTR Methods with an Expanded Reference Set. *Analytical Biochemistry* 287:252–260. DOI: 10.1006/abio.2000.4880.

4. Liebschner, D., P. V. Afonine, M. L. Baker, G. Bunkóczi, V. B. Chen, T. I. Croll, B. Hintze, L.-W. Hung, S. Jain, A. J. McCoy, N. W. Moriarty, R. D. Oeffner, B. K. Poon, M. G. Prisant, R. J. Read, J. S. Richardson, D. C. Richardson, M. D. Sammito, O. V. Sobolev, D. H. Stockwell, T. C. Terwilliger, A. G. Urzhumtsev, L. L. Videau, C. J. Williams, and P. D. Adams, 2019. Macromolecular structure determination using X-rays, neutrons and electrons: recent developments in *Phenix*. *Acta Crystallographica Section D* 75:861–877. DOI: 10.1107/S2059798319011471.

5. Schneidman-Duhovny, D., M. Hammel, and A. Sali, 2010. FoXS: a web server for rapid computation and fitting of SAXS profiles. *Nucleic Acids Research* 38:W540–W544. DOI: 10.1093/nar/gkq461.

6. Vestergaard, B., S. Sanyal, M. Roessle, L. Mora, R. H. Buckingham, J. S. Kastrup, M. Gajhede, D. I. Svergun, and M. Ehrenberg, 2005. The SAXS Solution Structure of RF1 Differs from Its Crystal Structure and Is Similar to Its Ribosome Bound Cryo-EM Structure. *Molecular Cell* 20:929–938. DOI: 10.1016/j.molcel.2005.11.022.

7. Abramson, J., J. Adler, J. Dunger, R. Evans, T. Green, A. Pritzel, O. Ronneberger, L. Willmore, A. J. Ballard, J. Bambrick, S. W. Bodenstein, D. A. Evans, C.-C. Hung, M. O'Neill, D. Reiman, K. Tunyasuvunakool, Z. Wu, A. Žemgulytė, E. Arvaniti, C. Beattie, O. Bertolli, A. Bridgland, A. Cherepanov, M. Congreve, A. I. Cowen-Rivers, A. Cowie, M. Figurnov, F. B. Fuchs, H. Gladman, R. Jain, Y. A. Khan, C. M. R. Low, K. Perlin, A. Potapenko, P. Savy, S. Singh, A. Stecula, A. Thillaisundaram,

C. Tong, S. Yakneen, E. D. Zhong, M. Zielinski, A. Žídek, V. Bapst, P. Kohli, M. Jaderberg, D. Hassabis, and J. M. Jumper, 2024. Accurate structure prediction of biomolecular interactions with AlphaFold 3. *Nature* 630:493–500. DOI: 10.1038/s41586-024-07487-w.

8. Round, A., E. Brown, R. Marcellin, U. Kapp, C. S. Westfall, J. M. Jez, and C. Zubieta, 2013. Determination of the GH3.12 protein conformation through HPLC-integrated SAXS measurements combined with X-ray crystallography. *Acta Crystallographica Section D* 69:2072–2080. DOI: 10.1107/S0907444913019276.
